# Computational Design and Theoretical Evaluation of Novel Heterocyclic Energetic Materials Based on 5‐H‐Imidazo‐[4,5‐c]‐Pyridazine and 1,2,4‐Triazolo‐[4,3‐a]‐Pyrazine Scaffolds

**DOI:** 10.1002/jcc.70490

**Published:** 2026-09-03

**Authors:** Luciana Amorim da Silva, Nathália Magalhães Paixão Rosa, Danillo Fernando Vianna Cantini, Aline Cardoso Anastácio

**Affiliations:** ^1^ Chemical Engineering Department Military Institute of Engineering Rio de Janeiro Rio de Janeiro Brazil; ^2^ Institute of Chemistry Federal Rural University of Rio de Janeiro Seropédica Rio de Janeiro Brazil; ^3^ Institute of Energetic Materials, Faculty of Chemical Technology University of Pardubice Pardubice Czech Republic

**Keywords:** aromaticy, bond order analysis, detonation properties, DFT, energetic materials, nitrogen heterocycles, substituent effects

## Abstract

Novel energetic materials based on 5‐H‐imidazo‐[4,5‐c]‐pyridazine and 1,2,4‐triazolo‐[4,3‐a]‐pyrazine scaffolds, modified with explosophore groups (—NO_2_, —NHNO_2_, —N_3_, and —ONO_2_), were theoretically designed and evaluated by DFT at the B3LYP/def2‐TZVPP // B3LYP/6‐311G(d,p) level. Nitramino derivatives exhibited the highest chemical stability based on HOMO‐LUMO gap analysis. Heat of formation followed the trend —N_3_ > —NO_2_ > —NHNO_2_ > —ONO_2_. Most imidazopyridazine derivatives exceeded RDX density, while all surpassed TNT. All derivatives showed superior detonation parameters over TNT, with 18 exceeding RDX performance. Impact sensitivity analysis revealed favorable safety profiles, with all di‐ and tri‐substituted nitramines less sensitive than RDX. Mayer bond order analysis of all 88 compounds identified the trigger bonds for each compound and class, revealing distinct lability ranges for each explosophore:O—NO_2_ (0.490–0.728), C—NO_2_ (0.737–0.789), HN—NO_2_ (0.784–0.892), C—NHNO_2_ (0.917–0.944), and C—N_3_ (1.018–1.102). Aromaticity analysis ($\text{NICS}{\left(1\right)}_{\mathrm{zz}}$) showed that explosophore substitution systematically reduces aromatic character relative to the unsubstituted scaffolds, a trend correlated with molecular electrostatic potential (MEP) redistribution and associated with higher density, heat of detonation, detonation velocity, and detonation pressure. After a combined analysis of detonation performance, impact sensitivity profiles, trigger bond lability, and bond dissociation enthalpies, three compounds (IA345, IA356, and TA356) emerged as the most promising candidates, exhibiting heat of formation > 303.7 kJ mol^−1^, oxygen balance > −28.2%, density > 1.85 g cm^−3^, detonation velocity > 8.76 km s^−1^, and detonation pressure > 33.17 GPa.

## Graphical abstract

Eighty‐eight novel energetic compounds based on 5‐H‐imidazo‐[4,5‐c]pyridazine and 1,2,4‐triazolo‐[4,3‐a]pyrazine scaffolds, functionalized with nitro, nitramino, azido, and nitrate ester groups, were theoretically evaluated by DFT. Nine candidates surpassed RDX in density and detonation performance while maintaining acceptable impact sensitivity. Both heterocyclic frameworks are established as promising platforms for next‐generation energetic materials, enabling preliminary screening of critical performance and safety parameters while minimizing synthesis hazards.

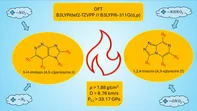

## 1 Introduction

Novel energetic materials development requires balancing performance with safety, targeting high density, superior detonation properties, and thermal‐chemical stability [1] Nitrogen heterocycles offer promising frameworks due to higher formation enthalpies and improved densities versus carbocyclic analogs [2, 3]. These structures also produce environmentally friendly dinitrogen gas upon combustion [4].

Fused heterocyclic systems provide additional advantages through coplanar frameworks and large conjugate systems [5, 6, 7], enhancing thermal stability and reducing sensitivity [8, 9, 10, 11, 12]. These structures provide multiple positions for incorporating explosophore groups such as nitro (—NO_2_), nitrate ester (—ONO_2_), nitramino (—NHNO_2_), and azido (—N_3_), which can improve heat of formation (HOF), detonation velocity (D), and pressure (*P*_CJ_) [13, 14, 15].

The five‐membered fused six‐membered nitrogen heteroaromatic ring system has recently been established as a particularly promising skeleton class for energetic materials design. Lu et al. [16] systematically characterized all 213 possible skeletons of this type using DFT calculations, providing design guidelines regarding nitrogen atom number, position, and their effects on stability and energy content. Building on this foundation, the present work investigates the substitution effects of key explosophore groups on selected scaffolds within this class, extending the analysis from bare skeletons to functionalized energetic candidates.

Compounds 5‐H‐imidazo‐[4,5‐c]‐pyridazine (I) and 1,2,4‐triazolo‐[4,3‐a]‐pyrazine (T) were selected due to their optimal combination of high nitrogen content, multiple functionalization sites, and rigid coplanar structures expected to provide enhanced formation enthalpy, superior thermal stability, and reduced sensitivity while maintaining essential density and performance characteristics. To the best of our knowledge, 5‐H‐imidazo‐[4,5‐c]‐pyridazine (I) and 1,2,4‐triazolo‐[4,3‐a]‐pyrazine (T) derivatives have remained unexplored as potential energetic materials, representing a knowledge gap in the field.

**FIGURE 1:**
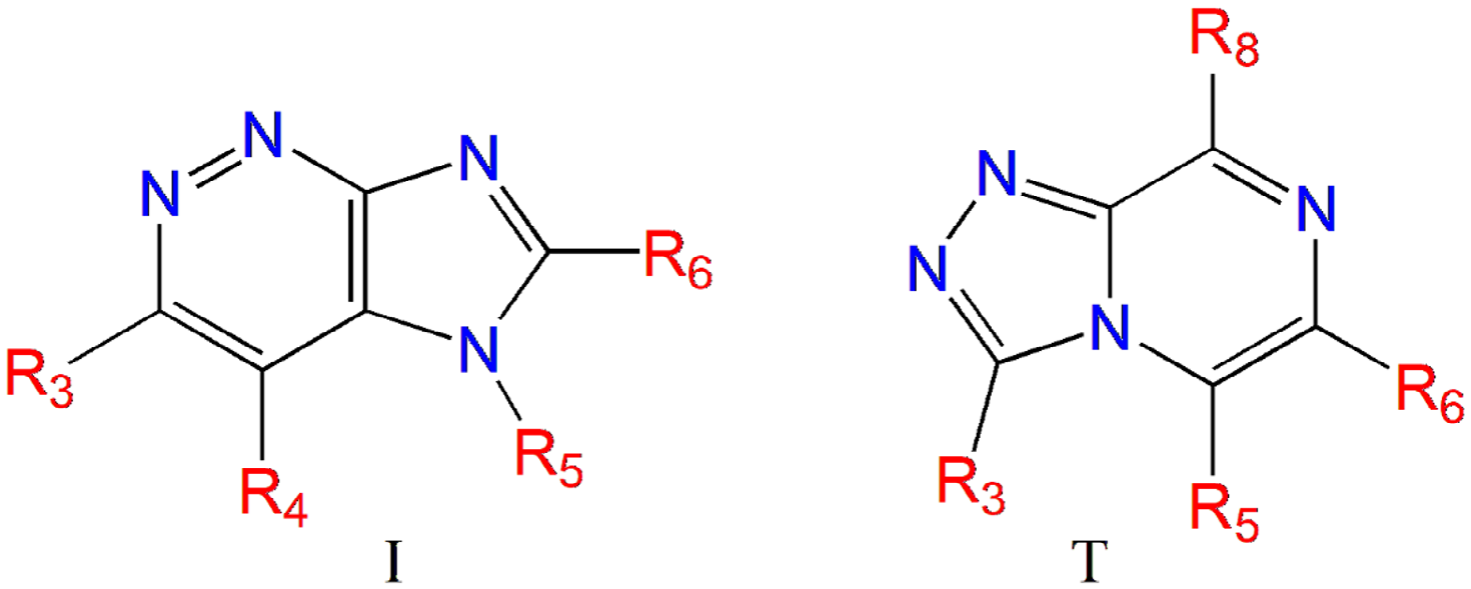
Available positions for designing novel energetic molecules based on 5‐H‐imidazo‐[4,5‐c]‐pyridazine (left) and 1,2,4‐triazolo‐[4,3‐a]‐pyrazine (right) scaffolds.

This study employed Density Functional Theory (DFT) methods to investigate 88 novel compounds based on scaffolds I and T functionalized with nitro (—NO_2_), nitrate ester (—ONO_2_), nitramino (—NHNO_2_), and azido (—N_3_) groups (see Figure 1 and Tables 1 and 2) [17]. Cartesian coordinates for all derivative compounds are provided in Table S1. Monosubstituted derivatives were excluded from the study, as they are expected to exhibit insufficient energy content for practical application as energetic materials.

**TABLE 1:** Derived energetic compounds of 5‐H‐imidazo‐[4,5‐c]‐pyridazine (I).

Position	IR34	IR35	IR36	IR45	IR46	IR56	IR345	IR346	IR356	IR456	IR3456
3	‐R	‐R	‐R	‐H	‐H	‐H	‐R	‐R	‐R	‐H	‐R
4	‐R	‐H	‐H	‐R	‐R	‐H	‐R	‐R	‐H	‐R	‐R
5	‐H	‐R	‐H	‐R	‐H	‐R	‐R	‐H	‐R	‐R	‐R
6	‐H	‐H	‐R	‐H	‐R	‐R	‐H	‐R	‐R	‐R	‐R

*Note:* The second letter R indicates the explosophore group (*A* = —NO_2_; *B* = —NHNO_2_; *C* = —N_3_ and *D* = —ONO_2_) and the number represents the positions displayed in the Figure 1.

**TABLE 2:** Derived energetic compounds of 1,2,4‐triazolo‐[4,3‐a]‐pyrazine (T).

Position	TR35	TR36	TR38	TR56	TR58	TR68	TR356	TR358	TR368	TR568	TR3568
3	‐R	‐R	‐R	‐H	‐H	‐H	‐R	‐R	‐R	‐H	‐R
5	‐R	‐H	‐H	‐R	‐R	‐H	‐R	‐R	‐H	‐R	‐R
6	‐H	‐R	‐H	‐R	‐H	‐R	‐R	‐H	‐R	‐R	‐R
8	‐H	‐H	‐R	‐H	‐R	‐R	‐H	‐R	‐R	‐R	‐R

*Note:* The second letter R indicates the explosophore group (*A* = —NO_2_; *B* = —NHNO_2_; *C* = —N_3_ and *D* = —ONO_2_) and the number represents the positions displayed in the Figure 1.

The predicted properties examined included HOMO‐LUMO energy gaps for chemical stability assessment [18], Mayer bond order (MBO) analysis for trigger bond identification [19, 20], aromaticity evaluation via the nucleus‐independent chemical shift (NICS(1)_ZZ_) index [21, 22, 23], and molecular electrostatic potential (MEP) distribution [24, 25]. Additional properties examined included heat of formation and detonation parameters for energy content evaluation, and impact sensitivity [26] for safety profiling. This computational approach enables the preliminary screening of critical parameters across structural variants, thereby minimizing synthesis hazards and resources while identifying promising candidates for experimental development. The thermal stability of these candidates was further evaluated through bond dissociation enthalpy (BDE) calculations.

## 2 Computational and Theoretical Methods

Quantum mechanical calculations were performed using Gaussian 09 (Rev. E.01) with default settings [27]. Molecular geometries were optimized at the B3LYP/6‐311G** level, with vibrational frequency analysis confirming all structures as true minima. Single‐point calculations at B3LYP/def2‐TZVPP provided frontier molecular orbital energies.

The B3LYP functional was selected for its established performance in thermochemical calculations of nitrogen‐rich energetic compounds [28, 29] and its consistency with the parametrization of the empirical models used for crystal density and heat of sublimation (Equations 6 and 7) [30, 31, 32]. Dispersion correction was not included in the geometry optimization step, as all calculations were performed on isolated gas‐phase molecules where intramolecular geometry parameters are primarily governed by short‐range covalent interactions; additionally, the empirical models of Rice and Byrd [32] and Politzer et al. [31] were parametrized without dispersion correction, and its inclusion would create an inconsistency with their parametrization domain.

To provide additional insight into the structure–property relationships of the designed compounds, an aromaticity analysis was carried out using the optimized geometries obtained at the theoretical level described above. Aromaticity was evaluated through the nucleus‐independent chemical shift (NICS) approach, a widely employed magnetic criterion defined as the negative value of the computed magnetic shielding tensor at selected points in space [21, 23]. In this work, the out‐of‐plane tensor component, NICS(1)_ZZ_, was adopted as an auxiliary descriptor due to its greater sensitivity to π‐electron delocalization and reduced contribution from *σ*‐electrons. Following the standard protocol reported in the literature, the NICS probe points were positioned 1.0Å above the geometric center of the benzene and five‐membered rings, perpendicular to the ring plane along the *z*‐axis [22]. This approach allows the assessment of local aromatic character and its correlation with the electronic and energetic properties of the investigated systems. The Multiwfn program (version 6.2) [33, 34] was used to compute the NICS(1)_ZZ_ values.

Although M06‐2X or double‐hybrid functionals could improve absolute energy accuracy, heats of formation were obtained via isodesmic reactions (Equation 1), which cancel a substantial portion of systematic errors in electron correlation by comparing structurally similar species [30]. The use of the large def2‐TZVPP basis set for single‐point energies further reduces basis set incompleteness errors, and together these strategies mitigate the known limitations of B3LYP for absolute energy calculations without requiring the computational cost of higher‐level methods across an 88‐compound dataset.

Gas‐phase heats of formation (HOF_gas_) at 298 K were calculated using isodesmic reactions (Equation 1) with experimental reference compounds (CH_4_, NH_3_, CH_3_NO_2_, NH_2_NO_2_, CH_3_ONO_2_), where R represents energetic functional groups (—NO_2_, —NHNO_2_; —N_3_ or —ONO_2_), and *n*_1_ and *n*_2_ represent the number of substituents on carbon and nitrogen ring atoms, respectively.

(1) $$C_{5}H_{\left(4-n_{1}-n_{2}\right)}N_{4}R_{\left(n_{1}+n_{2}\right)}+n_{1}CH_{4}+n_{2}NH_{3}\to C_{5}H_{4}N_{4}+n_{1}CH_{3}R+n_{2}{\mathrm{NH}}_{2}R$$

The heats of reaction were determined by Equations (2) and (3), where ΔE₀ is the total electronic energy difference at 0 K, ΔZPE is the zero‐point energy difference, and $\Delta H_{T}$ is the thermal correction from 0 to 298 K. For reactions in the gas phase, $\Delta \left(\mathrm{PV}\right)$ is equal to ΔnRT, and Δn = 0 for the designed isodesmic reactions [35]:

(2) $$\mathrm{HO}F_{\mathrm{gas}}=\sum \mathrm{HO}F_{\mathrm{gas}}\left(\text{Products}\right)-\sum \mathrm{HO}F_{\mathrm{gas}}\left(\text{Reactants}\right)$$

(3) $$\mathrm{HO}F_{\mathrm{gas}}=∆E_{298K}+∆\left(\mathrm{PV}\right)=∆E_{0}+∆\mathrm{ZPE}+∆H_{T}+∆\mathrm{nRT}$$

For comparison, literature values for the $\mathrm{HO}F_{\text{solid}}$ of two conventional explosives (trinitrotoluene, TNT, and hexogen, RDX) were also used [36, 37, 38]. For compounds lacking experimental HOF data, HOF_gas_ values were computed with the CBS‐4 M method [39, 40] using atomization reactions (Equation 4):

(4) $$C_{a}H_{b}N_{c}O_{d}\to \mathrm{aC}+\mathrm{bH}+\mathrm{cN}+\mathrm{dO}$$

Considering that energetic materials are employed in the condensed phase, solid‐state properties were calculated using Hess's law (Equation 5)

(5) $$\mathrm{HO}F_{\text{solid}}=\mathrm{HO}F_{\mathrm{gas}}-\Delta H_{\mathrm{sub}}$$

where the heat of sublimation ($\Delta H_{\mathrm{sub}}$) was determined using the empirical model proposed by Politzer et al. [41] (Equation 6).

(6) $$\Delta H_{\mathrm{sub}}=aA^{2}+b\sqrt{\left(\nu \sigma_{\mathrm{tot}}^{2}\right)}+c$$

where A is the surface area of a 0.001 contour value of electronic density on the isosurface A (in Å^2^) and $\nu \sigma_{\mathrm{tot}}^{2}$ (in kcal^2^ mol^−2^) is the electrostatic interaction index, ν represents the degree of balance between positive and negative electrostatic potentials, and $\sigma_{\mathrm{tot}}^{2}$ is a measure of electrostatic potential variability [41]. Electrostatic parameters were calculated using Multiwfn package (version 2026.6.2) [33, 34] The parameters for CHNO systems are: a = 0.000267 kcal mol^−1^ Å^−4^, b = 1.650087 and c = 2.966078 kcal mol^−1^ [30].

The theoretical crystal density (ρ) of CHNO energetic materials, was predicted using a formula proposed by Politzer et al. [31], which was improved by Rice and Byrd (Equation 7) [32]:

(7) $$\rho =\alpha \left(\frac{M}{V\left(0.001\right)}\right)+\beta \left(\nu \sigma_{\mathrm{tot}}^{2}\right)+\gamma$$

where α = 1.0462, β = 0.0021, and γ = −0.1586, M is the molar mass (in g mol^−1^) and $V\left(0.001\right)$ is the volume enclosed by the 0.001 a.u. isosurface.

Detonation velocity (D) and pressure (*P*_CJ_) were calculated using the Kamlet‐Jacobs Equations (8) and (9) [42]:

(8) $$D=1.01\left(N^{0.5}M^{0.25}Q_{\mathrm{MAX}}^{0.25}\right)\left(1+1.3\rho \right)$$

(9) $$P_{\mathrm{CJ}}=1.558\rho^{2}\left(NM^{0.5}Q_{\mathrm{MAX}}^{0.5}\right)$$

where $Q_{\mathrm{MAX}}$ is the maximum heat of detonation, in kcal kg^−1^, N is moles of gas per gram of explosive, M is grams of gas per mole of gas, D is in km s^−1^, and pressure $P_{\mathrm{CJ}}$ is in GPa, assuming combustion products of $N_{2}\left(g\right)$, $H_{2}O\left(g\right)$, $CO_{2}\left(g\right)$, and $C\left(s\right)$. Although kcal kg^−1^ is not an SI unit, it is retained here as required by the original parametrization of the Kamlet–Jacobs equations; conversion to kJ kg^−1^ would alter the empirical constants and invalidate the model.

Detonation parameters were also calculated using EXPLO5 software (version 8.01) [43], which is a thermodynamic software employing chemical equilibrium and steady‐state detonation modeling [44], and it employs the Exp‐6 equation of state based on the Buckingham exponential‐6 intermolecular potential [45]. The Exp‐6 equation of state employs an analytical representation of the excess Helmholtz free energy derived from Byers‐Brown's approach [46], where the dimensionless function $F\left(\alpha ,T^{*},\rho^{*}\right)$ is approximated by a Chebyshev polynomial in transformed canonical variables. The compressibility factor is calculated from Equations ((10), (11), (12), (13)) [45]:

(10) $$Z=\frac{\mathrm{pV}}{Nk_{B}T}=1+\rho^{*}{\left(\frac{\partial F}{\partial \rho^{*}}\right)}_{T^{*},\alpha }$$

(11) $$\rho^{*}=\frac{Nr_{m}^{3}}{V}$$

(12) $$F=\frac{A_{\mathrm{ex}}}{Nk_{B}T}$$

(13) $$T^{*}=\frac{k_{B}T}{\varepsilon }$$

where N is the number of molecules, $k_{B}$ is the Boltzmann constant, $\rho^{*}$ is the reduced density, $T^{*}$ is the reduced temperature, α is the exponential repulsion index, $r_{m}$ is the distance at the minimum potential, $A_{\mathrm{ex}}$ is the excess Helmholtz free energy and ε is the well depth. The Chapman‐Jouguet point was determined using free energy minimization, and the resulting equation system is solved using the modified Newton–Raphson method of successive approximations.

Impact sensitivity ($h_{50}$) was predicted using the following equations proposed by Pospíšil et al. [26]. For nitramines (Equation 14):

(14) $$h_{50}=-0.0064\sigma_{+}^{2}+241.42\nu -3.43$$

For other CHNO compounds (Equations 15 and 16):

(15) $$h_{50}=-234.83{\left[V_{\mathrm{eff}}-V\left(0.002\right)\right]}^{\frac{1}{3}}-3.197\nu \sigma_{\mathrm{tot}}^{2}+962$$

(16) $$V_{\mathrm{eff}}=\frac{M}{\rho }$$

where $\sigma_{+}^{2}$ is the positive electrostatic surface potential variability and $V\left(0.002\right)$ denotes the volume enclosed by 0.002 a.u. and $V_{\mathrm{eff}}$ is the effective volume.

Trigger bond identification was performed through Mayer bond order (MBO) analysis [19, 20], carried out using the Multiwfn package (version 2026.6.2) applied to the formatted checkpoint files (.fchk) generated from the B3LYP/6‐311G(d,p) geometry optimizations [33, 34]. The MBO is defined as Equation (17) [19, 20]:

(17) $$\mathrm{MBO}\left(A,B\right)=\sum {\left(P_{\alpha }S_{\alpha }+P_{\beta }S_{\beta }\right)}^{2}$$

where $P_{\alpha }$ and $P_{\beta }$ are the alpha and beta density matrices, respectively, and S is the overlap matrix. The MBO provides an orbital‐based measure of covalent bond strength and has been successfully applied for trigger bond identification in energetic materials [47, 48, 49]. For each compound, the trigger bond was identified as the covalent bond with the lowest MBO value among all C—N, N—N, C—O, and O—N bonds, applying a lower threshold of 0.30 to exclude spurious long‐range or non‐covalent interactions.

Bond dissociation enthalpies (BDEs) of the identified trigger bonds were calculated for the most promising candidates according to the standard homolytic bond cleavage scheme (Equation 18) [48]:

(18) $$A-B\left(g\right)\to A•\left(g\right)+B•\left(g\right)$$

The corresponding bond dissociation enthalpy was calculated as Equation (19)

(19) $$\mathrm{BDE}\left(A-B\right)=H_{298}\left(A•\right)+H_{298}\left(B•\right)-H_{298}\left(A-B\right)$$

where *H*_298_ is the sum of electronic and thermal enthalpies at 298 K [35]. Fragment radicals were optimized at the UB3LYP/6‐311G(d,p) level with vibrational frequency analysis confirming all species as true minima. Spin contamination was monitored through the ⟨S2⟩ expectation value; all doublet radicals yielded ⟨S2⟩ ≤ 0.756 before spin annihilation and ≤ 0.750 after spin annihilation, indicating negligible spin contamination [50]. The keywords Guess = Mix and SCF = (MaxCycle = 300, VShift = 300) were employed to assist convergence of open‐shell systems without affecting the final optimized electronic state [27].

## 3 Results and Discussion

### 3.1 HOMO‐LUMO Energy Gap

In the design of energetic materials, frontier molecular orbital energies provide critical insights into a molecule's kinetic stability and its inherent susceptibility to external stimuli. The HOMO energy (*E*_HOMO_) reflects the electron‐donating capability of the molecule, while the LUMO energy (*E*_LUMO_) indicates its electron‐accepting ability. The energy gap between them (ΔE) represents the minimum energy barrier required for an internal electronic transition from the ground state to the first excited state. Compounds with larger values exhibit lower chemical reactivity and greater kinetic stability, as electron transfer to high‐lying LUMOs or from low‐lying HOMOs is energetically unfavorable [18, 51].

Furthermore, these electronic parameters hold significant practical guiding value for predicting and modulating impact sensitivity [2, 3] The initiation of energetic materials under mechanical stimuli, such as impact or friction, is often governed by the activation of internal electronic excitations leading to bond rupture. Consequently, a wider gap generally correlates with decreased sensitivity, as a higher mechanical energy input is required to trigger the electronic transitions that precede molecular decomposition. From a molecular design perspective, carefully selecting and positioning electron‐withdrawing (EWG) or electron‐donating groups (EDG) to maximize the HOMO‐LUMO gap serves as a fundamental principle for stabilizing the energetic framework [5, 6]. This allows researchers to rationally tailor the safety profile of novel energetic candidates before experimental synthesis. The calculated values (Table 3) correlate with substituent electronic properties, following the expected trend from EDG to EWG.

**TABLE 3:** Calculated HOMO, LUMO, and HOMO‐LUMO gaps (∆E), in eV, of parent and designed compounds.

Compound	$E_{\text{HOMO}}$ (eV)	$E_{\text{LUMO}}$ (eV)	ΔE(eV)	Compound	$E_{\text{HOMO}}$ (eV)	$E_{\text{LUMO}}$ (eV)	ΔE (eV)
I	−6.48	−1.84	4.65	T	−7.07	−2.25	4.83
IA34	−8.00	−4.20	3.80	TA35	−8.37	−4.17	4.20
IA35	−7.81	−3.97	3.84	TA36	−8.48	−4.06	4.42
IA36	−7.85	−4.28	3.57	TA38	−8.38	−3.87	4.51
IA45	−7.72	−3.81	3.91	TA56	−8.32	−4.44	3.88
IA46	−7.81	−4.43	3.38	TA58	−8.36	−4.47	3.89
IA56	−7.56	−4.04	3.52	TA68	−8.34	−3.97	4.37
IA345	−8.33	−4.14	4.19	TA356	−8.73	−4.48	4.25
IA346	−8.51	−4.86	3.65	TA358	−8.84	−4.73	4.11
IA356	−8.23	−4.50	3.73	TA368	−8.95	−4.48	4.47
IA456	−7.97	−4.55	3.42	TA568	−8.78	−4.91	3.87
IA3456	−8.60	−4.91	3.69	TA3568	−9.13	−4.97	4.16
IB34	−7.51	−3.07	4.44	TB35	−7.45	−3.02	4.43
IB35	−7.49	−2.96	4.53	TB36	−7.33	−2.93	4.40
IB36	−7.31	−3.18	4.13	TB38	−7.38	−2.89	4.49
IB45	−7.31	−3.21	4.10	TB56	−7.59	−3.21	4.38
IB46	−7.09	−3.11	3.98	TB58	−7.63	−3.14	4.49
IB56	−7.12	−2.96	4.16	TB68	−7.32	−3.16	4.16
IB345	−7.84	−3.16	4.68	TB356	−7.78	−3.49	4.29
IB346	−7.64	−3.33	4.31	TB358	−7.53	−3.34	4.19
IB356	−7.68	−3.14	4.54	TB368	−7.40	−3.24	4.16
IB456	−7.48	−3.30	4.18	TB568	−7.67	−3.52	4.15
IB3456	−8.00	−3.48	4.52	TB3568	−7.78	−3.70	4.08
IC34	−6.71	−2.30	4.41	TC35	−6.71	−2.54	4.17
IC35	−6.94	−2.90	4.04	TC36	−6.60	−2.59	4.01
IC36	−6.65	−2.28	4.37	TC38	−6.69	−2.53	4.16
IC45	−6.87	−2.76	4.11	TC56	−6.54	−2.68	3.86
IC46	−6.73	−2.36	4.37	TC58	−6.67	−2.68	3.99
IC56	−6.69	−2.78	3.91	TC68	−6.68	−2.72	3.96
IC345	−6.84	−2.76	4.08	TC356	−6.33	−2.72	3.61
IC346	−6.69	−2.43	4.26	TC358	−6.65	−2.69	3.96
IC356	−6.79	−2.83	3.96	TC368	−6.56	−2.77	3.79
IC456	−6.87	−2.74	4.13	TC568	−6.41	−2.82	3.59
IC3456	−6.80	−2.74	4.06	TC3568	−6.27	−2.86	3.41
ID34	−7.37	−3.27	4.10	TD35	−7.29	−3.47	3.82
ID35	−7.35	−3.37	3.98	TD36	−7.45	−3.36	4.09
ID36	−7.23	−3.30	3.93	TD38	−7.46	−3.27	4.19
ID45	−7.21	−3.29	3.92	TD56	−7.39	−3.99	3.40
ID46	−7.11	−3.35	3.76	TD58	−7.48	−3.92	3.56
ID56	−7.02	−3.50	3.52	TD68	−7.64	−3.26	4.38
ID345	−7.62	−3.53	4.09	TD356	−7.45	−4.03	3.42
ID346	−7.51	−3.66	3.85	TD358	−7.29	−3.51	3.78
ID356	−7.46	−3.80	3.66	TD368	−7.64	−3.52	4.12
ID456	−7.35	−3.72	3.63	TD568	−7.55	−4.32	3.23
ID3456	−7.70	−3.98	3.72	TD3568	−7.58	−4.52	3.06
TNT	−8.76	−3.76	5.00	RDX	−8.80	−2.71	6.09

For reference, the ΔE values of RDX and TNT were calculated at the same theoretical level (B3LYP/def2‐TZVPP//B3LYP/6‐311G(d,p)), yielding 6.09 eV and 5.00 eV, respectively. These values indicate that the parent scaffolds I (4.65 eV) and T (4.83 eV) present lower kinetic stability than both benchmark explosives, while nitramino derivatives, which reach ΔE values up to 4.68 eV (IB345), approach the stability range of TNT.

Strong EWG like nitro (A) [52] and moderate EWGs like nitrate ester (D) [53] stabilize both frontier orbitals cooperatively through field/inductive and resonance withdrawal, with nitro showing the most pronounced effect. Although azido (C) and nitramino (B) are likewise net EWGs, confirmed by positive Hammett $\sigma_{p}$ values (a measure of the overall electronic effect on a reaction center in the para position) of 0.08 and 0.57, respectively, their frontier orbital stabilization is comparatively attenuated [54] Following the dual‐parameter decomposition of Hansch, Leo and Taft [54], in which $\sigma_{p}$ is resolved into a field/inductive component (*F*) and a resonance component (*R*), both substituents exhibit strong inductive withdrawal (*F* = 0.48 and 0.99) opposed by a π‐donor resonance contribution (*R* = −0.40 and −0.42) from lone‐pair delocalization on the α‐nitrogen. This internal F/R competition partially offsets their net withdrawing strength, unlike nitro (*F* = 0.65, *R* = 0.13) and nitrate ester (*F* = 0.48, *R* = 0.22), for which both components act cooperatively [54]. The reduced HOMO‐LUMO stabilization of groups B and C thus reflects resonance modulation of their withdrawing character, not a reversal of it.

**FIGURE 2:**
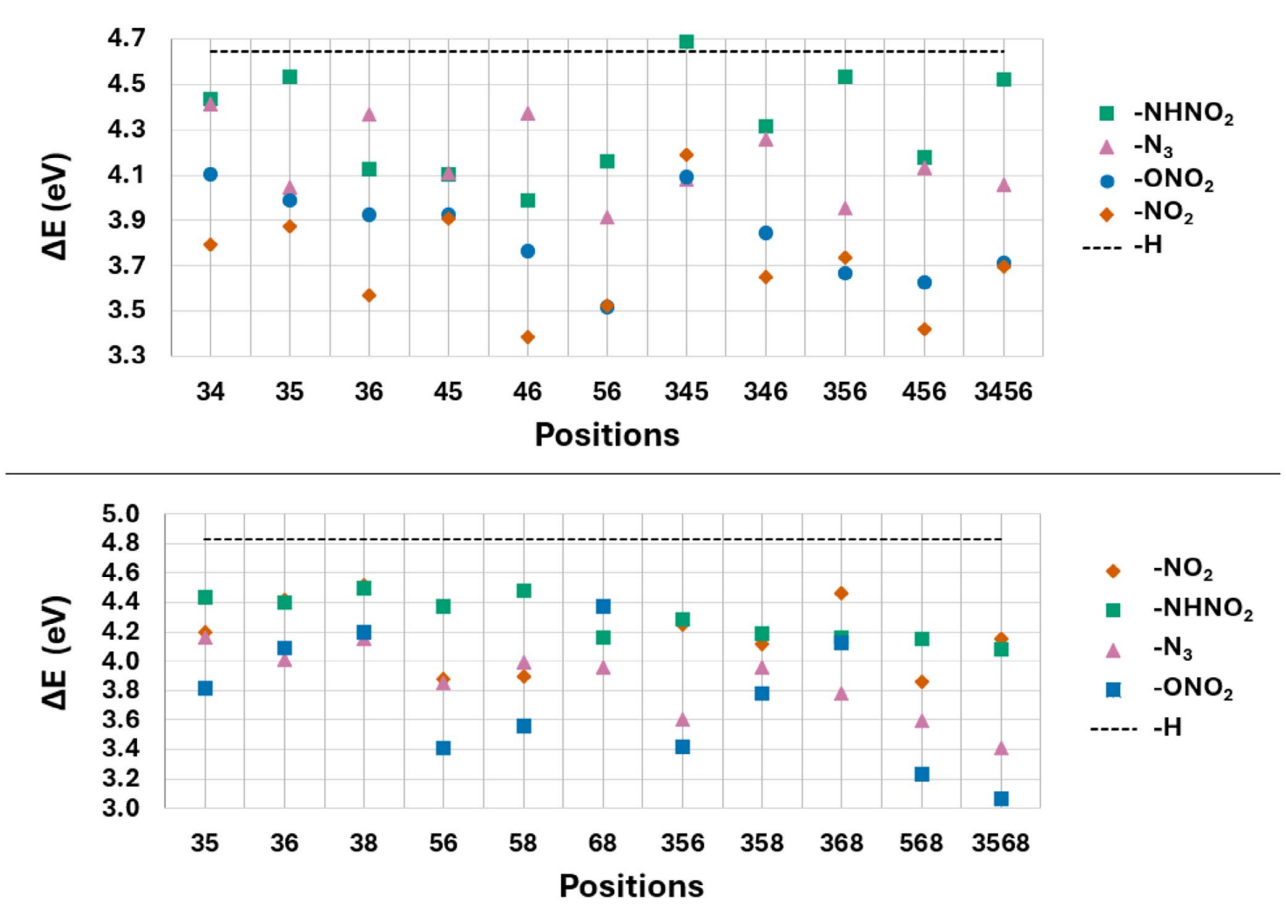
Variation trends of the HOMO‐LUMO gaps of the designed compounds as a function of the substituents' positions: 5‐H‐imidazo‐[4,5‐c]‐pyridazine (up) and 1,2,4‐triazolo‐[4,3‐a]‐pyrazine (down).

Figure 2 illustrates that ΔE trends for the derivatives depend on both substitution position and resonance effects within the ring system. When comparing similar structures, a smaller ΔE indicates lower chemical stability [51]. Within the 5‐H‐imidazo‐[4,5‐c]‐pyridazine series (I), mean ΔE values follow the order *B* > *C* > *D* > *A* (Table 3), consistent with partial resonance donation from the α‐nitrogen lone pair attenuating frontier orbital stabilization in groups *B* and *C*. Within the 1,2,4‐triazolo‐[4,3‐a]‐pyrazine series (T), this ranking shifts to *B* > *A* > *C* > *D*, reflecting the greater intrinsic electron deficiency of the triazolo‐pyrazine scaffold ($\Delta E_{T}$ = 4.83 eV vs. $\Delta E_{I}$ = 4.65 eV). This electron deficiency affects substituents asymmetrically: it amplifies HOMO stabilization by the nitro group (A), whose mean ΔE increases by 0.49 eV, while simultaneously enhancing resonance electron donation from the azido α‐nitrogen lone pair (C) into the stronger π‐acceptor ring, raising the HOMO and reducing mean ΔE by 0.29 eV. Groups B and D are largely insensitive to the scaffold change (ΔE variation of −0.03 and −0.10 eV, respectively).

It is worth noting that the stability trend observed from the HOMO‐LUMO gap analysis does not follow the classical empirical ranking for trigger bond dissociation energies (C—NO_2_ > N—NO_2_ > O—NO_2_). This distinction arises because HOMO‐LUMO gap reflects kinetic stability toward electronic reactions, whereas the classical hierarchy describes thermolytic bond rupture susceptibility under impact or thermal stimuli. In the present bicyclic framework, the —NHNO_2_ group combines the electron‐withdrawing effect of the nitramine moiety with partial electron donation through the N—H bond toward the aromatic system, effectively raising the HOMO‐LUMO separation more than the —NO_2_ group attached to carbon. This electronic interplay is specific to the fused heterocyclic scaffold and may differ from the behavior observed in benchmark molecules such as TATB, RDX, or PETN.

The HOMO‐LUMO energy gap (ΔE) analysis revealed a strong influence of substituent electronic properties and their positioning within the heterocyclic framework. Electron‐withdrawing groups (nitro and nitrate ester) stabilize frontier orbitals and lower energy ranges through electron delocalization, while electron‐donating groups (azido) increase energy ranges and destabilize structures, particularly at ring nitrogen positions. Given that nitramino may act as either EWG or EDG, these structures displayed reduced chemical reactivities.

### 3.2 Heat of Formation

The heat of formation (HOF) is a critical property of energetic materials that enables prediction of key energetic properties including detonation velocity and pressure, heat of detonation, and specific impulse. Thus, it is fundamental to accurately predict the HOF of the designed molecules. The calculated zero‐point energies (*E*_ZPE_), thermal corrections (*H*_T_), total electronic energies (*E*_0_), and $\mathrm{HO}F_{\mathrm{gas}}$ values of the reference compounds used in the isodesmic reactions are tabulated in Table 4.

**TABLE 4:** Computed total energies (*E*_0_), in a.u., ZPEs (*E*_ZPE_), thermal corrections (*H*_T_) and $\mathrm{HO}F_{\mathrm{gas}}$, all in kJ mol^−1^, of the reference compounds.

Compound	$E_{0}$ ^a^	$E_{\mathrm{ZPE}}$ ^b^	$H_{T}$ ^b^	$\mathrm{HO}F_{\mathrm{gas}}$
CH_4_	−40.5391064	115.73	10.11	−74.6^**c**^
CH_3_NO_2_	−245.1218954	128.97	14.11	‐81^c^
CH_3_NHNO_2_	−300.4823855	174.49	16.21	−73.2^d^
CH_3_N_3_	−204.17991	130.19	14.36	293.4^e^
CH_3_ONO_2_	−320.3346597	140.65	15.69	−122^c^
NH_3_	−56.5885924	89.04	10.10	−45.9^c^
NH_2_NO_2_	−261.1580388	102.28	12.26	−89.5^f^
NH_2_NHNO_2_	−316.5048218	146.20	15.51	53.7^g^
NH_2_N_3_	−220.1945754	100.63	14.15	420.8^h^
NH_2_ONO_2_	−336.3370681	109.92	15.87	44^g^
C_5_H_4_N_4_ (I)	−412.0616557	243.21	17.91	348.8^i^
C_5_H_4_N_4_ (T)	−412.0546903	242.78	17.70	372.6^i^

^a^ Calculated at the B3LYP/Def2‐TZVPP level.

^b^ Calculated at the B3LYP/6‐311G** level, and the scaling factor is 0.9887 for $E_{\mathrm{ZPE}}$ and 1.0102 for $H_{T}$ [55].

^c^ Obtained from http://webbook.nist.gov.

^d^ Obtained from reference [36].

^e^ Obtained from reference [37].

^f^ Obtained from reference [56].

^g^ Calculated data from reference [57].

^h^ Calculated data from reference [58].

^i^ Calculated at CBS‐4 M level.

Table 5 presents thermochemical parameters for the designed derivatives. Among 5‐H‐imidazo‐[4,5‐c]‐pyridazine compounds, azido derivatives exhibited the highest HOF_solid_ values, ranging from 856.3 kJ mol^−1^ (IC36) to 1677.2 kJ mol^−1^ (IC3456), attributed to the high energy content of N—N and N=N bonds. Nitro derivatives consistently showed higher values of HOF_solid_ than the parent compound (239.6 kJ mol^−1^). However, within the —NO_2_ series for the I scaffold, substitution at position 5 (a ring nitrogen) yields an N—NO_2_ linkage rather than C—NO_2_. Compounds bearing —NO_2_ at position 5 (IA35, IA45, IA56, IA345, IA356, IA456, and IA3456) are hereafter designated IA(N), while those substituted exclusively at carbon positions (IA34, IA36, IA46, and IA346) constitute IA(C). Consistently higher HOF_solid_ values for IA(N) versus IA(C) at equivalent substitution degrees, for example, IA56 (306.5 kJ mol^−1^) versus IA36 (246.2 kJ mol^−1^), and their proximity to the corresponding nitramino values, for example, IB56 (335.6 kJ mol^−1^), confirm the N—NO_2_ character of the IA(N) subgroup. Nitramino groups increased HOF_solid_ values only when added at position 5, due to the favorable energetic N—NHNO_2_ bond. Nitrate ester groups contributed positively only in disubstituted compounds at position 5.

**TABLE 5:** The total electronic energies (*E*_0_), in a.u., zero‐point energies (*E*_ZPE_), thermal corrections ($H_{T}$), gas phase heat of formation (HOF_gas_), solid phase heat of formation (HOF_gas_) and heat of sublimation ($\Delta H_{\mathrm{sub}}$), all in kJ mol‐1, are presented for the derivatives of the molecules under investigation.

Compound	$\mathrm{HO}F_{\text{solid}}$	$E_{0}$ ^a^	*E*_ZPE_ ^b^	*H*_T_ ^b^	$\mathrm{HO}F_{\mathrm{gas}}$	$\Delta H_{\mathrm{sub}}$ ^c^
IA34	263.7	−821.2128629	253.70	32.08	363.9	100.2
IA35	249.4	−821.1883165	252.30	31.80	356.3	106.9
IA36	246.2	−821.216345	253.75	31.80	354.5	108.4
IA45	288.0	−821.1767865	252.58	31.56	386.6	98.6
IA46	248.3	−821.218968	254.59	31.52	348.2	99.9
IA56	306.5	−821.1685986	251.77	32.00	407.7	101.2
IA345	313.0	−1025.742362	257.04	39.00	420.0	107.0
IA346	283.3	−1025.782305	258.47	39.21	387.2	103.9
IA356	303.7	−1025.743464	256.21	39.45	416.8	113.0
IA456	332.4	−1025.734126	255.37	39.64	440.6	108.3
IA3456	370.5	−1230.296031	259.78	47.15	483.8	113.3
TA35	341.1	−821.1881392	253.71	31.37	434.2	93.2
TA36	295.0	−821.2052783	253.70	31.49	389.4	94.4
TA38	309.1	−821.1985534	253.55	31.84	407.2	98.1
TA56	325.8	−821.1935817	252.53	31.91	419.3	93.5
TA58	307.8	−821.1990176	252.85	32.11	405.6	97.8
TA68	294.5	−821.2027593	252.66	32.06	395.5	101.0
TA356	379.3	−1025.749884	257.89	38.85	477.5	98.2
TA358	365.8	−1025.753938	258.06	38.99	467.2	101.4
TA368	322.2	−1025.770107	257.93	39.15	424.8	102.5
TA568	353.2	−1025.757987	256.63	39.74	455.9	102.6
TA3568	412.8	−1230.31209	261.91	46.61	519.7	107.0
IB34	189.1	−931.9624945	344.66	36.96	304.9	115.8
IB35	323.5	−931.9192811	341.81	37.59	445.6	122.1
IB36	163.9	−931.9700194	343.84	37.68	285.0	121.1
IB45	342.8	−931.9117901	342.78	37.07	465.7	122.9
IB46	179.3	−931.9626773	343.81	37.63	304.2	124.9
IB56	335.6	−931.9125147	341.37	37.70	463.0	127.5
IB345	328.0	−1191.857547	392.90	46.91	455.7	127.7
IB346	146.3	−1191.914559	394.09	47.30	278.1	131.9
IB356	297.8	−1191.865389	391.54	47.70	434.5	136.8
IB456	320.5	−1191.856733	392.33	47.25	457.6	137.1
IB3456	287.2	−1451.807515	442.86	56.79	434.5	147.3
TB35	263.9	−931.9384328	342.41	37.66	372.6	108.7
TB36	225.0	−931.952129	343.06	37.60	337.3	112.3
TB38	228.1	−931.950698	342.51	38.31	341.2	113.1
TB56	234.3	−931.9474735	343.14	37.06	349.0	114.7
TB58	241.0	−931.9444815	340.57	38.89	356.1	115.1
TB68	221.3	−931.949886	342.34	37.73	342.6	121.2
TB356	231.2	−1191.882306	391.79	47.42	366.8	135.6
TB358	230.1	−1191.88369	392.07	47.77	363.8	133.7
TB368	191.7	−1191.900508	391.88	48.56	320.2	128.6
TB568	204.6	−1191.891009	392.25	47.41	344.4	139.8
TB3568	209.2	−1451.826045	440.28	58.22	361.4	152.2
IC34	883.8	−739.3706175	258.71	32.41	1005.6	121.8
IC35	1029.4	−739.3191545	254.59	32.53	1147.0	117.6
IC36	856.3	−739.3801198	258.51	32.28	980.3	124.0
IC45	1056.3	−739.3107692	255.19	32.61	1169.7	113.4
IC46	876.6	−739.3732589	258.90	32.43	998.8	122.3
IC56	1034.5	−739.3186896	255.53	32.59	1149.2	114.8
IC345	1370.0	−902.9668757	262.70	39.90	1493.6	123.7
IC346	1190.6	−903.0297216	266.45	39.78	1321.9	131.4
IC356	1338.0	−902.977408	262.82	39.86	1466.0	128.1
IC456	1364.9	−902.9678771	263.11	40.04	1491.5	126.7
IC3456	1677.2	−1066.623902	270.58	47.40	1815.7	138.5
TC35	965.5	−739.3489784	257.70	32.29	1067.4	101.8
TC36	926.6	−739.3642114	258.10	32.22	1027.7	101.1
TC38	913.4	−739.3676638	258.86	32.15	1019.3	106.0
TC56	936.3	−739.3597498	257.57	32.29	1039.0	102.7
TC58	919.4	−739.3631615	257.99	32.22	1030.4	111.0
TC68	900.7	−739.3692137	257.76	32.12	1014.1	113.5
TC356	1271.3	−903.0073394	264.95	39.88	1385.4	114.1
TC358	1266.7	−903.0073692	265.44	39.61	1385.6	118.9
TC368	1226.2	−903.0227316	265.69	39.60	1345.5	119.2
TC568	1240.3	−903.0168801	265.09	39.76	1360.4	120.1
TC3568	1572.8	−1066.664294	272.42	47.39	1707.3	134.5
ID34	113.3	−971.6533063	272.19	38.17	240.8	127.5
ID35	265.8	−971.6034931	269.70	38.20	386.7	120.9
ID36	89.2	−971.6614055	271.52	38.37	219.1	129.9
ID45	283.3	−971.5963941	269.73	38.44	405.6	122.2
ID46	101.9	−971.6548974	271.36	38.73	236.3	134.4
ID56	268.6	−971.601079	269.35	38.73	393.2	124.6
ID345	224.7	−1251.390077	284.44	48.14	357.0	132.3
ID346	36.0	−1251.451046	285.69	48.84	181.3	145.3
ID356	188.2	−1251.40075	283.43	49.01	328.8	140.7
ID456	217.5	−1251.391712	283.98	48.74	352.8	135.4
ID3456	147.6	−1531.187017	298.11	58.97	300.0	152.4
TD35	159.9	−971.6440306	269.54	38.77	269.2	109.3
TD36	132.6	−971.6522234	270.70	38.31	248.4	115.8
TD38	134.4	−971.6515832	271.07	38.41	250.6	116.1
TD56	143.3	−971.6481465	269.46	38.88	258.4	115.2
TD58	139.2	−971.649567	269.99	38.77	255.1	116.0
TD68	132.2	−971.6525588	271.46	37.92	247.9	115.7
TD356	74.4	−1251.443528	283.01	49.63	205.3	130.9
TD358	65.7	−1251.445911	284.07	49.50	200.0	134.3
TD368	57.6	−1251.449269	284.76	48.78	191.1	133.6
TD568	61.7	−1251.447226	283.36	49.51	195.8	134.2
TD3568	2.7	−1531.23894	296.55	60.25	151.9	149.3

^a^ Calculated at the B3LYP/Def2‐TZVPP level.

^b^ Calculated at the B3LYP/6‐311G** level, and the scaling factor is 0.9887 for $E_{\mathrm{ZPE}}$ and 1.0102 for $H_{T}$ [55].

^c^ Calculated from molecular properties using Multiwfn package [59, 60].

For 1,2,4‐triazolo‐[4,3‐a]‐pyrazine derivatives, where all the explosophore groups are attached to ring carbon atoms, no analogous N—R ambiguity arises, and $\mathrm{HO}F_{\text{solid}}$ followed a clear trend: azido (900.7–1572.8 kJ mol^−1^) > nitro (294.5–412.8 kJ mol^−1^) > nitramino (191.7–263.9 kJ mol^−1^) > nitrate ester (2.7–159.9 kJ mol^−1^). Only azido and nitro derivatives exceeded the parent compound value (281.6 kJ mol^−1^).

Comparison with conventional explosives revealed that almost all the I and T derivatives had higher values than RDX, with only five cases (ID346, TD358, TD368, TD568, and TD3568) whose values were between TNT (−67.1 kJ mol^−1^) and RDX (66.9 kJ mol^−1^) [38]. Within each series, tetrasubstituted compounds exhibited higher $\mathrm{HO}F_{\text{solid}}$ than trisubstituted ones for azido and nitro series, while trisubstituted compounds outperformed disubstituted ones only in the azido series.

The highest $\mathrm{HO}F_{\text{solid}}$ values in nitramino and nitrate ester series corresponded to compounds with substituents at positions 45 (5‐H‐imidazo‐[4,5‐c]‐pyridazine) and 35 (1,2,4‐triazolo‐[4,3‐a]‐pyrazine), where N—NHNO_2_ and N—ONO_2_ bonds provide greater energy content than their carbon‐bonded analogs. Position 35 in 1,2,4‐triazolo‐[4,3‐a]‐pyrazine benefits from favorable electronic delocalization due to proximity of ring nitrogen atoms, enhancing resonance stabilization and increasing internal energy through effective π‐electron conjugation.

Overall, the introduction of explosophoric groups increases the energetic potential of the compounds, as evidenced by higher heat of formation (HOF) values. However, this modification simultaneously reduces the HOMO‐LUMO gap, indicating increased chemical reactivity and decreased stability. Achieving a balance between both properties is a critical aspect that must be considered in the design of new safe and efficient energetic materials.

### 3.3 Detonation Parameters

The energetic properties oxygen balance (Ω), nitrogen content $\left(\%N\right)$, density (ρ), heat of detonation ($Q_{\mathrm{MAX}}$), and the detonation velocity (D) and the detonation pressure (*P*_CJ_) are presented in Table 6. 2,6‐dinitrotoluene (DNT) and TNT were chosen as reference compounds because TNT is a traditional explosive and DNT is its precursor, making them suitable for the isodesmic approach. The computed energetic properties using B3LYP/6‐311G** showed good agreement with experimental data, with heat of formation variations below 10 kJ mol^−1^ [38], validating this theoretical level for the present work.

**TABLE 6:** Predicted densities (ρ), in g cm^−3^, maximum heat of detonation ($Q_{\mathrm{MAX}}$) in kcal kg^−1^, detonation velocities (D) in km s^−1^, and pressure (*P*_CJ_), in GPa, of designed molecules and traditional explosives.

Compound	Ω (%)	N (%)	ρ (g cm^−3^)	$Q_{\mathrm{MAX}}$ (kcal kg^−1^)	KJ		EXPLO 5	
					D (km s^−1^)	$P_{\mathrm{CJ}}$ (GPa)	D (km s^−1^)	$P_{\mathrm{CJ}}$ (GPa)
I	−160.0	46.6	1.58	656.6	4.94	10.00	5.76	11.29
T	−160.0	46.6	1.47	740.1	4.85	9.16	5.53	10.84
IA34	−53.3	40.0	1.79	1228.7	7.58	25.43	7.87	25.01
IA35	−53.3	40.0	1.81	1226.2	7.61	25.77	7.91	25.26
IA36	−53.3	40.0	1.81	1218.7	7.61	25.82	7.92	25.40
IA45	−53.3	40.0	1.78	1273.8	7.62	25.59	7.86	24.98
IA46	−53.3	40.0	1.78	1228.7	7.55	25.13	7.80	24.46
IA56	−53.3	40.0	1.78	1294.9	7.63	25.64	7.85	25.08
IA345	−28.2	38.4	1.88	1419.9	8.36	31.81	8.79	33.55
IA346	−28.2	38.4	1.86	1392.0	8.26	30.86	8.68	32.24
IA356	−28.2	38.4	1.88	1411.2	8.36	31.83	8.80	33.49
IA456	−28.2	38.4	1.88	1438.0	8.40	32.07	8.82	33.64
IA3456	−10.7	37.3	1.93	1548.0	8.88	36.37	9.35	41.70
TA35	−53.3	40.0	1.77	1334.2	7.66	25.70	7.85	25.24
TA36	−53.3	40.0	1.76	1281.7	7.56	25.01	7.77	24.33
TA38	−53.3	40.0	1.77	1297.8	7.60	25.37	7.82	24.80
TA56	−53.3	40.0	1.77	1316.8	7.63	25.55	7.83	24.97
TA68	−53.3	40.0	1.77	1281.2	7.58	25.22	7.80	24.60
TA58	−53.3	40.0	1.77	1296.3	7.61	25.41	7.82	24.87
TA356	−28.2	38.4	1.85	1482.0	8.37	31.56	8.76	33.17
TA358	−28.2	38.4	1.85	1469.3	8.35	31.40	8.74	33.01
TA368	−28.2	38.4	1.84	1428.5	8.27	30.75	8.67	32.24
TA568	−28.2	38.4	1.85	1457.5	8.33	31.24	8.72	32.85
TA3568	−10.7	37.3	1.91	1581.6	8.87	36.07	9.32	41.61
IB34	−53.3	46.6	1.79	1061.1	7.48	24.69	7.94	24.76
IB35	−53.3	46.6	1.76	1194.8	7.62	25.36	7.91	24.98
IB36	−53.3	46.6	1.78	1036.0	7.41	24.20	7.89	24.23
IB45	−53.3	46.6	1.79	1214.0	7.74	26.45	8.06	26.60
IB46	−53.3	46.6	1.80	1051.3	7.52	25.10	8.03	25.54
IB56	−53.3	46.6	1.78	1206.8	7.72	26.26	8.04	26.18
IB345	−32.0	46.6	1.81	1272.7	8.15	29.59	8.47	30.54
IB346	−32.0	46.6	1.83	1128.0	7.98	28.51	8.45	29.87
IB356	−32.0	46.6	1.81	1248.6	8.10	29.20	8.43	30.04
IB456	−32.0	46.6	1.83	1266.7	8.21	30.25	8.56	31.28
IB3456	−17.8	46.6	1.85	1294.6	8.47	32.34	8.84	34.03
TB35	−53.3	46.6	1.73	1135.5	7.44	23.93	7.73	23.32
TB36	−53.3	46.6	1.73	1096.7	7.38	23.58	7.71	23.00
TB38	−53.3	46.6	1.73	1099.9	7.38	23.6	7.71	23.02
TB56	−53.3	46.6	1.76	1106	7.47	24.39	7.84	24.09
TB58	−53.3	46.6	1.74	1112.7	7.42	23.91	7.75	23.32
TB68	−53.3	46.6	1.77	1093.1	7.49	24.64	7.89	24.57
TB356	−32	46.6	1.84	1195.6	8.11	29.54	8.53	30.91
TB358	−32	46.6	1.82	1194.7	8.05	28.88	8.43	29.85
TB368	−32	46.6	1.79	1164.1	7.90	27.56	8.27	28.29
TB568	−32	46.6	1.84	1174.4	8.09	29.44	8.54	30.79
TB3568	−17.8	46.6	1.85	1242.8	8.41	31.89	8.82	33.67
IC34	−87.1	69.3	1.71	1098.4	6.89	20.43	7.40	19.73
IC35	−87.1	69.3	1.65	1270.6	6.98	20.46	7.29	19.02
IC36	−87.1	69.3	1.70	1065.9	6.81	19.84	7.31	18.88
IC45	−87.1	69.3	1.65	1302.4	7.01	20.62	7.30	19.18
IC46	−87.1	69.3	1.69	1089.9	6.82	19.84	7.28	18.91
IC56	−87.1	69.3	1.64	1276.6	6.95	20.22	7.24	18.80
IC345	−72.1	74.9	1.69	1391.0	7.52	24.11	7.71	22.12
IC346	−72.1	74.9	1.72	1214.7	7.35	23.34	7.69	21.85
IC356	−72.1	74.9	1.68	1359.6	7.44	23.49	7.62	21.45
IC456	−72.1	74.9	1.68	1386.0	7.48	23.76	7.65	21.67
IC3456	−56.3	78.8	1.71	1410.7	7.70	25.48	7.93	23.99
TC35	−87.1	69.3	1.61	1195.1	6.76	18.94	7.02	17.51
TC36	−87.1	69.3	1.59	1149.1	6.64	18.12	6.95	17.23
TC38	−87.1	69.3	1.61	1133.4	6.67	18.41	6.95	16.89
TC56	−87.1	69.3	1.62	1160.5	6.73	18.78	7.01	17.23
TC58	−87.1	69.3	1.64	1140.5	6.75	19.06	7.08	17.66
TC68	−87.1	69.3	1.64	1118.4	6.74	19.04	7.09	17.72
TC356	−72.1	74.9	1.66	1294.1	7.28	22.38	7.46	20.31
TC358	−72.1	74.9	1.67	1289.5	7.31	22.61	7.50	20.53
TC368	−72.1	74.9	1.65	1249.7	7.20	21.81	7.38	19.68
TC568	−72.1	74.9	1.67	1263.6	7.29	22.52	7.50	20.56
TC3568	−56.3	78.8	1.7	1322.8	7.56	24.49	7.82	23.07
ID34	−33.1	34.7	1.92	1321.3	8.34	32.04	8.73	32.80
ID35	−33.1	34.7	1.83	1471.8	8.29	30.80	8.52	31.37
ID36	−33.1	34.7	1.89	1297.4	8.21	30.79	8.58	31.11
ID45	−33.1	34.7	1.85	1489.1	8.36	31.53	8.60	32.14
ID46	−33.1	34.7	1.91	1310.0	8.30	31.65	8.69	32.32
ID56	−33.1	34.7	1.84	1474.6	8.32	31.13	8.55	31.79
ID345	−7.9	32.3	1.95	1590.6	9.09	38.45	9.41	42.73
ID346	−7.9	32.3	1.99	1441.8	9.01	38.14	9.45	41.75
ID356	−7.9	32.3	1.92	1561.8	8.95	36.85	9.26	40.74
ID456	−7.9	32.3	1.93	1584.9	9.03	37.73	9.34	41.97
ID3456	8.8	30.8	2.00	1645.9	9.51	42.62	9.06	37.45
TD35	−33.1	34.7	1.81	1367.3	8.06	28.93	8.30	28.98
TD36	−33.1	34.7	1.82	1340.3	8.04	28.82	8.29	28.88
TD38	−33.1	34.7	1.82	1342.1	8.06	29.02	8.32	29.25
TD56	−33.1	34.7	1.83	1350.8	8.09	29.26	8.35	29.53
TD58	−33.1	34.7	1.82	1346.8	8.07	29.13	8.33	29.37
TD68	−33.1	34.7	1.83	1339.9	8.08	29.27	8.35	29.47
TD356	−7.9	32.3	1.9	1472	8.76	35.16	9.11	38.18
TD358	−7.9	32.3	1.91	1465.2	8.78	35.48	9.14	38.47
TD368	−7.9	32.3	1.91	1458.8	8.75	35.09	9.11	38.04
TD568	−7.9	32.3	1.91	1462	8.77	35.37	9.14	38.40
TD3568	8.8	30.8	1.97	1550.8	9.25	39.94	8.83	34.56
TNT	−74.0	18.5	1.64	1289.8	6.96	20.27	6.78	18.55
RDX	−21.6	37.8	1.77	1487.5	8.68	33.04	8.63	33.15

It is evident that the addition of groups containing oxygen atoms, such as —NO_2_, —NHNO_2_, and —ONO_2_, improves the oxygen balance, as 5‐H‐imidazo‐[4,5‐c]‐pyridazine and 1,2,4‐triazolo‐[4,3‐a]‐pyrazine are compounds with significant oxygen deficiency. The results in Table 6 show that, regarding oxygen balance, the trisubstituted compounds ID (−7.9%) and TD (−7.9%) and the tetrasubstituted IA (−10.7%), IB (−17.8%), ID (8.8%), TA (−10.7%), TB (−17.8%) and TD (8.8%) presented values superior to RDX (−21.6%).

The discrepancies between Kamlet‐Jacobs (KJ) and EXPLO 5 predictions can be attributed to fundamental differences in their computational approaches. While KJ equations rely on empirical correlations developed from traditional explosives such as TNT and RDX, EXPLO 5 employs full thermochemical equilibrium calculations of detonation products, making it more adaptable to novel molecular architectures. The bicyclic compounds investigated, particularly those with exceptionally high nitrogen content (69.3%–78.8% for azido‐rich IC/TC series), deviate significantly from the molecules sample used to parametrize KJ equations.

**FIGURE 3:**
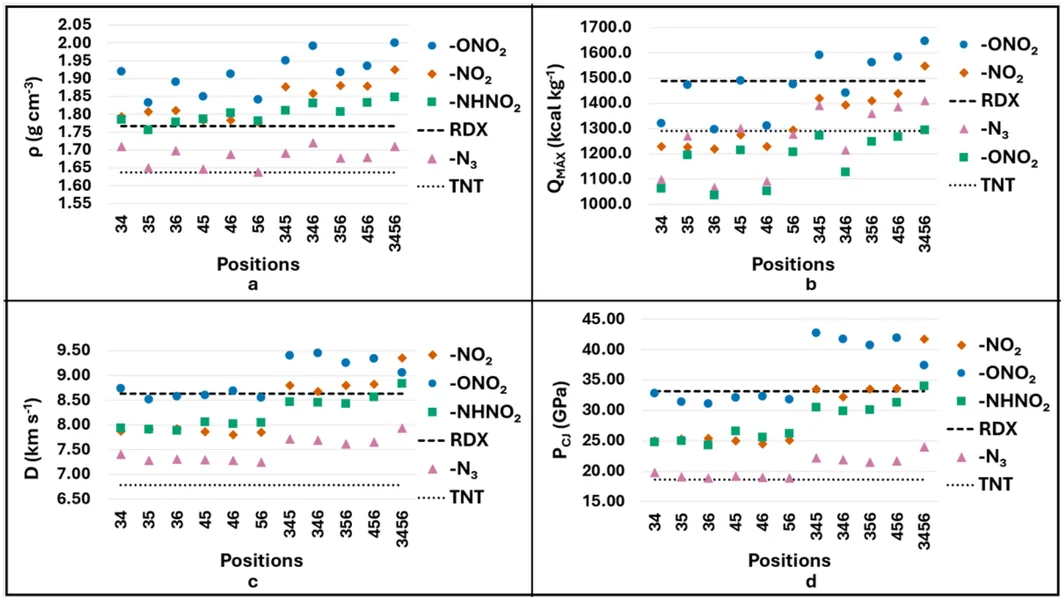
(a–d) Variation of ρ, $Q_{\mathrm{MAX}}$, D, and $P_{\mathrm{CJ}}$ for the substituted 5‐H‐imidazo[4,5‐c]‐pyridazine derivatives.

**FIGURE 4:**
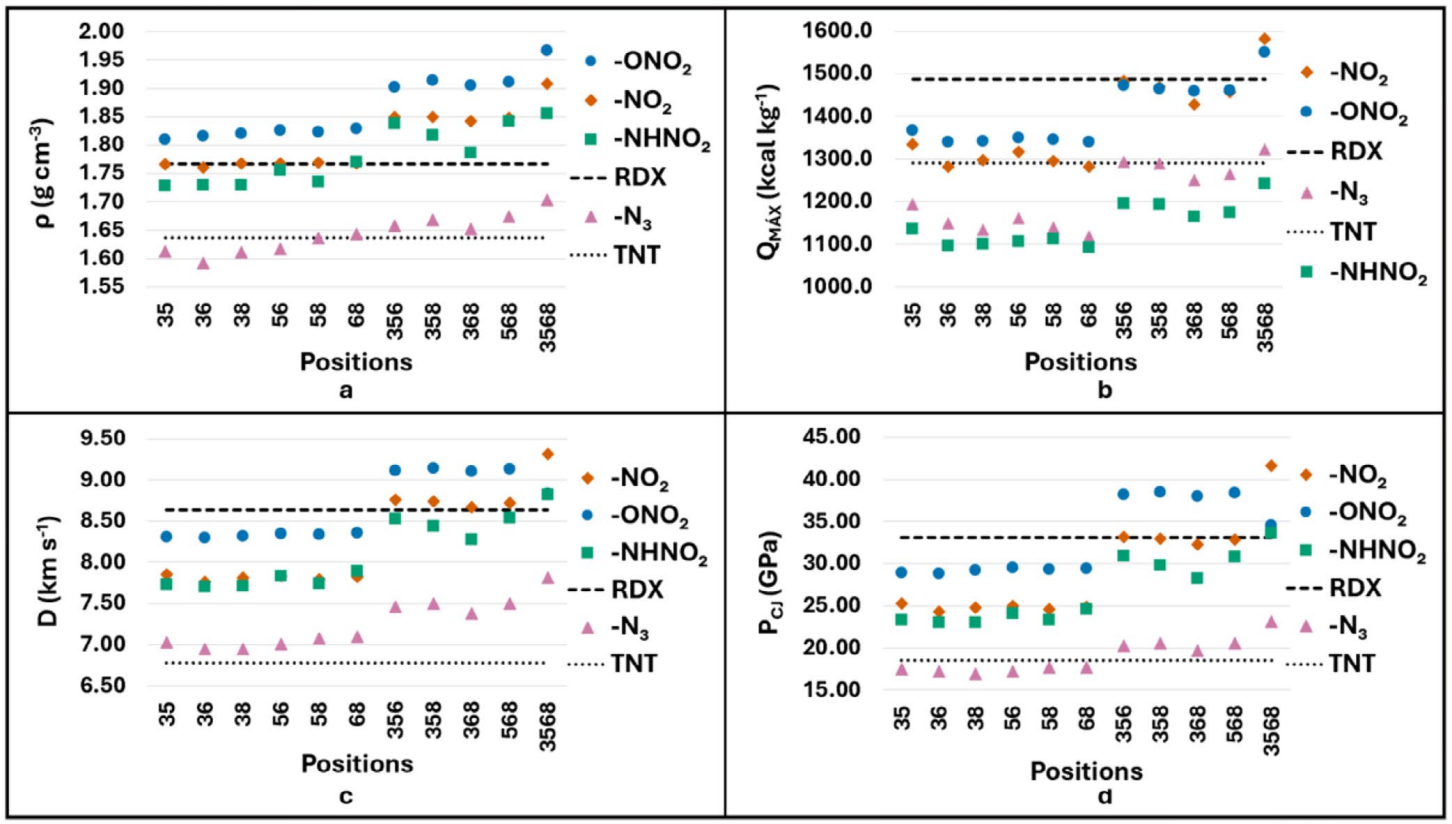
(a–d) Variation of ρ, $Q_{\mathrm{MAX}}$, D and $P_{\mathrm{CJ}}$ of substituted 1,2,4‐triazolo[4,3‐a]‐pyrazine derivatives.

Additionally, compounds with multiple azido and nitrate ester groups produce detonation product distributions that may not follow the simplified assumptions from empirical correlations. Small uncertainties in predicted densities are also amplified in detonation calculations, contributing to differences between methods. Given EXPLO 5 has demonstrated reliability for CHNO‐based energetics [61, 62, 63], Figures 3 and 4 were generated using $Q_{\mathrm{MAX}}$ from KJ equations and detonation velocity and pressure from EXPLO 5's output.

As illustrated in Figure 3a, molecules containing —ONO_2_ and —NO_2_ groups exhibited the highest density values in nearly all positions, surpassing RDX (1.77 g cm^−3^) in di‐, tri‐ and tetrasubstituted compounds. As a group, azides exhibited the lowest density values; however, many of them were observed to be higher than TNT (1.64 g cm^−3^). Applying the IA(C)/IA(N) distinction from Section 3.2, a density advantage of IA(N) over IA(C) emerges at the trisubstituted level, IA345, IA356 and IA456 (ρ = 1.88 g cm^−3^) versus IA346 (ρ = 1.86 g cm^−3^), while the nitrate ester series remains distinctly superior across all substitution degrees. The overall density contribution follows: —ONO_2_ > N—NO_2_ ≈ C—NO_2_ > —NHNO_2_ > —N_3_, with the N—NO_2_/C—NO_2_ separation becoming meaningful only at higher substitution degrees.

As demonstrated in Figure 3b, position 5, which contains a ring nitrogen atom, appears to influence $Q_{\mathrm{MAX}}$ values, suggesting that N—R bonds possess higher energy than C—R bonds. For D and $P_{\mathrm{CJ}}$ properties (Figure 3c,d), values generally increased with the number of substituents, although the tetrasubstituted nitrate ester (ID3456) deviated from this trend. All tri‐ and tetrasubstituted derivatives exceeded TNT performance (D = 6.78 km s^−1^ and $P_{\mathrm{CJ}}$ = 18.55 GPa). Consistent with the HOF trends in Section 3.2, at the trisubstituted level the IA(N) subgroup outperformed IA(C) in both D and $P_{\mathrm{CJ}}$, IA345/IA356/IA456 (D = 8.79–8.82 km s^−1^; $P_{\mathrm{CJ}}$ = 33.49–33.64 GPa) versus IA346 (D = 8.68 km s^−1^; $P_{\mathrm{CJ}}$ = 32.24 GPa), though remaining distinctly below the nitrate ester series. Notably, nitrate ester derivatives ID345, ID346, ID356, ID456 and ID3456 and the nitro‐based IA3456 demonstrated D values of 9.06–9.45 km s^−1^ and $P_{\mathrm{CJ}}$ values of 37.45–41.97 GPa, surpassing RDX benchmarks (D = 8.63 km s^−1^ and $P_{\mathrm{CJ}}$ = 33.15 GPa). Considering the IA(C)/IA(N) subdivision, the order of energetic performance for the 5‐H‐imidazo‐[4,5‐c]‐pyridazine derivatives is: —ONO_2_ > N—NO_2_ > C—NO_2_ > —NHNO_2_ > —N_3_.

The analysis of the variation trends of ρ, $Q_{\mathrm{MAX}}$, D and $P_{\mathrm{CJ}}$, presented in Figure 4, indicates that, with all groups linked to carbon atoms in the rings, changes in the position of substituents did not result in significant variations. The main differences observed are related to the number of substituents present. As expected, as the number of substituent groups increases, the values of parameters such as ρ, $Q_{\mathrm{MAX}}$, D and $P_{\mathrm{CJ}}$ for the di‐ and trisubstituted derivatives also increase. In the case of D and $P_{\mathrm{CJ}}$ properties, the tetrasubstituted nitrate ester (TD3568) also exhibited an atypical response. Since all explosophore groups in the T scaffold are attached exclusively to ring carbon atoms, the ranking holds the trend of ρ, with: —ONO_2_ > —NO_2_ > —NHNO_2_ > —N_3_. In comparison with conventional explosives, only the introduction of three nitro or nitrate ester groups or four nitro, nitramino or nitrate ester resulted in D and $P_{\mathrm{CJ}}$ values superior to those of RDX.

Considering all the energetic properties studied, 18 compounds were considered the most promising for applications as explosives, in the following order: ID346 > ID345 > IA3456 > ID456 > TA3568 > ID356 > TD358 > TD568 > TD356 > TD368 > ID3456 > IB3456 > TD3568 > TB3568 > IA456 > IA356 > IA345 > TA356. All these compounds presented D and $P_{\mathrm{CJ}}$ values superior to RDX (D = 8.63 km s^−1^ and $P_{\mathrm{CJ}}$ = 33.15 GPa).

### 3.4 Sensitivity

**FIGURE 5:**
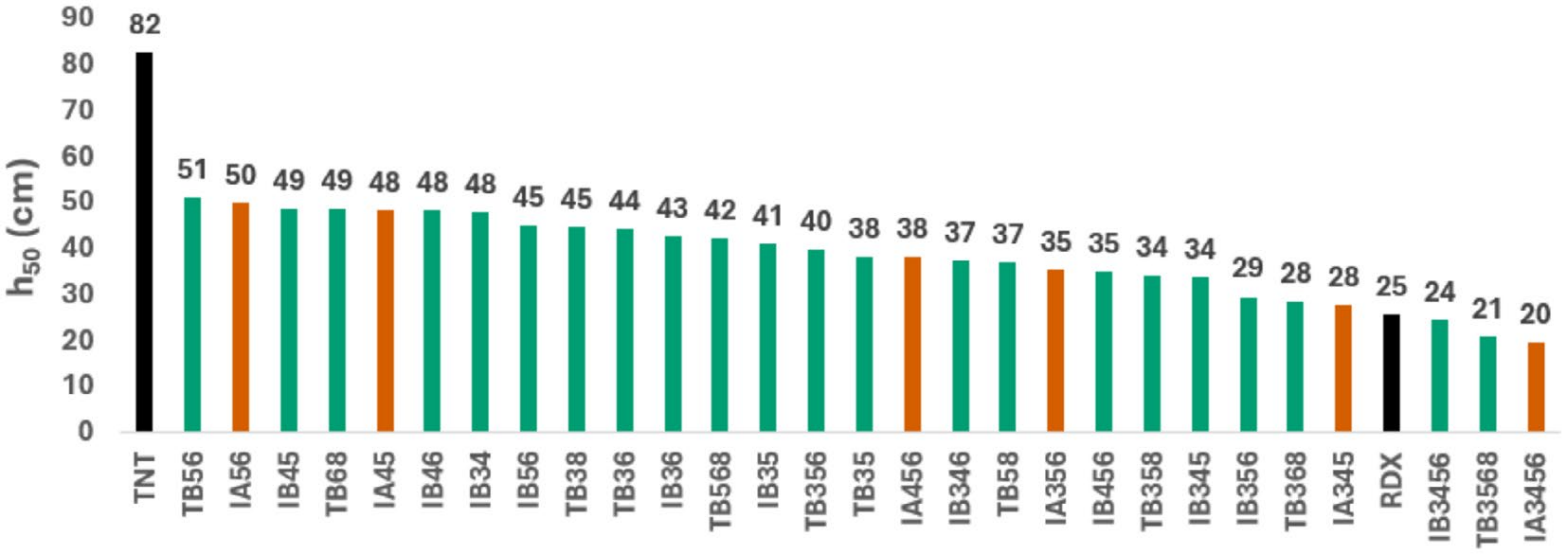
Computed $h_{50}$ values based on the nitramine model (Equation 14) compared to experimental $h_{50}$ of TNT and RDX (black bars). Green bars represent nitramino derivatives (—NHNO_2_) and vermillion bars represent nitro derivatives (—NO_2_) of the 5‐H‐imidazo‐[4,5‐c]‐pyridazine (I) and 1,2,4‐triazolo‐[4,3‐a]‐pyrazine (T) scaffolds [26].

The initial attempt to determine the impact sensitivity for the designed molecules employed the model proposed by Pospíšil et al. [26] described by Equations (14) and (15). This model is based on the free space within the compound's crystal lattice (ΔV) and the experimental values of $h_{50}$. The results obtained for the designed nitramines are illustrated in Figure 5, where it is evident that, except for compounds IB3456, TB3568, and IA3456, all remaining 26 nitramines demonstrated impact sensitivity values between those of TNT and RDX.

**FIGURE 6:**
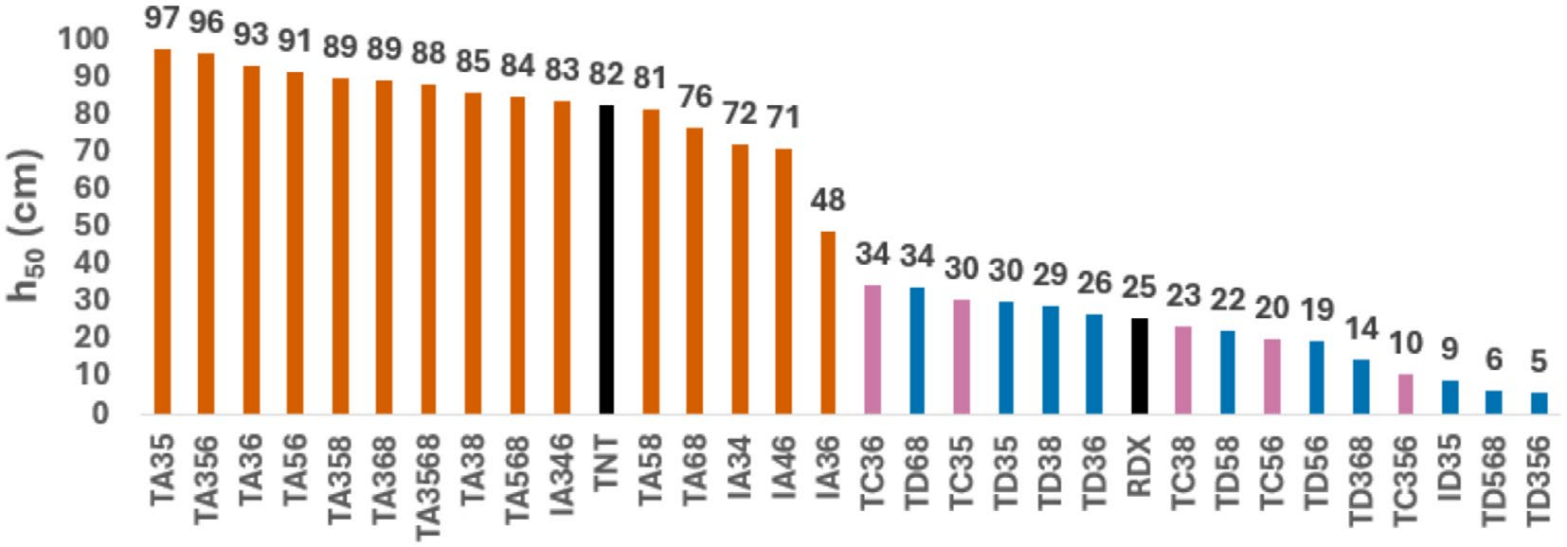
Computed $h_{50}$ values based on the non‐nitramine model (Equation 15) compared to experimental $h_{50}$ of TNT and RDX (black bars). Vermillion bars represent nitro derivatives (—NO_2_), pink‐purple bars represent azido derivatives (—N_3_), and deep blue bars represent nitrate ester derivatives (—ONO_2_) of the 5‐H‐imidazo‐[4,5‐c]‐pyridazine (I) and 1,2,4‐triazolo‐[4,3‐a]‐pyrazine (T) scaffolds [26].

The model proposed by Pospíšil et al. [26], based on Equation (15), was also applied to designed molecules that are not nitramines; however, the $h_{50}$ values of 17 azido derivatives and 12 nitrate esters derivatives had no physical meaning because they were negative. Figure 6 shows the results for the remaining derived compounds which gave positive values of $h_{50}$.

The negative $h_{50}$ values obtained for the 17 azido derivatives and 12 nitrate ester derivatives arise from extrapolation beyond the applicability domain of the Pospíšil model [26]. Because the model was parameterized on a training set of 20 compounds with no organic azides and only one nitrate ester (PETN), the free‐volume descriptor (ΔV) for these classes falls outside the range for which the linear regression was calibrated, producing outputs that are mathematically consistent but physically meaningless. Accordingly, the Pospíšil non‐nitramine model is considered appropriate only for the C‐nitro and N‐oxide derivatives within this study, for which positive and physically coherent $h_{50}$ predictions can be obtained.

Analysis of the data from Figure 6 reveals distinct patterns among the different energetic derivative classes. Nitro derivatives (TA/IA compounds) consistently demonstrate superior impact sensitivity compared to TNT and RDX. Notably, TA35, TA356, TA36, TA 56, TA358, TA368, TA3568, TA38, TA 568, and IA346 all exhibit $h_{50}$ values higher than TNT, indicating enhanced safety characteristics. The remaining nitro derivatives (TA58, TA68, IA34, IA46, and IA36) also show favorable $h_{50}$ values, between TNT and RDX. The nitrate ester derivatives (TD/ID series) consistently demonstrate the highest impact sensitivity, with $h_{50}$ values predominantly below those of RDX.

Overall, the data suggests that while many nitro derivatives offer promising alternatives to both TNT and RDX with respect to impact sensitivity, azido and particularly nitrate ester derivatives would require additional stabilization strategies to achieve comparable safety profiles.

**FIGURE 7:**
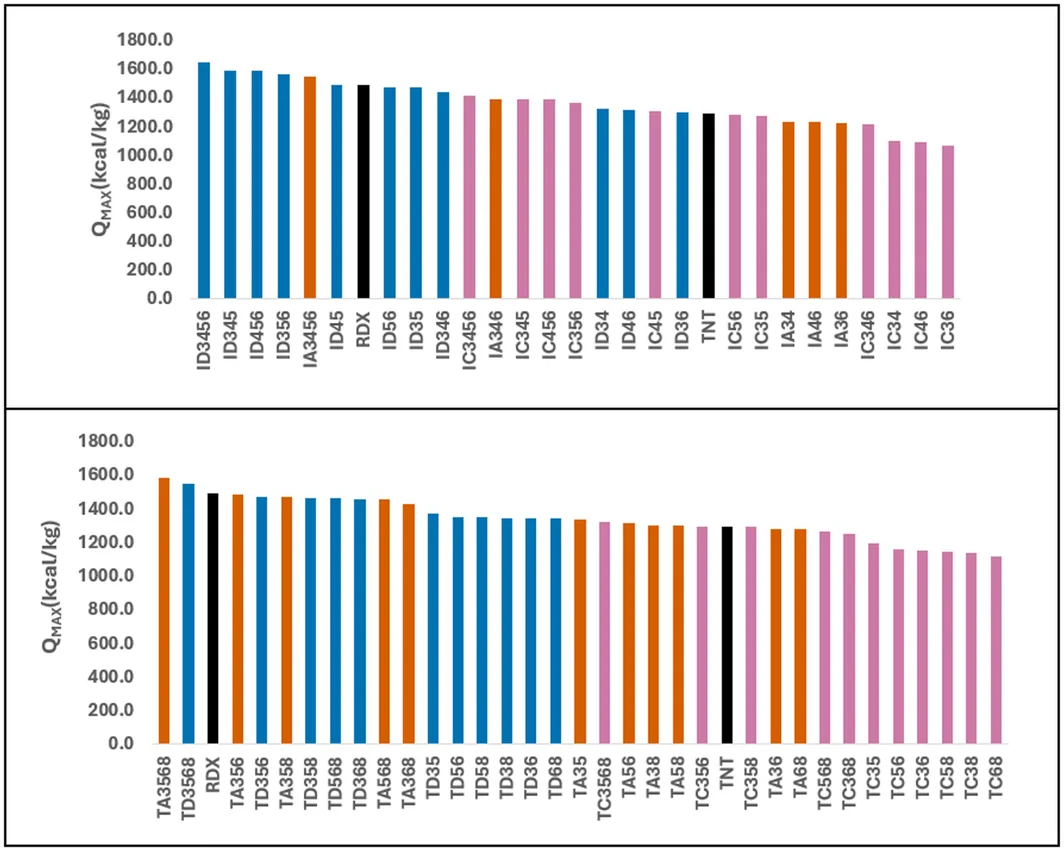
Estimated $Q_{\mathrm{MAX}}$ for the designed non‐nitramine compounds in descending order for the I (above) and T (below) scaffolds. Deep blue bars represent nitrate ester derivatives (—ONO_2_), vermillion bars represent nitro derivatives (—NO_2_), and pink‐purple bars represent azido derivatives (—N_3_). RDX and TNT reference values are represented in black.

In a subsequent study, Politzer and Murray [64] observed that, for non‐nitramine compounds, there exists a general tendency whereby higher $Q_{\mathrm{MAX}}$ results in lower $h_{50}$ values. Thus, a second attempt to predict $h_{50}$ was based on maximum detonation energy (Q_MAX_). Figure 7 presents the data collected in Table 6, from which it can be inferred that, among the non‐nitramine compounds, ID3456, ID345, ID456, ID356, ID45, TA3568, and TD3568 exhibit greater sensitivity than RDX.

Figure 7 shows that, generally, nitrate esters present higher $Q_{\mathrm{MAX}}$ values compared to azides, supporting the conclusion that nitrate esters tend to be more sensitive than azides. This observation is consistent with the research conducted by Zhang et al. [5], who investigated the effects of substituents on dipicrylamide derivatives, and finds a parallel in the data reported by Zhao and colleagues for fused tetrazole derivatives [15]. However, those authors noted that the relative ordering between these functional groups may vary depending on the molecular structure and degree of substitution, which justifies the individualized analysis conducted in the present work.

### 3.5 Aromaticity and Electrostatic Potential Analysis

**FIGURE 8:**
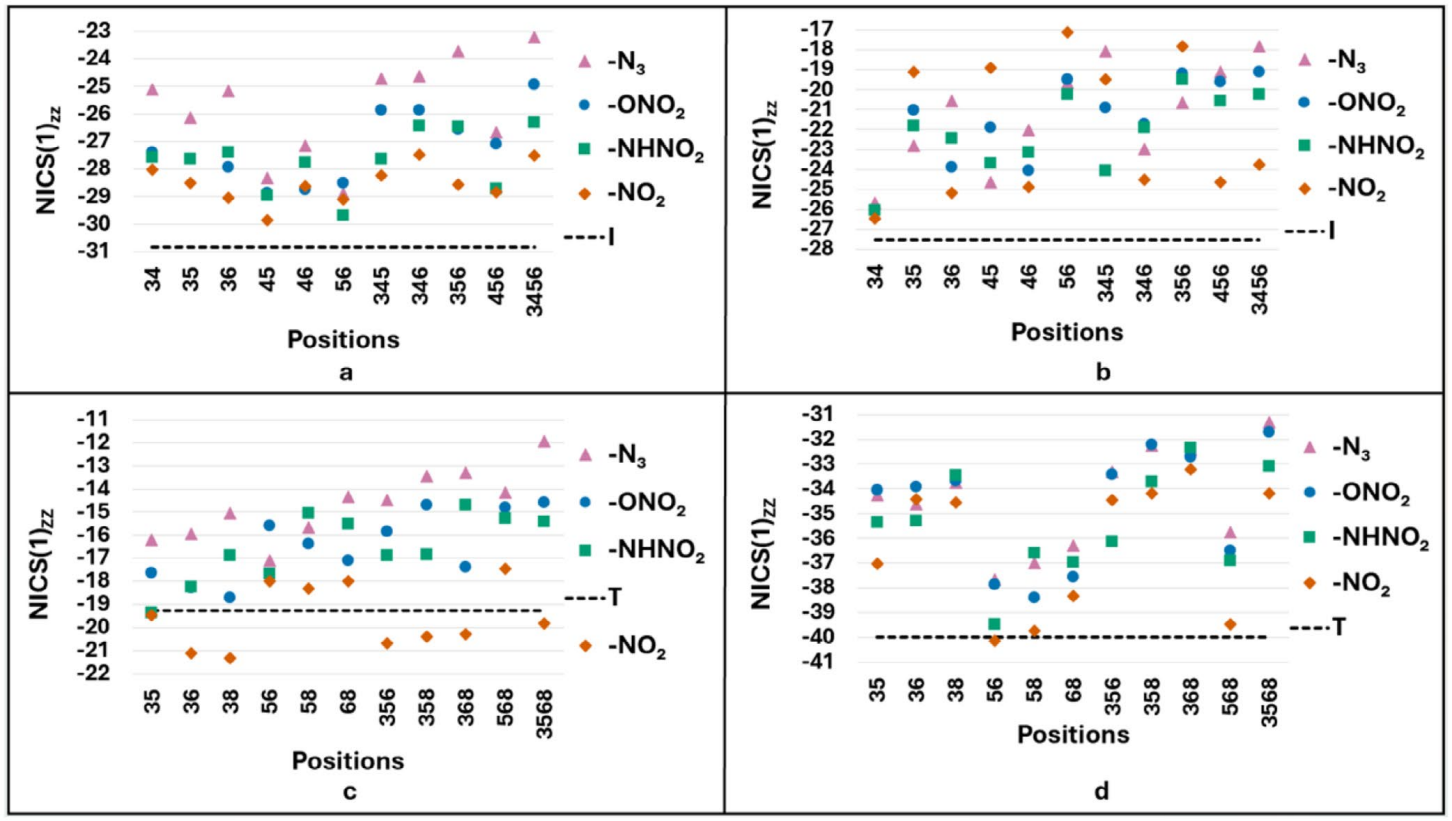
$\text{NICS}{\left(1\right)}_{\mathrm{zz}}$ for the investigated systems: (a) 6‐member ring and (b) 5‐member ring for the 5‐H‐imidazo[4,5‐c]‐pyridazine derivatives; (c) 6‐member ring and (d) 5‐member ring for the 1,2,4‐triazolo[4,3‐a]‐pyrazine derivatives. The dashed black lines represent the value of the unsubstituted systems.

Aromaticity analysis using the $\text{NICS}{\left(1\right)}_{\mathrm{zz}}$ index (Figure 8) revealed that the introduction of different explosophore groups causes distinct perturbations in the electronic distribution of the systems studied. For the 5‐H‐imidazo[4,5‐c]‐pyridazine derivatives, the —N_3_ substituent was observed to cause a significant reduction in aromaticity within the six‐membered ring, evidenced by an increase in $\text{NICS}{\left(1\right)}_{\mathrm{zz}}$ values (making them less negative). This behavior suggests reduced π‐delocalization efficiency, resulting in lower stability of the heteroaromatic core.

In the five‐membered ring, the —NO_2_ and —ONO_2_ substituents, when introduced at the 5‐position, yielded the highest $\text{NICS}{\left(1\right)}_{\mathrm{zz}}$ values, indicating a more pronounced reduction in aromatic character in that region. This result highlights the particular significance of functionalization at this position, as it compromises the stability of the unsubstituted core. Overall, all 5‐H‐imidazo[4,5‐c]‐pyridazine derivatives exhibited less negative $\text{NICS}{\left(1\right)}_{\mathrm{zz}}$ values compared to the reference compound, indicating that substitution systematically reduces aromatic character and, consequently, the electronic stability of the system.

For the 1,2,4‐triazolo[4,3‐a]‐pyrazine derivatives, the behavior proved more complex. Although the —N_3_ substituent also significantly reduced the aromaticity of the six‐membered ring, some exceptions were observed. Specifically, compounds substituted with —NO_2_ at positions 36, 38, 356, 358, 368, and 3568 displayed more negative $\text{NICS}{\left(1\right)}_{\mathrm{zz}}$ values than the unsubstituted core, indicating an increase in aromaticity. This behavior may be related to the resonance‐based electron‐donating capacity of the —NO_2_ group, resulting in a more balanced electron density within the ring. In contrast, the effects of substituents on the five‐membered ring were less pronounced for this series, suggesting lower electronic sensitivity of this region to functionalization. Nevertheless, the derivatives containing —ONO_2_ generally exhibited the highest $\text{NICS}{\left(1\right)}_{\mathrm{zz}}$ values, indicating that this group is the one that most compromises aromatic character.

The correlation between $\text{NICS}{\left(1\right)}_{\mathrm{zz}}$ values and detonation‐related properties reveals a consistent structure–property relationship: less negative $\text{NICS}{\left(1\right)}_{\mathrm{zz}}$ values, indicating reduced aromatic character and π‐electron delocalization, are associated with higher density (ρ), heat of detonation ($Q_{\mathrm{MAX}}$), detonation velocity (D), and detonation pressure (*P*_CJ_), suggesting that the partial loss of aromatic stabilization increases the detonation energy yield. Conversely, greater aromatic character is linked to enhanced molecular stability at the expense of energetic performance. The results are presented in Figures S1–S4.

Molecular electrostatic potential (MEP) maps (Tables S2 and S3) complement the $\text{NICS}{\left(1\right)}_{\mathrm{zz}}$ results, showing that explosophore substitution redistributes electron density across the molecular surface. The —NO_2_, —NHNO_2_, —N_3_, and —ONO_2_ groups act as electron‐withdrawing substituents, modifying the electronic structure of the conjugated heteroaromatic systems through combined inductive and resonance effects and affecting the π‐electron distribution and aromatic character of the fused rings. Specifically, the MEP maps show the electrostatic potential at the ring centers shifting from nearly neutral or slightly negative (green, in the unsubstituted core) to more positive (blue, in the substituted derivatives), reflecting the electron‐withdrawing inductive effect of the explosophore groups. This depletion of electron density reduces availability for π‐delocalization, weakening the diatropic ring current and yielding less negative NICS(1)_ZZ_ values, consistent with reduced aromatic character [24, 25].

This accumulation of positive density at the centers of the 5‐ and 6‐membered rings, associated with reduced π‐electron delocalization and less negative NICS(1)_ZZ_ values, indicates decreased electronic stability. This trend aligns with the calculated detonation properties: compounds exhibiting greater MEP polarization and reduced aromatic character generally display higher values for density, heat of detonation, detonation velocity and pressure. Together, these results show that combining MEP and NICS analyses gives a consistent interpretation of the relationship between electronic structure, stability, and energetic performance in the compounds studied.

### 3.6 Trigger Bond Identification and Thermal Stability

The thermal stability of energetic compounds is closely related to the strength of the covalent bond that initiates the decomposition process, commonly referred to as the trigger bond [65]. The rupture of this specific linkage governs the initial reaction kinetics that lead to detonation, meaning that weaker trigger bonds generally increase the sensitivity of the explosive to external stimuli. Therefore, identifying the weakest bond in a molecule is essential for understanding its intrinsic stability and safety profile. Accordingly, the Mayer bond order (MBO) which quantifies the degree of electronic sharing between two bonded atoms [19, 20], has been used as an efficient descriptor to identify potentially weak bonds in energetic materials [66, 67, 68, 69], as its values correlate positively with bond strength. Consequently, lower bond order values indicate weaker covalent interactions and a greater propensity for homolytic cleavage.

Compared with bond dissociation energy (BDE) calculations, which require additional calculations for the radical fragments formed upon bond cleavage, MBO is obtained directly from the electronic structure of the optimized molecule, making it an efficient descriptor for screening large molecular libraries [66]. Accordingly, MBO values were calculated for all 88 designed compounds, whereas BDE calculations were performed only for the most promising candidates selected from the analyses in Sections 3.3 and 3.4. The MBO values of the trigger bonds are summarized in Table 7 and fully detailed in Table S4.

**TABLE 7:** Trigger bond type and Mayer bond order (MBO) range for the 88 designed compounds, grouped by scaffold, explosophore class, trigger bond type, and substitution position. The detailed data for all compounds are provided in Table S4.

Compounds	Nr of compounds	Explosophore	Trigger bond	Position	MBO (minimum)
IA36, IA46, IA56, IA346	4	—NO_2_	C—NO_2_	R6	0.737–0.748
IA35, IA345, IA356	3	—NO_2_	C—NO_2_	R3	0.738–0.747
IA34, IA45	2	—NO_2_	C—NO_2_	R4	0.773–0.789
IA456, IA3456	2	—NO_2_	N—NO_2_	R5	0.606–0.620
IB35, IB45, IB56, IB345, IB356, IB456, IB3456	7	—NHNO_2_	HN—NO_2_	R5	0.784–0.818
IB34, IB46, IB346	3	—NHNO_2_	C—NHNO_2_	R4	0.917–0.935
IB36	1	—NHNO_2_	C—NHNO_2_	R3	0.944
IC35, IC45, IC56, IC345, IC356, IC456, IC3456	7	—N_3_	N=N_3_	R5	0.939–0.964
IC34, IC36, IC346	3	—N_3_	C—N_3_	R3	1.018–1.034
IC46	1	—N_3_	C—N_3_	R4	1.102
ID35, ID45, ID56, ID345, ID356, ID456, ID3456	7	—ONO_2_	O—N	R5	0.560–0.575
ID36, ID46, ID346	3	—ONO_2_	O—N	R6	0.641–0.654
ID34	1	—ONO_2_	O—N	R4	0.710
TA38, TA58, TA68, TA358, TA368, TA568	6	—NO_2_	C—NO_2_	R8	0.747–0.762
TA36, TA56, TA356, TA3568	4	—NO_2_	C—NO_2_	R6	0.746–0.782
TA35	1	—NO_2_	C—NO_2_	R3	0.774
TB35, TB36, TB38, TB358, TB368, TB3568	6	—NHNO_2_	HN—NO_2_	R3	0.823–0.882
TB56, TB58, TB356	3	—NHNO_2_	HN—NO_2_	R5	0.857–0.892
TB68, TB568	2	—NHNO_2_	HN—NO_2_	R8	0.872–0.880
TC36, TC56, TC68, TC356, TC368, TC568, TC3568	7	—N_3_	C—N_3_	R6	1.027–1.055
TC35, TC58, TC358	3	—N_3_	C—N_3_	R5	1.037–1.079
TC38	1	—N_3_	C—N_3_	R3	1.069
TD56, TD58, TD356, TD358, TD568, TD3568	6	—ONO_2_	O—N	R5	0.490–0.570
TD35, TD36, TD38, TD368	4	—ONO_2_	O—N	R3	0.568–0.585
TD68	1	—ONO_2_	O—N	R8	0.728

For nitro derivatives (IA/TA series), the trigger bond is the C—NO_2_ linkage, except for IA456 and IA3456, where —NO_2_ at the ring nitrogen position R5 forms an N—NO_2_ bond with considerably lower MBO values (0.606–0.620), reflecting the intrinsically weaker character of N—N bonds relative to C—N bonds in conjugated heterocyclic systems [66], consistent with the higher heats of formation of the IA(N) subgroup discussed in Section 3.2. Within the IA series, no single substitution site consistently acts as the trigger. Although the local electronic environment suggests a general lability trend of R4 > R3 > R6, cooperative electronic effects arising from multiple simultaneous substitutions modify this ordering in tri‐ and tetrasubstituted derivatives, preventing the establishment of a simple positional rule. In contrast, the TA series exhibits a clearer positional hierarchy (R8 > R6 > R3 > R5). Position R3, flanked by two triazole nitrogen atoms, consistently forms the strongest C—NO_2_ bond, whereas C(R6) and C(R8), adjacent to fewer ring nitrogen atoms, display progressively lower bond orders [70]. Position R5 never acts as the trigger site. A cooperative substitution effect is observed in TA3568, where full substitution shifts the trigger from R8 to R6.

For nitramino derivatives, the trigger bond depends on both scaffold and substitution pattern. In the IB series, when —NHNO_2_ occupies the ring nitrogen at R5, conjugation between the endocyclic nitrogen lone pair and the substituent strengthens the N—NHNO_2_ anchor bond, making the internal HN—NO_2_ bond the trigger [71]. When R5 is unsubstituted, the trigger shifts to the C—NHNO_2_ anchor bond, following the positional preference R4 > R3. This trend reflects differences in the local electronic environment of the ring carbons, with C(R4) forming the weakest anchor bond and C(R6), adjacent to two ring nitrogen atoms, the strongest [72, 73]. Consequently, R6 is never identified as the trigger site in the IB series. In the TB series, the internal HN—NO_2_ bond is consistently the trigger regardless of substitution position. Compared with the imidazo[4,5‐c]pyridazine scaffold, the larger number of ring nitrogen atoms in the triazolo[4,3‐a]pyrazine framework enhances the electron‐withdrawing character of the ring, strengthening the C—NHNO_2_ anchor bonds and shifting the weakest linkage to the internal HN—NO_2_ bond [72, 73]. Notably, R6 does not act as a trigger site in either scaffold for nitramino derivatives.

For azido derivatives, the nature of the trigger bond differs between the two scaffolds. In the IC series, whenever the azido group occupies R5, the N—N_3_ bond becomes the trigger, consistent with the lower stability of N—Nbonds in this electronic environment [65]. Otherwise, the trigger is the C—N_3_ anchor bond, with the positional preference R3 > R4. In the TC series, the trigger is always C—N_3_, following the hierarchy R6 > R5 > R3 > R8. Position R6, located in the pyrazine ring and surrounded by two carbon atoms, presents the lowest electron deficiency and therefore the weakest C—N_3_ bond, whereas R8 forms the strongest anchor bond. A cooperative effect is observed in TC356, where simultaneous substitution at R5 and R6 locally inverts the expected R5 > R3 order while preserving R6 as the trigger site.

It should be noted that the C—N_3_ anchor bond presents the highest MBO values in the series (1.018–1.102), which reflects its ground‐state bond strength but does not describe the actual thermal decomposition mechanism of organic azides. Analysis of the internal bond orders of the —N_3_ group confirms that C—N_1_ (MBO ≈ 1.02–1.10) and N—N (MBO ≈ 0.94–0.96) are weaker than N1—N2 (MBO ≈ 1.23–1.39) and N2—N3 (MBO ≈ 2.21–2.26). Despite its higher MBO, the N1—N2 bond is the preferential cleavage site, as thermal decomposition proceeds via stepwise N_2_ extrusion with nitrene formation as the rate‐determining step, driven by the exceptional thermodynamic stability of the N_2_ product rather than by ground‐state bond strength [74, 75]. Consequently, the C—N_3_ and N—N_3_ MBO absolute values reported here characterize anchor bond lability under homolytic conditions and are not applicable to the prediction of thermal decomposition pathways for azido derivatives, as previously identified in the literature [76, 77, 78].

For nitrate ester derivatives (ID/TD series), the internal O—N bond is consistently identified as the trigger, in agreement with the well‐established homolytic decomposition pathway of organic nitrate esters [79]. In the ID series, the weakest O—N bond occurs at R5, where the nitrate ester is attached to the ring nitrogen, followed by R6 and R4. Unlike the ID series, where R5 corresponds to a ring nitrogen, all substitutable positions in the TD series are ring carbons. The preferential lability at R5 is consistent with its lower electron deficiency compared with R3 and R8. The larger number of adjacent ring nitrogen atoms at the latter positions increases the electron‐withdrawing character of the ring, strengthening the corresponding C—ONO_2_ bonds.

The calculated MBO values span distinct ranges for each trigger‐bond type: O—NO_2_ (0.490–0.728), N—NO_2_ (0.606–0.620), C—NO_2_ (0.737–0.789), HN—NO_2_ (0.784–0.892), C—NHNO_2_ (0.917–0.944), N—N_3_ (0.939–0.964), and C—N_3_ (1.018–1.102). These ranges provide a quantitative description of the intrinsic bond strength associated with each explosophore within the investigated fused nitrogen‐rich heterocyclic frameworks.

The C—NO_2_ bond orders agree well with values reported for nitro‐substituted energetic heterocycles (typically 0.72–0.85) [47], supporting the reliability of the present calculations. Likewise, the O—NO_2_ bond orders fall within a low range, which was expected for organic nitrate esters [80], where the O—N bond is widely recognized as the trigger linkage [65]. In contrast, the N—NO_2_ bond orders are substantially lower than those commonly reported for conventional nitramines (typically 0.80–0.95) [47], which is consistent with the distinct electronic environment of N‐nitro fused aza‐heterocycles.

Although no fused aza‐heterocyclic azido derivatives directly comparable to the present system have been reported, the same limitation has been observed in previous WBI‐based studies of organic azides, in which the C—N_3_ and N—N_3_ bonds display slightly lower absolute WBI values, incorrectly suggesting these as the weaker (trigger) bonds, while N1—N2 exhibits comparatively higher values despite being the actual cleavage site [76, 77, 78], confirming that this bond‐order parameter, in absolute terms, does not correctly identify the true trigger bond in azido‐substituted systems.

Because the trigger bonds correspond to chemically distinct linkages, direct comparison of their absolute MBO values should be interpreted with caution. Instead, the calculated bond orders provide a consistent criterion for identifying the weakest bond within each molecule and for rationalizing the influence of scaffold and substitution pattern on trigger‐bond strength.

### 3.7 Identification of Promising Candidates

As previously stated, the best detonation properties among the designed molecules were found for 18 compounds: ID346, ID345, IA3456, ID456, TA3568, ID356, TD358, TD568, TD356, TD368, ID3456, IB3456, TD3568, TB3568, IA456, IA356, IA345, and TA356.

The final selection of promising candidates was carried out through a two‐step screening procedure based on sensitivity thresholds relative to RDX. For nitramines, the h_50_ values predicted directly by Pospíšil's nitramine model (Equation 14) are considered reliable; therefore, any nitramine with h_50_ lower than that of RDX was excluded. For non‐nitramine compounds, since Pospíšil's model excluded organic azides entirely and included only PETN as a nitrate ester representative, the Figure 6 criteria alone were deemed insufficient for exclusion. Instead, the secondary criterion of Politzer and Murray [64]: given that higher $Q_{\mathrm{MAX}}$ is associated with lower h_50_ among non‐nitramine compounds, any derivative with $Q_{\mathrm{MAX}}$ exceeding that of RDX (1487.5 kcal kg^−1^) was considered to present unacceptable sensitivity and was accordingly excluded. After applying both filters, nine molecules remained as promising candidates: ID346, TD358, TD568, TD356, TD368, IA456, IA356, IA345, and TA356.

The thermal stability of the nine promising candidates was further assessed through MBO analysis and BDE calculations. Among the promising candidates, the nitrate ester derivatives (ID346, TD358, TD568, TD356, TD368) present the lowest MBO values (0.490–0.641) alongside IA456 (minimum MBO = 0.620), while the remaining nitro derivatives (IA345, IA356, and TA356) present higher values (0.738–0.753).

BDE values were calculated for the four nitro‐substituted candidates and are presented in Table 8. The acceptance criterion for thermal stability requires BDE > 80 kJ mol^−1^ [81]. IA456, bearing a N—NO_2_ trigger bond with the lowest MBO among the nitro candidates (0.620), presents a BDE of 68.9 kJ mol^−1^, falling below this threshold. This confirms the greater lability of N—NO_2_ relative to C—NO_2_ linkages, consistent with the charge‐shift bond framework proposed by Joy et al. [65]. The remaining three C—NO_2_ candidates (IA345, IA356, and TA356) present BDE values ranging from 207.6 to 234.3 kJ mol^−1^, well above the stability threshold. These values are notably lower than those reported for C—NO_2_ bonds in analogous monocyclic N‐heteroaromatic systems (258–354 kJ mol^−1^) [70], consistent with the progressive reduction in C—NO_2_ BDE with increasing number of ring nitrogen atoms and degree of substitution [70].

**TABLE 8:** Trigger bond type, position, MBO, and BDE for the nitro‐substituted promising candidates.

Compounds	Trigger bond	Position	MBO (minimum)	BDE (kJ mol^−1^)
IA345	C—NO_2_	R3	0.747	211.5
IA356	C—NO_2_	R3	0.738	234.3
IA456	N—NO_2_	R5	0.620	68.9
TA356	C—NO_2_	R6	0.753	207.6

For the nitrate ester candidates, geometry optimization of the alkoxy radical fragment generated by O—N homolysis converged spontaneously to a carbonyl‐containing structure (C=O, *d* ≈ 1.19–1.20 Å) in all five cases, indicating that the radical undergoes barrierless rearrangement rather than existing as a stable species. This behavior is consistent with the known instability of α‐nitroxy alkyl radicals, which spontaneously eliminate NO₂ to form carbonyl products [82]. The calculated enthalpies therefore do not represent simple homolytic bond dissociation and cannot be directly compared to the BDE values of the nitro candidates. For these compounds, the MBO of the O—N trigger bond (0.490–0.641) serves as the thermal lability descriptor.

**FIGURE 9:**
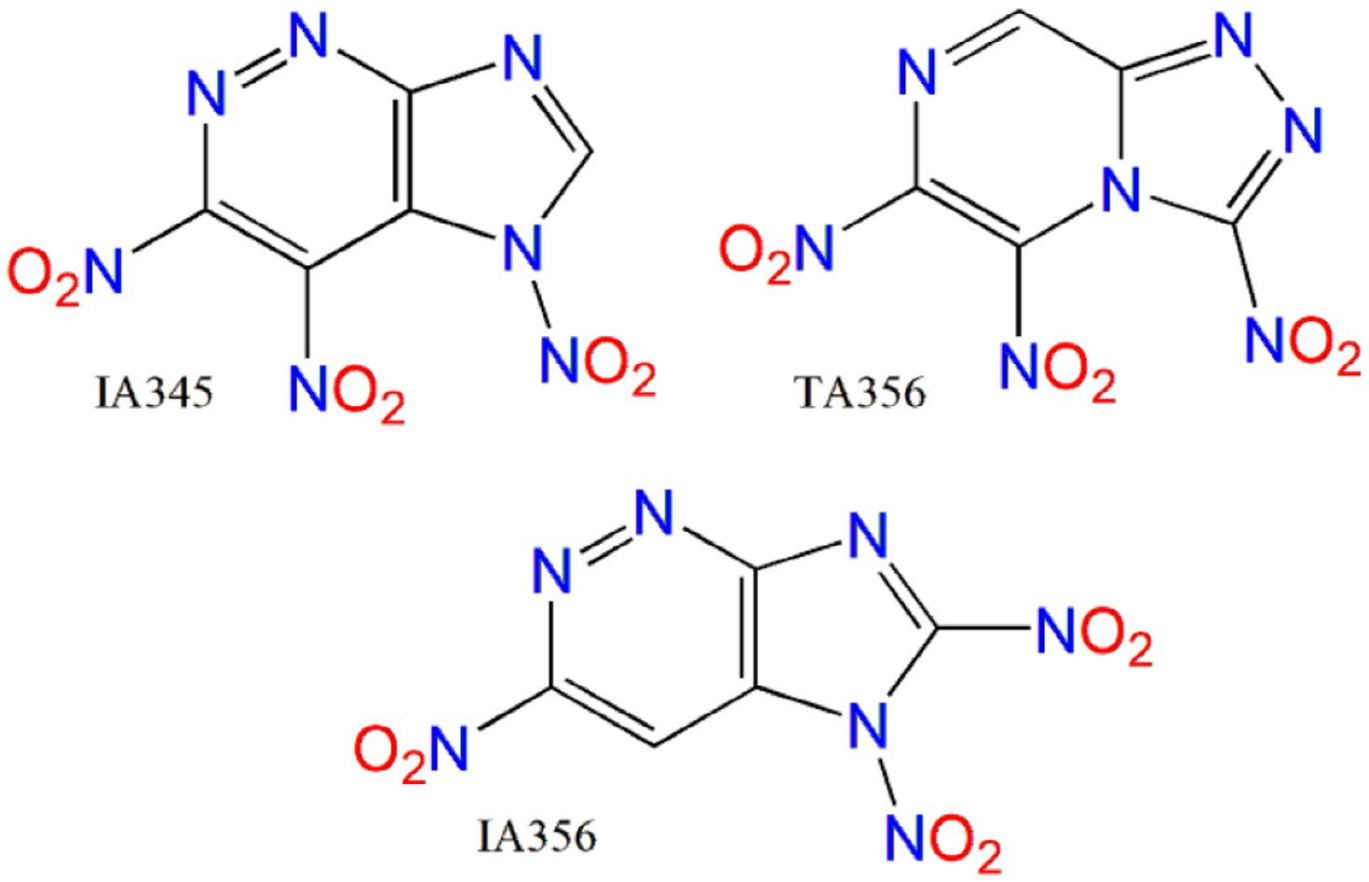
Promising candidates as energetic materials.

The combined analysis of MBO values, BDE calculations, and sensitivity data provides additional insight into the relative merits of the nine promising candidates. The nitrate ester candidates (ID346, TD358, TD568, TD356, TD368) present the lowest trigger bond MBO values (0.490–0.641) and undergo spontaneous rearrangement of the alkoxy radical to a carbonyl‐containing structure upon O—N homolysis, indicating a near‐barrierless decomposition pathway. Combined with the marginal impact sensitivity profiles of the nitrate ester series discussed in Section 3.4, these results indicate a less favorable stability profile relative to the nitro‐substituted candidates. Among the nitro‐substituted candidates, IA456 presents a BDE of 68.9 kJ mol^−1^, below the accepted stability threshold of 80 kJ mol^−1^ [81], reflecting the intrinsic lability of the N—NO_2_ linkage in this fused heterocyclic framework. Accordingly, IA345, IA356, and TA356 are identified, in Figure 9, as the most promising compounds, satisfying all performance, sensitivity, and thermal stability criteria established in this study.

## 4 Conclusions

This theoretical study of 5‐H‐imidazo‐[4,5‐c]‐pyridazine and 1,2,4‐triazolo‐[4,3‐a]‐pyrazine derivatives, functionalized with energetic groups (—NO_2_, —NHNO_2_, —N_3_, and —ONO_2_), demonstrated their potential as high‐performance energetic materials. DFT calculations at the B3LYP/def2‐TZVPP//B3LYP/6‐311G(d,p) level revealed that nitramino derivatives exhibited the highest chemical stability based on HOMO‐LUMO gap analysis, while substituent positioning significantly influenced stability through resonance effects.

Heat of formation followed the trend: —N_3_ > —NO_2_ > —NHNO_2_ > —ONO_2_. All derivatives showed densities exceeding TNT, with most I‐scaffold derivatives also surpassing RDX. Substitution at the ring nitrogen (I scaffold, position 5) generated N—NO_2_ linkages with higher density and detonation performance than C—NO_2_ analogues at the trisubstituted level, yielding the ranking: —ONO_2_ > —N—NO_2_ > —C—NO_2_ > —NHNO_2_ > —N_3_. For the fully carbon‐bonded T scaffold, the simpler order: —ONO_2_ > —NO_2_ > —NHNO_2_ > —N_3_ applies. This confirms that the ring atom bonded to the explosophore, not the explosophore identity alone, is the primary determinant of energetic performance.

Detonation performance increased with the number of substituents, with all derivatives exceeding TNT parameters. Notably, 18 compounds (ID346, ID345, IA3456, ID456, TA3568, ID356, TD358, TD568, TD356, TD368, ID3456, IB3456, TD3568, TB3568, IA456, IA356, IA345, and TA356) surpassed RDX in both detonation velocity and pressure. Impact sensitivity was assessed through two complementary approaches: the nitramine‐specific equation (Equation 14) was applied exclusively to nitramino derivatives, for which it is well‐parameterized; the non‐nitramine equation (Equation 15) was restricted to C‐nitro and N‐oxide derivatives, the only subclasses within its applicability domain; and the Politzer‐Murray $Q_{\mathrm{MAX}}$ criterion served as the sensitivity proxy for azido and nitrate ester compounds, for which no physically valid $h_{50}$ predictions could be obtained from the Pospíšil model. All nitramines showed lower sensitivity than RDX, and nitrate esters generally exhibited higher sensitivity than azides, consistent with established literature trends.

The same structural feature could be observed for the aromaticity trends. Explosophore substitution withdrew π‐electron density from the fused heterocyclic core, reducing NICS(1)_ZZ_ magnitude and increasing MEP polarization most strongly at ring‐nitrogen positions. Compounds with the greatest aromaticity loss consistently showed the highest density, detonation velocity and pressure, linking electronic depletion at the substitution site directly to detonation performance.

Mayer bond order analysis showed that N—NO_2_ trigger bonds are consistently more labile than C—NO_2_ bonds, and BDE calculations confirmed this: IA456 (N—NO_2_ at ring nitrogen) fell below the 80 kJ mol^−1^ stability threshold, while IA345, IA356, and TA356 (C—NO_2_ at carbon positions) remained well above it. Nitrate esters, with the lowest MBO values and barrierless O—N homolysis due to the spontaneous rearrangement of the alkoxy radical, prevented direct BDE calculation and showed the least favorable thermal stability profile overall. It is noted that MBO, as a static ground‐state descriptor, does not correctly identify the true trigger bond in azido derivatives, where thermal decomposition proceeds via N1—N2 cleavage despite C—N_3_ and N—N_3_ bonds displaying lower absolute bond orders, a limitation consistent with previous WBI‐based studies of organic azides.

After sequential screening relative to RDX, nine compounds were identified as promising candidates. Among these, the combined analysis of trigger bond lability and bond dissociation enthalpies further identified three compounds (IA345, IA356, and TA356) as presenting the most favorable balance between detonation performance and thermal stability. They exhibit heat of formation > 303.7 kJ mol^−1^, oxygen balance > −28.2%, density > 1.85 g cm^−3^, detonation velocity > 8.76 km s^−1^, detonation pressure > 33.17 GPa, bond dissociation enthalpies well above the accepted stability threshold of 80 kJ mol^−1^ and impact sensitivity comparable or lower than RDX. Overall, the position and chemical nature of the substitution site on the fused heterocyclic scaffold emerges as the unifying structural factor linking electronic structure, aromaticity, detonation performance, sensitivity, and thermal stability across the investigated compounds.

## Author Contributions

**Luciana Amorim da Silva:** conceptualization, software, investigation, writing – original draft. **Nathália Magalhães Paixão Rosa:** writing – review and editing. **Danillo Fernando Vianna Cantini:** software, resources. **Aline Cardoso Anastácio:** supervision, writing – review and editing.

## Funding

This work was supported by Fundação Carlos Chagas Filho de Amparo à Pesquisa do Estado do Rio de Janeiro, Coordenação de Aperfeiçoamento de Pessoal de Nível Superior (ROR: 00x0ma614), Univerzita Pardubice (SGS_2026_003), FAPERJ, CNPq.

## Conflicts of Interest

The authors declare no conflicts of interest.

## Supporting information


**Table S1:** DFT/B3LYP/6‐311G(d,p) optimized geometries of the designed compounds.
**Table S2:** Molecular electrostatic potential maps for the 5‐H‐imidazo[4,5‐c]‐pyridazine derivatives, mapped on the electron density surface at 0.001a.u. Red regions indicate electron‐rich corresponding to negative MEP values, while the blue region indicates electron‐deficient corresponding to positive MEP values.
**Table S3:** Molecular electrostatic potential maps for the 1,2,4‐triazolo[4,3‐a]‐pyrazine derivatives, mapped on the electron density surface at 0.001a.u. Red regions indicate electron‐rich corresponding to negative MEP values, while the blue region indicates electron‐deficient corresponding to positive MEP values.
**Figure S1:** A plot of the computed aromaticity index $\text{NICS}{\left(1\right)}_{\mathrm{zz}}$ of the 6‐member ring. Predicted densities (ρ), in g cm^−3^, maximum heat of detonation ($Q_{\mathrm{MAX}}$) in kcal kg^−1^, detonation velocities (D) in km s^−1^, and pressure ($P_{\mathrm{CJ}}$), in GPa, of 5‐H‐imidazo[4,5‐c]‐pyridazine derivatives (a) —NO_2_; (b) —NHNO_2_; (c) —N_3_ and (d) —ONO_2_.
**Figure S2:** A plot of the computed aromaticity index $\text{NICS}{\left(1\right)}_{\mathrm{zz}}$ of the 5‐member ring. Predicted densities (ρ), in g cm^−3^, maximum heat of detonation ($Q_{\mathrm{MAX}}$) in kcal kg^−1^, detonation velocities (D) in km s^−1^, and pressure ($P_{\mathrm{CJ}}$), in GPa, of 5‐H‐imidazo[4,5‐c]‐pyridazine derivatives (a) —NO_2_; (b) —NHNO_2_; (c) —N_3_ and (d) —ONO_2_.
**Figure S3:** A plot of the computed aromaticity index $\text{NICS}{\left(1\right)}_{\mathrm{zz}}$ of the 6‐member ring. Predicted densities (ρ), in g cm^−3^, maximum heat of detonation ($Q_{\mathrm{MAX}}$) in kcal kg^−1^, detonation velocities (D) in km s^−1^, and pressure ($P_{\mathrm{CJ}}$), in GPa, of 1,2,4‐triazolo[4,3‐a]‐pyrazine derivatives: (a) —NO_2_; (b) —NHNO_2_; (c) —N_3_ and (d) —ONO_2_.
**Figure S4:** A plot of the computed aromaticity index $\text{NICS}{\left(1\right)}_{\mathrm{zz}}$ of the 5‐member ring. Predicted densities (ρ), in g cm^−3^, maximum heat of detonation ($Q_{\mathrm{MAX}}$) in kcal kg^−1^, detonation velocities (D) in km s^−1^, and pressure ($P_{\mathrm{CJ}}$), in GPa, of 1,2,4‐triazolo[4,3‐a]‐pyrazine derivatives: (a) —NO_2_; (b) —NHNO_2_; (c) —N_3_ and (d) —ONO_2_.
**Table S4:** Calculated Mayer bond order of the designed compounds.

## Data Availability

The data that support the findings of this study are available from the corresponding author upon reasonable request.
