# Supplementary material for: Computational Design and Theoretical Evaluation of Novel Heterocyclic Energetic Materials Based on 5‐H‐Imidazo‐[4,5‐c]‐Pyridazine and 1,2,4‐Triazolo‐[4,3‐a]‐Pyrazine Scaffolds
Source: J Comput Chem. 2026 Sep 3;47(23):e70490. doi: 10.1002/jcc.70490 (PMC13542628; doi:10.1002/jcc.70490)
Supplement: Supplementary file 1 [file JCC-47-0-s001.docx]

**Supplementary Information**

**Computational Design and Theoretical Evaluation of Novel Heterocyclic Energetic Materials Based on 5-H-imidazo-[4,5-c] pyridazine and 1,2,4-triazolo-[4,3-a] pyrazine Scaffolds**

Luciana Amorim da Silva^a^, Nathália Magalhães Paixão Rosa^b^, Danillo Fernando Vianna Cantini^c^, Aline Cardoso Anastácio^a^*

a Chemical Engineering Department, Military Institute of Engineering, Rio de Janeiro, RJ, Brazil.

b Institute of Chemistry, Federal Rural University of Rio de Janeiro, Seropédica, RJ, 23890-000, Brazil.

c Institute of Energetic Materials, Faculty of Chemical Technology, University of Pardubice, Pardubice, Czech Republic

* Corresponding author.

E-mail address: [alinecardoso@ime.eb.br](mailto:alinecardoso@ime.eb.br)

Table of Contents

[Part A. Cartesian coordinates of the designed compounds	S3](#_Toc237786608)

[Part B. Molecular electrostatic potential maps for the 5-H-imidazo[4,5-c]-pyridazine and 1,2,4-triazolo[4,3-a]-pyrazine derivatives	S18](#_Toc237786609)

[Part C. Computed aromaticity index $NICS1zz$ of the 6-member ring and 5-member ring of of 5-H-imidazo[4,5-c]-pyridazine and 1,2,4-triazolo[4,3-a]-pyrazine derivatives	S26](#_Toc237786610)

[Part D. Identification of Trigger Bonds in the designed compounds through Mayer Bond Order	S30](#_Toc237786611)

# Part A. Cartesian coordinates of the designed compounds

**Table S1**. DFT/B3LYP/6-311G(d,p) optimized geometries of the designed compounds.

| **IA34** | | | |
| --- | --- | --- | --- |
| **Atom** | **X, Å** | **Y, Å** | **Z, Å** |
| C | 0.12347 | 0.44928 | -0.00002 |
| C | 0.86926 | -0.73165 | -0.00001 |
| C | -1.24420 | 0.24488 | 0.00000 |
| C | -1.72739 | -1.09923 | 0.00000 |
| N | -0.94132 | -2.16550 | -0.00000 |
| N | 0.36983 | -1.96016 | -0.00000 |
| N | -2.35368 | 1.02374 | 0.00001 |
| C | -3.43852 | 0.15559 | 0.00001 |
| N | -3.10959 | -1.10111 | 0.00001 |
| H | -2.34517 | 2.03406 | 0.00002 |
| H | -4.44991 | 0.53554 | 0.00002 |
| N | 2.36038 | -0.72859 | 0.00001 |
| N | 0.68087 | 1.80424 | -0.00002 |
| O | 2.89185 | -0.74955 | -1.09108 |
| O | 2.89181 | -0.74941 | 1.09112 |
| O | 1.88867 | 1.93012 | -0.00001 |
| O | -0.14061 | 2.71994 | -0.00003 |

| **IA345** | | | |
| --- | --- | --- | --- |
| **Atom** | **X, Å** | **Y, Å** | **Z, Å** |
| C | -0.52665 | -0.19568 | 0.03553 |
| C | -1.67255 | 0.60234 | -0.01934 |
| C | 0.64872 | 0.52698 | 0.03160 |
| C | 0.55518 | 1.94907 | 0.05640 |
| N | -0.58346 | 2.62656 | 0.03511 |
| N | -1.70161 | 1.92455 | -0.02622 |
| N | 2.01165 | 0.29222 | 0.07662 |
| C | 2.64677 | 1.54532 | 0.11373 |
| N | 1.81461 | 2.52956 | 0.11165 |
| H | 3.72424 | 1.60068 | 0.13696 |
| N | -3.02755 | -0.02784 | -0.00564 |
| N | -0.58701 | -1.67569 | 0.12261 |
| O | -3.86002 | 0.43764 | -0.74907 |
| O | -3.16409 | -0.95252 | 0.78097 |
| O | -1.07722 | -2.25361 | -0.82379 |
| O | -0.12707 | -2.15594 | 1.13927 |
| N | 2.72337 | -0.94075 | -0.20617 |
| O | 3.91103 | -0.88696 | -0.01236 |
| O | 2.04449 | -1.84726 | -0.61004 |

| **IA3456** | | | |
| --- | --- | --- | --- |
| **Atom** | **X, Å** | **Y, Å** | **Z, Å** |
| C | 1.24324 | 0.19676 | -0.00369 |
| C | 2.22841 | -0.78753 | 0.00016 |
| C | -0.05472 | -0.29779 | 0.00516 |
| C | -0.20654 | -1.72209 | 0.01664 |
| N | 0.79895 | -2.58868 | 0.00875 |
| N | 2.02237 | -2.10168 | 0.00488 |
| N | -1.33414 | 0.18299 | -0.00890 |
| C | -2.15204 | -0.93387 | 0.00541 |
| N | -1.53933 | -2.06722 | 0.01754 |
| N | 1.54120 | 1.63881 | -0.03994 |
| N | -1.82549 | 1.73404 | -0.00400 |
| O | 2.57799 | 1.98488 | -0.56459 |
| O | 0.69591 | 2.36648 | 0.46343 |
| O | -2.20569 | 2.08339 | 1.05494 |
| O | -1.70851 | 2.23845 | -1.06424 |
| N | -3.61896 | -0.79746 | -0.04914 |
| O | -4.02848 | 0.32063 | -0.33819 |
| O | -4.27162 | -1.78737 | 0.17908 |
| N | 3.67781 | -0.43983 | 0.07797 |
| O | 4.02647 | 0.10783 | 1.10781 |
| O | 4.36304 | -0.77175 | -0.86228 |

| **IA346** | | | |
| --- | --- | --- | --- |
| **Atom** | **X, Å** | **Y, Å** | **Z, Å** |
| C | -0.97199 | 0.40976 | 0.00002 |
| C | -1.83553 | -0.68344 | 0.00001 |
| C | 0.36819 | 0.05967 | 0.00001 |
| C | 0.70255 | -1.32824 | 0.00001 |
| N | -0.19299 | -2.30783 | -0.00000 |
| N | -1.46835 | -1.96392 | 0.00000 |
| N | 1.55442 | 0.71771 | 0.00000 |
| C | 2.51290 | -0.27140 | -0.00000 |
| N | 2.07550 | -1.48459 | 0.00000 |
| N | -3.31814 | -0.52721 | 0.00006 |
| N | -1.38076 | 1.81999 | -0.00007 |
| O | -3.84544 | -0.49420 | 1.09194 |
| O | -3.84558 | -0.49473 | -1.09175 |
| O | -2.56799 | 2.07071 | -0.00030 |
| O | -0.46366 | 2.63670 | 0.00009 |
| N | 3.92951 | 0.12882 | -0.00000 |
| O | 4.76021 | -0.74910 | -0.00001 |
| O | 4.11807 | 1.34088 | 0.00001 |
| H | 1.70406 | 1.71887 | 0.00002 |

| **IA35** | | | |
| --- | --- | --- | --- |
| **Atom** | **X, Å** | **Y, Å** | **Z, Å** |
| C | -0.58398 | -0.65678 | -0.00001 |
| C | -1.77957 | 0.06212 | 0.00001 |
| C | 0.50970 | 0.17880 | 0.00000 |
| C | 0.32136 | 1.58426 | 0.00005 |
| N | -0.86956 | 2.17055 | 0.00008 |
| N | -1.93079 | 1.37669 | 0.00005 |
| N | 1.88887 | 0.03844 | -0.00001 |
| C | 2.44132 | 1.32968 | 0.00002 |
| N | 1.54132 | 2.25463 | 0.00006 |
| H | 3.51232 | 1.46037 | 0.00002 |
| N | -3.06437 | -0.72233 | -0.00001 |
| N | 2.63696 | -1.17793 | -0.00005 |
| O | -4.10719 | -0.10915 | -0.00039 |
| O | -2.93750 | -1.94107 | 0.00038 |
| O | 1.97833 | -2.19126 | -0.00008 |
| O | 3.83781 | -1.04534 | -0.00006 |
| H | -0.55399 | -1.73457 | -0.00001 |

| **IA356** | | | |
| --- | --- | --- | --- |
| **Atom** | **X, Å** | **Y, Å** | **Z, Å** |
| C | 1.45328 | 0.67989 | 0.05769 |
| C | 2.49426 | -0.24635 | 0.00889 |
| C | 0.22306 | 0.06032 | 0.04339 |
| C | 0.15402 | -1.35139 | -0.02812 |
| N | 1.21335 | -2.14961 | -0.06639 |
| N | 2.40124 | -1.56631 | -0.04688 |
| N | -1.10817 | 0.46639 | 0.07629 |
| C | -1.86265 | -0.70988 | -0.00842 |
| N | -1.16828 | -1.78551 | -0.05146 |
| N | 3.90335 | 0.28805 | 0.02538 |
| N | -1.57128 | 1.82928 | -0.16097 |
| O | 4.81257 | -0.50824 | -0.00001 |
| O | 4.00107 | 1.50812 | 0.06293 |
| O | -2.71130 | 1.93023 | -0.52265 |
| O | -0.73686 | 2.67449 | 0.03521 |
| N | -3.33346 | -0.71602 | 0.13979 |
| O | -3.95706 | -1.33068 | -0.69542 |
| O | -3.75283 | -0.13674 | 1.12500 |
| H | 1.62613 | 1.74308 | 0.10866 |

| **IA36** | | | |
| --- | --- | --- | --- |
| **Atom** | **X, Å** | **Y, Å** | **Z, Å** |
| C | -1.19557 | -0.97318 | 0.00001 |
| C | -2.13922 | 0.04626 | -0.00000 |
| C | 0.09466 | -0.47921 | 0.00001 |
| C | 0.30038 | 0.92970 | -0.00002 |
| N | -0.68551 | 1.82759 | -0.00004 |
| N | -1.91770 | 1.35803 | -0.00003 |
| N | 1.35391 | -1.02039 | 0.00002 |
| C | 2.20468 | 0.05575 | 0.00000 |
| N | 1.65063 | 1.22229 | -0.00002 |
| N | -3.59115 | -0.34748 | 0.00001 |
| N | 3.65212 | -0.20317 | 0.00001 |
| O | -4.42183 | 0.53150 | 0.00012 |
| O | -3.81008 | -1.55392 | -0.00011 |
| O | 3.96035 | -1.39270 | 0.00003 |
| O | 4.39729 | 0.74856 | -0.00001 |
| H | -1.48186 | -2.01498 | 0.00002 |
| H | 1.65016 | -1.98662 | 0.00004 |

| **IA45** | | | |
| --- | --- | --- | --- |
| **Atom** | **X, Å** | **Y, Å** | **Z, Å** |
| C | -1.13649 | 0.49829 | 0.00110 |
| C | -2.43635 | 0.00698 | -0.12564 |
| C | -0.13596 | -0.45163 | 0.02393 |
| C | -0.54695 | -1.81235 | 0.01626 |
| N | -1.80479 | -2.21813 | -0.06487 |
| N | -2.75127 | -1.29341 | -0.16245 |
| N | 1.23869 | -0.51652 | 0.14247 |
| C | 1.58003 | -1.88458 | 0.17735 |
| N | 0.55445 | -2.66013 | 0.12113 |
| H | 2.61746 | -2.17481 | 0.24120 |
| N | -0.92683 | 1.94010 | 0.22105 |
| N | 2.20718 | 0.49864 | -0.23609 |
| O | -1.68974 | 2.70505 | -0.34169 |
| O | -0.03497 | 2.24162 | 0.99829 |
| O | 1.76782 | 1.44283 | -0.83673 |
| O | 3.34212 | 0.24568 | 0.08444 |
| H | -3.26699 | 0.69923 | -0.18227 |

| **IA456** | | | |
| --- | --- | --- | --- |
| **Atom** | **X, Å** | **Y, Å** | **Z, Å** |
| C | -1.93754 | 0.15027 | -0.00005 |
| C | -2.79009 | 1.24773 | -0.00001 |
| C | -0.57450 | 0.42830 | -0.00002 |
| C | -0.22885 | 1.81683 | 0.00004 |
| N | -1.09946 | 2.82109 | 0.00006 |
| N | -2.38591 | 2.53071 | 0.00003 |
| N | 0.62765 | -0.22531 | -0.00004 |
| C | 1.59294 | 0.76904 | 0.00008 |
| N | 1.14044 | 1.97503 | 0.00008 |
| N | -2.49295 | -1.21105 | -0.00013 |
| N | 0.90537 | -1.81825 | 0.00009 |
| O | -3.70266 | -1.33260 | 0.00027 |
| O | -1.67939 | -2.12687 | -0.00061 |
| O | 0.98169 | -2.26347 | 1.09057 |
| O | 0.98224 | -2.26345 | -1.09036 |
| N | 3.02726 | 0.43582 | 0.00003 |
| O | 3.28960 | -0.76170 | 0.00043 |
| O | 3.80750 | 1.35831 | -0.00042 |
| H | -3.86048 | 1.08896 | -0.00001 |

| **IA46** | | | |
| --- | --- | --- | --- |
| **Atom** | **X, Å** | **Y, Å** | **Z, Å** |
| C | -1.75597 | -0.05205 | -0.00000 |
| C | -2.48563 | 1.13051 | 0.00000 |
| C | -0.37979 | 0.08732 | 0.00000 |
| C | 0.13608 | 1.41465 | 0.00000 |
| N | -0.61638 | 2.51034 | 0.00000 |
| N | -1.93129 | 2.35806 | 0.00000 |
| N | 0.70539 | -0.72921 | -0.00000 |
| C | 1.78888 | 0.12002 | 0.00000 |
| N | 1.52035 | 1.38202 | 0.00000 |
| N | -2.39115 | -1.37276 | -0.00000 |
| N | 3.13559 | -0.46701 | 0.00000 |
| O | -3.60622 | -1.42595 | -0.00000 |
| O | -1.62687 | -2.33550 | 0.00000 |
| O | 3.16181 | -1.69422 | 0.00000 |
| O | 4.08014 | 0.28913 | -0.00001 |
| H | -3.56759 | 1.10030 | 0.00000 |
| H | 0.71776 | -1.74070 | 0.00000 |

| **IA56** | | | |
| --- | --- | --- | --- |
| **Atom** | **X, Å** | **Y, Å** | **Z, Å** |
| C | 2.18076 | 1.15308 | 0.10758 |
| C | 3.38172 | 0.44123 | 0.04972 |
| C | 1.06754 | 0.34048 | 0.07379 |
| C | 1.24022 | -1.05677 | -0.02595 |
| N | 2.41783 | -1.66273 | -0.07214 |
| N | 3.49808 | -0.89506 | -0.03314 |
| N | -0.31682 | 0.51627 | 0.10380 |
| C | -0.86120 | -0.77176 | -0.01642 |
| N | 0.00631 | -1.71023 | -0.06998 |
| N | -0.99059 | 1.76961 | -0.16143 |
| N | -2.30714 | -1.03032 | 0.13407 |
| O | -0.32460 | 2.75095 | 0.06203 |
| O | -2.11763 | 1.68126 | -0.57171 |
| O | -2.83433 | -0.48842 | 1.08971 |
| O | -2.80818 | -1.78729 | -0.66740 |
| H | 4.32871 | 0.96780 | 0.07714 |
| H | 2.14128 | 2.22967 | 0.18123 |

| **IB34** | | | |
| --- | --- | --- | --- |
| **Atom** | **X, Å** | **Y, Å** | **Z, Å** |
| C | -0.11299 | 0.12593 | -0.06012 |
| C | 0.66497 | -1.01210 | -0.35419 |
| C | -1.45885 | -0.18019 | 0.11444 |
| C | -1.88313 | -1.53438 | -0.00705 |
| N | -1.07451 | -2.55292 | -0.28704 |
| N | 0.19963 | -2.26411 | -0.45349 |
| N | -2.59436 | 0.51551 | 0.42226 |
| C | -3.62119 | -0.40695 | 0.44753 |
| N | -3.24204 | -1.63032 | 0.21584 |
| H | -2.66826 | 1.52309 | 0.40631 |
| H | -4.63704 | -0.09918 | 0.64975 |
| N | 3.02325 | -0.62375 | 0.25841 |
| N | -0.04986 | 2.59064 | -0.24666 |
| O | 4.11689 | -1.10844 | 0.07962 |
| O | 2.70319 | 0.15933 | 1.14811 |
| O | -1.19924 | 2.60121 | -0.67856 |
| O | 0.67207 | 3.55334 | -0.07642 |
| N | 0.49689 | 1.37342 | 0.09211 |
| H | 1.44929 | 1.42811 | 0.44578 |
| N | 2.04546 | -0.93324 | -0.70710 |
| H | 2.34863 | -1.77601 | -1.18797 |

| **IB345** | | | |
| --- | --- | --- | --- |
| **Atom** | **X, Å** | **Y, Å** | **Z, Å** |
| C | -0.54002 | 0.16095 | 0.09090 |
| C | -1.68759 | 0.81714 | -0.38921 |
| C | 0.54985 | 1.00446 | 0.19858 |
| C | 0.42565 | 2.36830 | -0.17432 |
| N | -0.69206 | 2.90093 | -0.65247 |
| N | -1.74485 | 2.10922 | -0.73968 |
| N | 1.83943 | 0.89758 | 0.67572 |
| C | 2.41245 | 2.16552 | 0.54949 |
| N | 1.61071 | 3.04968 | 0.05183 |
| H | 3.43728 | 2.31937 | 0.85360 |
| N | -3.67891 | -0.38172 | 0.41432 |
| N | -0.02453 | -2.12986 | -0.53654 |
| O | -4.86997 | -0.43966 | 0.21707 |
| O | -3.07421 | -0.77606 | 1.40420 |
| O | 0.78001 | -1.70142 | -1.33895 |
| O | -0.44819 | -3.25734 | -0.42636 |
| N | -0.48966 | -1.20748 | 0.45929 |
| H | -1.34959 | -1.56860 | 0.86921 |
| N | -2.90633 | 0.13359 | -0.65112 |
| H | -3.52459 | 0.67819 | -1.24669 |
| N | 2.52110 | -0.19875 | 1.13661 |
| N | 3.21683 | -0.96918 | 0.06875 |
| H | 1.91868 | -0.86106 | 1.61962 |
| O | 3.57510 | -0.34151 | -0.89052 |
| O | 3.41152 | -2.12080 | 0.36762 |

| **IB3456** | | | |
| --- | --- | --- | --- |
| **Atom** | **X, Å** | **Y, Å** | **Z, Å** |
| C | -1.21653 | -0.18881 | -0.20652 |
| C | -2.17312 | -1.22176 | -0.28167 |
| C | 0.07108 | -0.66863 | -0.02956 |
| C | 0.29301 | -2.06849 | 0.02055 |
| N | -0.66550 | -2.97945 | -0.08840 |
| N | -1.89851 | -2.53069 | -0.22291 |
| N | 1.32655 | -0.11245 | 0.12453 |
| C | 2.20977 | -1.18785 | 0.24971 |
| N | 1.63762 | -2.34768 | 0.18628 |
| N | -1.17801 | 1.97055 | -1.34637 |
| N | 1.24457 | 1.79481 | 1.52996 |
| O | -1.87983 | 2.92903 | -1.57102 |
| O | -0.14774 | 1.64934 | -1.92698 |
| O | 1.02411 | 1.03960 | 2.44489 |
| O | 1.21224 | 3.00139 | 1.50003 |
| N | 1.69346 | 1.19318 | 0.27680 |
| N | -1.56201 | 1.17668 | -0.25241 |
| N | 3.55655 | -0.97984 | 0.51312 |
| N | 4.38684 | -0.41852 | -0.52151 |
| O | 3.82464 | 0.17803 | -1.41135 |
| O | 5.57103 | -0.58680 | -0.35105 |
| H | 4.03371 | -1.77761 | 0.91889 |
| H | 1.40552 | 1.78598 | -0.50137 |
| H | -2.49224 | 1.42434 | 0.07887 |
| N | -3.54927 | -0.98113 | -0.55244 |
| N | -4.37624 | -0.34879 | 0.40556 |
| H | -4.02028 | -1.83216 | -0.84879 |
| O | -3.85933 | 0.51320 | 1.10572 |
| O | -5.53815 | -0.67928 | 0.39376 |

| **IB346** | | | |
| --- | --- | --- | --- |
| **Atom** | **X, Å** | **Y, Å** | **Z, Å** |
| C | 0.98858 | 0.06089 | -0.10216 |
| C | 1.95053 | -0.94513 | -0.34442 |
| C | -0.30136 | -0.44537 | -0.03286 |
| C | -0.51017 | -1.84302 | -0.20240 |
| N | 0.45648 | -2.72494 | -0.42961 |
| N | 1.68565 | -2.24637 | -0.49071 |
| N | -1.54728 | 0.08059 | 0.20250 |
| C | -2.41252 | -0.98444 | 0.12962 |
| N | -1.85164 | -2.14116 | -0.09006 |
| N | 4.16638 | -0.21644 | 0.45720 |
| N | 0.71266 | 2.49258 | -0.39554 |
| O | 5.33028 | -0.53195 | 0.36564 |
| O | 3.66233 | 0.49562 | 1.31967 |
| O | -0.37131 | 2.30664 | -0.93588 |
| O | 1.27745 | 3.55500 | -0.23149 |
| N | 1.37308 | 1.38638 | 0.10728 |
| H | 2.28806 | 1.59166 | 0.49949 |
| N | 3.32529 | -0.65148 | -0.58769 |
| H | 3.78973 | -1.42990 | -1.04782 |
| N | -3.77880 | -0.89584 | 0.33522 |
| N | -4.54067 | 0.26823 | 0.25216 |
| O | -3.93844 | 1.33004 | 0.23736 |
| O | -5.74042 | 0.09486 | 0.23840 |
| H | -1.81120 | 1.05721 | 0.17901 |
| H | -4.32423 | -1.73930 | 0.20786 |

| **IB35** | | | |
| --- | --- | --- | --- |
| **Atom** | **X, Å** | **Y, Å** | **Z, Å** |
| C | 0.53400 | -0.18581 | -0.06632 |
| C | 1.65629 | 0.57972 | 0.27339 |
| C | -0.59118 | 0.58779 | -0.23408 |
| C | -0.54256 | 1.98725 | -0.03251 |
| N | 0.56444 | 2.63229 | 0.31098 |
| N | 1.66399 | 1.91392 | 0.44882 |
| N | -1.90651 | 0.34156 | -0.59087 |
| C | -2.56475 | 1.57734 | -0.55151 |
| N | -1.79195 | 2.56304 | -0.23771 |
| H | -3.61969 | 1.62601 | -0.77929 |
| N | 3.49450 | -1.03065 | -0.14073 |
| N | -2.78542 | -1.66310 | 0.37003 |
| O | 4.70475 | -1.11289 | -0.05050 |
| O | 2.74518 | -1.79744 | -0.72370 |
| O | -2.88858 | -1.06657 | 1.41008 |
| O | -2.92810 | -2.83878 | 0.13139 |
| N | -2.52844 | -0.84729 | -0.83484 |
| N | 2.92372 | 0.03456 | 0.55677 |
| H | 3.62823 | 0.72143 | 0.80370 |
| H | 0.58010 | -1.25791 | -0.17817 |
| H | -2.04572 | -1.45214 | -1.49532 |

| **IB356** | | | |
| --- | --- | --- | --- |
| **Atom** | **X, Å** | **Y, Å** | **Z, Å** |
| C | 1.48754 | 0.20388 | 0.01542 |
| C | 2.49718 | -0.72461 | -0.27155 |
| C | 0.23081 | -0.33210 | -0.13679 |
| C | 0.06422 | -1.66881 | -0.56510 |
| N | 1.07849 | -2.47821 | -0.83627 |
| N | 2.29596 | -1.99364 | -0.67252 |
| N | -1.06056 | 0.14492 | 0.02925 |
| C | -1.90682 | -0.92128 | -0.30234 |
| N | -1.28216 | -2.00057 | -0.64354 |
| N | 4.48570 | 0.40966 | 0.68265 |
| N | -1.24155 | 2.36345 | -0.81429 |
| O | 5.68626 | 0.25358 | 0.79510 |
| O | 3.79137 | 1.23302 | 1.25545 |
| O | -1.11948 | 1.89929 | -1.92015 |
| O | -1.25498 | 3.51734 | -0.45599 |
| N | -1.48659 | 1.41386 | 0.26886 |
| N | 3.87095 | -0.42298 | -0.25456 |
| H | 4.47533 | -1.20031 | -0.49846 |
| N | -3.28650 | -0.75443 | -0.32957 |
| N | -3.98361 | -0.56938 | 0.91209 |
| O | -3.33042 | -0.18033 | 1.85587 |
| O | -5.17071 | -0.79004 | 0.85609 |
| H | -3.78364 | -1.44114 | -0.88629 |
| H | 1.70362 | 1.21654 | 0.31766 |
| H | -1.18017 | 1.83090 | 1.14356 |

| **IB36** | | | |
| --- | --- | --- | --- |
| **Atom** | **X, Å** | **Y, Å** | **Z, Å** |
| C | 1.19372 | -0.49258 | 0.35590 |
| C | 2.17851 | 0.48925 | 0.19016 |
| C | -0.07918 | 0.01175 | 0.21311 |
| C | -0.27490 | 1.39463 | -0.04590 |
| N | 0.71930 | 2.26414 | -0.16556 |
| N | 1.94923 | 1.79029 | -0.05631 |
| N | -1.34568 | -0.52373 | 0.27462 |
| C | -2.20040 | 0.52889 | 0.03767 |
| N | -1.62503 | 1.68050 | -0.15392 |
| N | 4.21584 | -0.88782 | -0.12339 |
| N | -4.30949 | -0.75251 | -0.07511 |
| O | 5.41602 | -0.75990 | -0.28351 |
| O | 3.55736 | -1.90336 | -0.27792 |
| O | -3.70058 | -1.80538 | 0.06396 |
| O | -5.49630 | -0.60943 | -0.28001 |
| N | -3.58445 | 0.42433 | 0.05683 |
| N | 3.56072 | 0.24996 | 0.34770 |
| H | 4.13864 | 1.07302 | 0.21477 |
| H | 1.43365 | -1.52337 | 0.56341 |
| H | -1.63832 | -1.48293 | 0.39189 |
| H | -4.11556 | 1.25027 | -0.18990 |

| **IB45** | | | |
| --- | --- | --- | --- |
| **Atom** | **X, Å** | **Y, Å** | **Z, Å** |
| C | 0.52678 | 1.14366 | -0.38588 |
| C | 1.62238 | 1.93562 | -0.75201 |
| C | 0.87202 | -0.10228 | 0.10821 |
| C | 2.25186 | -0.42413 | 0.19762 |
| N | 3.23866 | 0.38487 | -0.17026 |
| N | 2.90672 | 1.57230 | -0.65572 |
| N | 0.22666 | -1.22917 | 0.58166 |
| C | 1.22583 | -2.14952 | 0.90167 |
| N | 2.42271 | -1.70848 | 0.69829 |
| H | 0.94778 | -3.12085 | 1.28408 |
| N | -1.63830 | 1.90522 | 0.47902 |
| N | -1.74380 | -1.78249 | -0.62899 |
| O | -2.46804 | 2.76355 | 0.28246 |
| O | -1.48652 | 1.23708 | 1.49318 |
| O | -1.02859 | -2.19897 | -1.50570 |
| O | -2.94041 | -1.61185 | -0.62660 |
| N | -1.11018 | -1.50639 | 0.66811 |
| N | -0.79819 | 1.60172 | -0.61449 |
| H | -1.63645 | -0.75075 | 1.10846 |
| H | 1.45987 | 2.92871 | -1.16016 |
| H | -0.90598 | 2.34106 | -1.30002 |

| **IB456** | | | |
| --- | --- | --- | --- |
| **Atom** | **X, Å** | **Y, Å** | **Z, Å** |
| C | 1.97471 | 0.70323 | 0.04327 |
| C | 2.69936 | 1.90286 | 0.07102 |
| C | 0.60237 | 0.87634 | 0.09306 |
| C | 0.10087 | 2.20134 | 0.14764 |
| N | 0.85342 | 3.29465 | 0.16938 |
| N | 2.16793 | 3.12834 | 0.13761 |
| N | -0.52350 | 0.07206 | 0.11980 |
| C | -1.61296 | 0.94457 | 0.17507 |
| N | -1.28567 | 2.19592 | 0.18315 |
| N | 2.56536 | -1.41392 | -1.04594 |
| N | -0.19441 | -1.79037 | 1.54819 |
| O | 3.51895 | -2.13906 | -1.21426 |
| O | 1.52514 | -1.38810 | -1.68639 |
| O | -0.22917 | -1.01726 | 2.47453 |
| O | 0.09211 | -2.96432 | 1.53601 |
| N | -0.62781 | -1.28231 | 0.25008 |
| N | 2.64977 | -0.54263 | 0.06561 |
| N | -2.90932 | 0.46194 | 0.30685 |
| N | -3.51078 | -0.22007 | -0.80719 |
| O | -2.76155 | -0.67608 | -1.64118 |
| O | -4.71676 | -0.28853 | -0.75479 |
| H | -3.57207 | 1.13643 | 0.67348 |
| H | -0.13905 | -1.79637 | -0.48184 |
| H | 3.78475 | 1.87840 | 0.04975 |
| H | 3.61557 | -0.52703 | 0.37400 |

| **IB46** | | | |
| --- | --- | --- | --- |
| **Atom** | **X, Å** | **Y, Å** | **Z, Å** |
| C | -1.96242 | 0.29013 | -0.17626 |
| C | -2.79299 | 1.41045 | -0.00750 |
| C | -0.60985 | 0.58361 | -0.12231 |
| C | -0.23244 | 1.94091 | 0.08088 |
| N | -1.08041 | 2.95449 | 0.21490 |
| N | -2.37504 | 2.66610 | 0.17052 |
| N | 0.56968 | -0.10854 | -0.25459 |
| C | 1.56003 | 0.82830 | -0.09086 |
| N | 1.14583 | 2.04943 | 0.09594 |
| N | -2.16257 | -2.15929 | 0.10027 |
| N | 3.50289 | -0.69628 | -0.01568 |
| O | -2.97912 | -3.05576 | 0.02637 |
| O | -1.04236 | -2.21512 | 0.58198 |
| O | 2.76640 | -1.67003 | -0.05708 |
| O | 4.70873 | -0.68539 | 0.11864 |
| N | 2.91692 | 0.55454 | -0.18602 |
| N | -2.55034 | -0.95116 | -0.48705 |
| H | -3.87220 | 1.29012 | -0.03085 |
| H | -3.54777 | -0.97200 | -0.65980 |
| H | 0.69419 | -1.11173 | -0.24335 |
| H | 3.55387 | 1.31855 | 0.00294 |

| **IB56** | | | |
| --- | --- | --- | --- |
| **Atom** | **X, Å** | **Y, Å** | **Z, Å** |
| C | 2.41103 | 0.67722 | -0.78407 |
| C | 3.61932 | -0.00399 | -0.61549 |
| C | 1.32581 | -0.00845 | -0.28643 |
| C | 1.52001 | -1.27153 | 0.31469 |
| N | 2.70336 | -1.85968 | 0.43541 |
| N | 3.75902 | -1.20889 | -0.03850 |
| N | -0.04284 | 0.21374 | -0.20614 |
| C | -0.57446 | -0.91572 | 0.42490 |
| N | 0.30669 | -1.80872 | 0.73510 |
| N | -0.48466 | 2.48191 | 0.36390 |
| N | -2.87746 | -1.12373 | -0.32280 |
| O | -0.78873 | 3.54145 | -0.13465 |
| O | -0.05917 | 2.24310 | 1.46598 |
| O | -2.51998 | -0.77504 | -1.42772 |
| O | -3.95917 | -1.54114 | 0.02113 |
| N | -1.92791 | -0.98579 | 0.74224 |
| N | -0.75355 | 1.33559 | -0.49675 |
| H | 4.54884 | 0.43480 | -0.95964 |
| H | -2.15364 | -1.65427 | 1.47079 |
| H | 2.36143 | 1.65216 | -1.25337 |
| H | -0.72903 | 1.64409 | -1.46453 |

| **IC34** | | | |
| --- | --- | --- | --- |
| **Atom** | **X, Å** | **Y, Å** | **Z, Å** |
| C | 0.05904 | 0.67086 | 0.00014 |
| C | -0.73604 | -0.49500 | 0.00004 |
| C | 1.41396 | 0.38054 | 0.00008 |
| C | 1.85682 | -0.96459 | -0.00011 |
| N | 1.03571 | -2.01191 | -0.00023 |
| N | -0.25779 | -1.74965 | -0.00015 |
| N | 2.55440 | 1.13683 | 0.00015 |
| C | 3.61012 | 0.23913 | 0.00001 |
| N | 3.24186 | -1.00770 | -0.00015 |
| H | 2.60498 | 2.14342 | 0.00029 |
| H | 4.63323 | 0.58653 | 0.00004 |
| N | -2.12930 | -0.31804 | 0.00018 |
| N | -2.82801 | -1.34381 | 0.00012 |
| N | -3.58367 | -2.17937 | 0.00010 |
| N | -0.32954 | 2.01468 | 0.00038 |
| N | -1.52276 | 2.35526 | -0.00011 |
| N | -2.53256 | 2.85864 | -0.00049 |

| **IC3436** | | | |
| --- | --- | --- | --- |
| **Atom** | **X, Å** | **Y, Å** | **Z, Å** |
| C | -1.10120 | 0.33277 | -0.08710 |
| C | -2.02136 | -0.74013 | 0.02155 |
| C | 0.20988 | -0.10698 | -0.07005 |
| C | 0.50316 | -1.48591 | 0.06788 |
| N | -0.42362 | -2.42851 | 0.18130 |
| N | -1.68535 | -2.02788 | 0.14658 |
| N | 1.44432 | 0.51779 | -0.11200 |
| C | 2.38829 | -0.50910 | -0.06204 |
| N | 1.87277 | -1.69511 | 0.07137 |
| N | -1.35642 | 1.70288 | -0.17991 |
| N | -2.50911 | 2.15481 | -0.26482 |
| N | -3.45993 | 2.75548 | -0.35159 |
| N | 1.71475 | 1.80465 | -0.61452 |
| N | 1.70011 | 2.67467 | 0.29842 |
| N | 1.71133 | 3.58912 | 0.95424 |
| N | 3.71823 | -0.14611 | -0.13388 |
| N | 4.53270 | -1.08783 | -0.10024 |
| N | 5.37513 | -1.83148 | -0.08118 |
| N | -3.38470 | -0.40635 | 0.00230 |
| N | -4.19451 | -1.34161 | 0.10552 |
| N | -5.03751 | -2.08365 | 0.18963 |

| **IC345** | | | |
| --- | --- | --- | --- |
| **Atom** | **X, Å** | **Y, Å** | **Z, Å** |
| C | -0.23554 | 0.27374 | -0.11070 |
| C | -1.49207 | -0.36103 | 0.01510 |
| C | 0.80939 | -0.63771 | -0.11566 |
| C | 0.54971 | -2.02396 | 0.01322 |
| N | -0.67007 | -2.53722 | 0.14250 |
| N | -1.67664 | -1.68462 | 0.13467 |
| N | 2.18450 | -0.54930 | -0.18472 |
| C | 2.66514 | -1.85504 | -0.15396 |
| N | 1.73304 | -2.74596 | -0.01785 |
| H | 3.72623 | -2.03873 | -0.23840 |
| N | -2.62412 | 0.46763 | 0.02055 |
| N | -3.72852 | -0.08812 | 0.13615 |
| N | -4.79037 | -0.45029 | 0.23342 |
| N | 0.05583 | 1.63688 | -0.20646 |
| N | -0.83493 | 2.49984 | -0.26061 |
| N | -1.48308 | 3.42071 | -0.32289 |
| N | 2.96519 | 0.54557 | -0.60189 |
| N | 3.08719 | 1.41413 | 0.30333 |
| N | 3.28113 | 2.30828 | 0.95955 |

| **IC346** | | | |
| --- | --- | --- | --- |
| **Atom** | **X, Å** | **Y, Å** | **Z, Å** |
| C | -0.91020 | 0.66190 | 0.00021 |
| C | -1.69102 | -0.51810 | -0.00018 |
| C | 0.44501 | 0.39044 | 0.00017 |
| C | 0.90928 | -0.95007 | -0.00030 |
| N | 0.10519 | -2.00656 | -0.00076 |
| N | -1.19659 | -1.76134 | -0.00069 |
| N | 1.57464 | 1.16793 | 0.00044 |
| C | 2.63632 | 0.28353 | 0.00015 |
| N | 2.29387 | -0.97392 | -0.00030 |
| N | -3.08641 | -0.35540 | 0.00008 |
| N | -3.77380 | -1.38866 | 0.00048 |
| N | -4.51864 | -2.23383 | 0.00084 |
| N | -1.31687 | 2.00022 | 0.00113 |
| N | -2.51448 | 2.32455 | -0.00080 |
| N | -3.53092 | 2.81425 | -0.00089 |
| N | 3.91071 | 0.82675 | 0.00036 |
| N | 4.84701 | 0.00610 | 0.00008 |
| N | 5.78250 | -0.61735 | -0.00013 |
| H | 1.63028 | 2.17462 | 0.00067 |

| **IC35** | | | |
| --- | --- | --- | --- |
| **Atom** | **X, Å** | **Y, Å** | **Z, Å** |
| C | 0.35936 | -0.82298 | -0.28702 |
| C | 1.66570 | -0.36254 | -0.07862 |
| C | -0.58089 | 0.17563 | -0.17897 |
| C | -0.17647 | 1.50611 | 0.10606 |
| N | 1.08836 | 1.85699 | 0.29727 |
| N | 2.00597 | 0.91018 | 0.20324 |
| N | -1.95222 | 0.25262 | -0.27285 |
| C | -2.29257 | 1.59718 | -0.10543 |
| N | -1.27385 | 2.35759 | 0.14171 |
| H | -3.32478 | 1.90781 | -0.18043 |
| N | 2.69845 | -1.30921 | -0.17087 |
| N | 3.85569 | -0.89447 | 0.01136 |
| N | 4.95208 | -0.67585 | 0.15032 |
| N | -2.80424 | -0.74938 | -0.77428 |
| N | -3.33409 | -1.42334 | 0.15479 |
| N | -3.90374 | -2.12648 | 0.82488 |
| H | 0.14719 | -1.85876 | -0.51481 |

| **IC356** | | | |
| --- | --- | --- | --- |
| **Atom** | **X, Å** | **Y, Å** | **Z, Å** |
| C | -1.39131 | 1.01828 | -0.22288 |
| C | -2.51732 | 0.19642 | -0.05410 |
| C | -0.21044 | 0.32568 | -0.13088 |
| C | -0.22045 | -1.07615 | 0.10093 |
| N | -1.33148 | -1.77809 | 0.25696 |
| N | -2.48426 | -1.12553 | 0.17699 |
| N | 1.12697 | 0.65657 | -0.20278 |
| C | 1.82975 | -0.54698 | -0.08010 |
| N | 1.07283 | -1.58048 | 0.13072 |
| N | -3.77421 | 0.81925 | -0.13328 |
| N | -4.76962 | 0.09080 | 0.01498 |
| N | -5.76081 | -0.43294 | 0.12790 |
| N | 1.62314 | 1.86641 | -0.71757 |
| N | 2.26615 | 2.51852 | 0.15129 |
| N | 2.89364 | 3.22491 | 0.76346 |
| N | 3.20688 | -0.47356 | -0.16175 |
| N | 3.81209 | -1.55643 | -0.04970 |
| N | 4.48088 | -2.45562 | 0.03309 |
| H | -1.47678 | 2.07979 | -0.40989 |

| **IC36** | | | |
| --- | --- | --- | --- |
| **Atom** | **X, Å** | **Y, Å** | **Z, Å** |
| C | -1.18459 | 1.35553 | 0.00000 |
| C | -2.15341 | 0.33982 | -0.00000 |
| C | 0.10199 | 0.87286 | 0.00000 |
| C | 0.32620 | -0.53486 | -0.00000 |
| N | -0.64905 | -1.43022 | -0.00000 |
| N | -1.89669 | -0.97652 | -0.00000 |
| N | 1.35941 | 1.42797 | 0.00000 |
| C | 2.24532 | 0.36464 | 0.00000 |
| N | 1.68507 | -0.80963 | 0.00000 |
| N | -3.49941 | 0.74869 | -0.00000 |
| N | -4.35663 | -0.14968 | 0.00000 |
| N | -5.24437 | -0.84412 | 0.00000 |
| N | 3.59633 | 0.67334 | 0.00000 |
| N | 4.37202 | -0.30065 | 0.00000 |
| N | 5.18124 | -1.08098 | 0.00000 |
| H | -1.46253 | 2.40122 | 0.00000 |
| H | 1.61399 | 2.40335 | 0.00000 |

| **IC45** | | | |
| --- | --- | --- | --- |
| **Atom** | **X, Å** | **Y, Å** | **Z, Å** |
| C | -0.69220 | -0.71141 | -0.05839 |
| C | -0.73991 | -2.09978 | 0.12870 |
| C | 0.59810 | -0.20675 | -0.09737 |
| C | 1.68783 | -1.09475 | 0.06164 |
| N | 1.56037 | -2.40770 | 0.24998 |
| N | 0.33072 | -2.89522 | 0.27523 |
| N | 1.17746 | 1.03877 | -0.22475 |
| C | 2.55156 | 0.83634 | -0.19395 |
| N | 2.88821 | -0.40314 | -0.00768 |
| N | -1.78887 | 0.15926 | -0.19043 |
| N | -2.92937 | -0.32430 | -0.17228 |
| N | -4.01894 | -0.62220 | -0.17423 |
| N | 0.58128 | 2.23202 | -0.67530 |
| N | -0.12226 | 2.77672 | 0.21751 |
| N | -0.81586 | 3.38871 | 0.85978 |
| H | -1.69323 | -2.61679 | 0.16947 |
| H | 3.22180 | 1.67437 | -0.31798 |

| **IC456** | | | |
| --- | --- | --- | --- |
| **Atom** | **X, Å** | **Y, Å** | **Z, Å** |
| C | -1.74363 | -0.43788 | -0.05071 |
| C | -2.28538 | -1.72585 | 0.10591 |
| C | -0.36129 | -0.42268 | -0.04716 |
| C | 0.34086 | -1.63916 | 0.12459 |
| N | -0.24416 | -2.82274 | 0.28333 |
| N | -1.57146 | -2.84545 | 0.26325 |
| N | 0.61887 | 0.55079 | -0.13154 |
| C | 1.83201 | -0.13229 | -0.07029 |
| N | 1.70840 | -1.41522 | 0.10751 |
| N | -2.46179 | 0.76257 | -0.18874 |
| N | -3.69893 | 0.70760 | -0.21235 |
| N | -4.82330 | 0.81239 | -0.25022 |
| N | 0.46532 | 1.84611 | -0.66072 |
| N | 0.24480 | 2.69638 | 0.24488 |
| N | 0.02716 | 3.59103 | 0.89207 |
| N | 2.98542 | 0.61906 | -0.17776 |
| N | 4.05014 | -0.02584 | -0.13192 |
| N | 5.08032 | -0.47410 | -0.10706 |
| H | -3.36098 | -1.87084 | 0.11074 |

| **IC46** | | | |
| --- | --- | --- | --- |
| **Atom** | **X, Å** | **Y, Å** | **Z, Å** |
| C | -1.69519 | -0.05809 | 0.00003 |
| C | -2.21493 | 1.24650 | 0.00002 |
| C | -0.31416 | -0.10191 | 0.00002 |
| C | 0.41296 | 1.11498 | -0.00000 |
| N | -0.15060 | 2.32027 | -0.00001 |
| N | -1.47779 | 2.36393 | -0.00000 |
| N | 0.63182 | -1.09650 | 0.00003 |
| C | 1.85016 | -0.44863 | 0.00001 |
| N | 1.77290 | 0.85315 | -0.00001 |
| N | -2.42247 | -1.26334 | 0.00006 |
| N | -3.65929 | -1.20574 | -0.00002 |
| N | -4.78515 | -1.30402 | -0.00008 |
| N | 2.98760 | -1.24009 | 0.00000 |
| N | 4.07098 | -0.62647 | -0.00002 |
| N | 5.11363 | -0.20631 | -0.00003 |
| H | 0.48305 | -2.09366 | 0.00004 |
| H | -3.28755 | 1.41245 | 0.00003 |

| **IC56** | | | |
| --- | --- | --- | --- |
| **Atom** | **X, Å** | **Y, Å** | **Z, Å** |
| C | 2.30736 | 1.12627 | -0.21100 |
| C | 3.45762 | 0.35186 | -0.03269 |
| C | 1.14278 | 0.39838 | -0.12596 |
| C | 1.20759 | -0.99734 | 0.11100 |
| N | 2.34373 | -1.66172 | 0.27857 |
| N | 3.47679 | -0.96917 | 0.20350 |
| N | -0.20745 | 0.67786 | -0.20772 |
| C | -0.86167 | -0.54884 | -0.08328 |
| N | -0.06554 | -1.55196 | 0.13625 |
| N | -2.24103 | -0.53255 | -0.17245 |
| N | -2.80088 | -1.63905 | -0.05751 |
| N | -3.43201 | -2.56498 | 0.02690 |
| N | -0.74779 | 1.86753 | -0.72609 |
| N | -1.42527 | 2.49159 | 0.13645 |
| N | -2.08705 | 3.17063 | 0.74389 |
| H | 4.43931 | 0.80976 | -0.07871 |
| H | 2.34420 | 2.19103 | -0.40224 |

| **ID34** | | | |
| --- | --- | --- | --- |
| **Atom** | **X, Å** | **Y, Å** | **Z, Å** |
| C | -0.17820 | 0.08339 | -0.53163 |
| C | 0.34914 | -1.20935 | -0.36478 |
| C | -1.53485 | 0.16243 | -0.26835 |
| C | -2.21885 | -1.02113 | 0.12959 |
| N | -1.63646 | -2.20725 | 0.27562 |
| N | -0.34076 | -2.28089 | 0.01958 |
| N | -2.49135 | 1.13679 | -0.28447 |
| C | -3.67611 | 0.52368 | 0.09435 |
| N | -3.55712 | -0.74516 | 0.34498 |
| H | -2.35998 | 2.11307 | -0.49696 |
| H | -4.59445 | 1.08928 | 0.16034 |
| O | 1.65330 | -1.46908 | -0.71328 |
| O | 0.60186 | 1.10330 | -0.98525 |
| N | 2.66848 | -0.95020 | 0.25864 |
| N | 0.89111 | 2.20786 | 0.06950 |
| O | 3.76484 | -1.27722 | -0.04932 |
| O | 2.24965 | -0.29165 | 1.16822 |
| O | 0.19215 | 2.19809 | 1.03350 |
| O | 1.75949 | 2.91599 | -0.30455 |

| **ID345** | | | |
| --- | --- | --- | --- |
| **Atom** | **X, Å** | **Y, Å** | **Z, Å** |
| C | -0.59117 | 0.32295 | -0.57616 |
| C | -1.74171 | 1.07707 | -0.27203 |
| C | 0.58654 | 1.01786 | -0.36167 |
| C | 0.51177 | 2.36544 | 0.07871 |
| N | -0.62142 | 3.00120 | 0.34912 |
| N | -1.74826 | 2.33028 | 0.17553 |
| N | 1.93746 | 0.79916 | -0.53900 |
| C | 2.59075 | 1.98995 | -0.19841 |
| N | 1.78035 | 2.92675 | 0.16739 |
| H | 3.66754 | 2.04945 | -0.26290 |
| O | -2.98604 | 0.56934 | -0.54808 |
| O | -0.70818 | -0.91421 | -1.12739 |
| N | -3.45467 | -0.49013 | 0.41086 |
| N | -0.25320 | -2.08246 | -0.22471 |
| O | -4.56491 | -0.82234 | 0.17037 |
| O | -2.66579 | -0.84218 | 1.24018 |
| O | 0.33483 | -1.77556 | 0.76524 |
| O | -0.56244 | -3.11531 | -0.70354 |
| O | 2.52929 | -0.32763 | -0.91953 |
| N | 3.08947 | -1.16147 | 0.41267 |
| O | 3.00989 | -0.55529 | 1.42280 |
| O | 3.49925 | -2.21087 | 0.07210 |

| **ID3456** | | | |
| --- | --- | --- | --- |
| **Atom** | **X, Å** | **Y, Å** | **Z, Å** |
| C | 1.23555 | -0.39859 | -0.46906 |
| C | 2.09591 | -1.47191 | -0.16698 |
| C | -0.06688 | -0.61121 | -0.04833 |
| C | -0.39099 | -1.84733 | 0.57558 |
| N | 0.49222 | -2.80911 | 0.82718 |
| N | 1.74153 | -2.59602 | 0.45491 |
| N | -1.27169 | 0.04762 | -0.13372 |
| C | -2.23902 | -0.79939 | 0.45691 |
| N | -1.73873 | -1.92196 | 0.86956 |
| O | 1.68089 | 0.65285 | -1.20439 |
| O | -1.50645 | 1.26349 | -0.60468 |
| N | 1.76136 | 2.00108 | -0.44689 |
| N | -1.61156 | 2.35303 | 0.66218 |
| O | 2.32348 | 2.80468 | -1.10198 |
| O | 1.24598 | 2.02384 | 0.62659 |
| O | -1.53645 | 1.84963 | 1.72554 |
| O | -1.77247 | 3.43479 | 0.22926 |
| O | -3.48346 | -0.37848 | 0.55811 |
| N | -4.42869 | -0.90377 | -0.66610 |
| O | -5.52671 | -0.52827 | -0.49199 |
| O | -3.85986 | -1.54262 | -1.47968 |
| O | 3.38679 | -1.46687 | -0.62677 |
| N | 4.30938 | -0.52530 | 0.10397 |
| O | 3.80508 | 0.17108 | 0.93688 |
| O | 5.42016 | -0.62766 | -0.29020 |

| **ID346** | | | |
| --- | --- | --- | --- |
| **Atom** | **X, Å** | **Y, Å** | **Z, Å** |
| C | -0.94168 | 0.00400 | 0.46119 |
| C | -1.63647 | -1.21111 | 0.32162 |
| C | 0.39040 | -0.08004 | 0.09250 |
| C | 0.88764 | -1.32925 | -0.38448 |
| N | 0.15090 | -2.43126 | -0.49652 |
| N | -1.11743 | -2.35038 | -0.13317 |
| N | 1.45879 | 0.76595 | 0.05198 |
| C | 2.51547 | 0.02303 | -0.46657 |
| N | 2.21735 | -1.21621 | -0.72570 |
| O | -2.92872 | -1.31839 | 0.77338 |
| O | -1.54787 | 1.10101 | 0.98797 |
| N | -3.94748 | -0.64309 | -0.09754 |
| N | -1.77814 | 2.25941 | -0.03290 |
| O | -5.04612 | -0.83433 | 0.30064 |
| O | -3.52334 | -0.02010 | -1.02898 |
| O | -1.16065 | 2.18126 | -1.04724 |
| O | -2.52078 | 3.05908 | 0.41676 |
| O | 3.68337 | 0.62418 | -0.67063 |
| N | 4.84372 | 0.11917 | 0.32660 |
| O | 5.87441 | 0.56429 | -0.02329 |
| O | 4.47384 | -0.57187 | 1.21050 |
| H | 1.48097 | 1.75409 | 0.25215 |

| **ID35** | | | |
| --- | --- | --- | --- |
| **Atom** | **X, Å** | **Y, Å** | **Z, Å** |
| C | -0.43118 | -0.28271 | -0.77844 |
| C | -1.68264 | 0.27743 | -0.51668 |
| C | 0.60101 | 0.55337 | -0.41865 |
| C | 0.31995 | 1.81404 | 0.15834 |
| N | -0.91061 | 2.26051 | 0.38422 |
| N | -1.91381 | 1.47282 | 0.03289 |
| N | 1.98059 | 0.51258 | -0.47750 |
| C | 2.44853 | 1.71332 | 0.07118 |
| N | 1.49589 | 2.50304 | 0.44490 |
| H | 3.51094 | 1.90063 | 0.13243 |
| O | -2.79753 | -0.42043 | -0.91627 |
| O | 2.72866 | -0.47792 | -0.94599 |
| N | -3.62412 | -0.96004 | 0.23255 |
| N | 3.09565 | -1.54219 | 0.27376 |
| O | -4.66855 | -1.36667 | -0.15454 |
| O | -3.11468 | -0.92693 | 1.30998 |
| O | 2.67637 | -1.22039 | 1.33256 |
| O | 3.72687 | -2.44018 | -0.15517 |
| H | -0.31916 | -1.26032 | -1.22725 |

| **ID356** | | | |
| --- | --- | --- | --- |
| **Atom** | **X, Å** | **Y, Å** | **Z, Å** |
| C | 1.41637 | 0.33353 | -0.77437 |
| C | 2.48511 | -0.51096 | -0.47321 |
| C | 0.22659 | -0.12085 | -0.25116 |
| C | 0.19984 | -1.31333 | 0.51414 |
| N | 1.28072 | -2.04957 | 0.76243 |
| N | 2.42542 | -1.63432 | 0.25166 |
| N | -1.08653 | 0.29502 | -0.27073 |
| C | -1.81760 | -0.63328 | 0.50694 |
| N | -1.07906 | -1.59895 | 0.95898 |
| O | 3.70971 | -0.21880 | -1.01916 |
| O | -1.58216 | 1.37160 | -0.85967 |
| N | 4.75861 | 0.18209 | 0.00503 |
| N | -1.60141 | 2.64516 | 0.20884 |
| O | 5.83650 | 0.20589 | -0.48665 |
| O | 4.35642 | 0.43404 | 1.09789 |
| O | -1.17256 | 2.37545 | 1.27686 |
| O | -2.05377 | 3.59729 | -0.31589 |
| O | -3.10362 | -0.42832 | 0.71646 |
| N | -4.04895 | -1.21880 | -0.34748 |
| O | -5.17653 | -1.02529 | -0.08278 |
| O | -3.45660 | -1.82493 | -1.17050 |
| H | 1.53735 | 1.22952 | -1.36765 |

| **ID36** | | | |
| --- | --- | --- | --- |
| **Atom** | **X, Å** | **Y, Å** | **Z, Å** |
| C | 1.17805 | -1.05379 | 0.37275 |
| C | 2.09551 | -0.01109 | 0.47825 |
| C | -0.08794 | -0.61951 | 0.03324 |
| C | -0.30475 | 0.76859 | -0.19945 |
| N | 0.64738 | 1.69455 | -0.09273 |
| N | 1.85196 | 1.28562 | 0.25904 |
| N | -1.30888 | -1.20316 | -0.17208 |
| C | -2.16893 | -0.17376 | -0.53612 |
| N | -1.61578 | 1.00328 | -0.55436 |
| O | 3.36642 | -0.31022 | 0.91396 |
| O | -3.43004 | -0.46617 | -0.85415 |
| N | 4.42238 | -0.18354 | -0.15946 |
| N | -4.48012 | -0.01930 | 0.26788 |
| O | 5.51097 | -0.25543 | 0.30703 |
| O | 4.02752 | -0.06352 | -1.27867 |
| O | -3.99984 | 0.35515 | 1.28233 |
| O | -5.57549 | -0.16496 | -0.13862 |
| H | 1.46209 | -2.08108 | 0.55931 |
| H | -1.54860 | -2.18250 | -0.14438 |

| **ID45** | | | |
| --- | --- | --- | --- |
| **Atom** | **X, Å** | **Y, Å** | **Z, Å** |
| C | 0.79135 | 0.96182 | -0.51372 |
| C | 2.06050 | 1.38108 | -0.92245 |
| C | 0.77078 | -0.28819 | 0.07495 |
| C | 1.98954 | -0.99498 | 0.20147 |
| N | 3.16328 | -0.53368 | -0.21055 |
| N | 3.18517 | 0.66362 | -0.78457 |
| N | -0.16964 | -1.12175 | 0.63708 |
| C | 0.50651 | -2.27889 | 1.04803 |
| N | 1.77583 | -2.22969 | 0.81524 |
| O | -0.32154 | 1.71484 | -0.77110 |
| O | -1.47120 | -0.88862 | 0.76031 |
| N | -0.92286 | 2.38562 | 0.48116 |
| N | -2.27271 | -1.39743 | -0.60236 |
| O | -1.87870 | 3.01981 | 0.19181 |
| O | -0.35809 | 2.17042 | 1.50679 |
| O | -3.42016 | -1.15060 | -0.50486 |
| O | -1.57029 | -1.90509 | -1.40731 |
| H | 2.17445 | 2.34470 | -1.40608 |
| H | -0.04021 | -3.08282 | 1.51931 |

| **ID456** | | | |
| --- | --- | --- | --- |
| **Atom** | **X, Å** | **Y, Å** | **Z, Å** |
| C | -1.87750 | 1.01367 | -0.44727 |
| C | -2.45824 | 2.25090 | -0.16146 |
| C | -0.55458 | 0.89792 | -0.05606 |
| C | 0.06690 | 2.03213 | 0.53022 |
| N | -0.54762 | 3.18902 | 0.75674 |
| N | -1.82221 | 3.28527 | 0.41025 |
| N | 0.44853 | -0.04070 | -0.12803 |
| C | 1.59997 | 0.55653 | 0.43609 |
| N | 1.39890 | 1.77874 | 0.81370 |
| O | -2.58116 | 0.08921 | -1.17824 |
| O | 0.36149 | -1.29461 | -0.54702 |
| N | -2.97769 | -1.16379 | -0.42731 |
| N | 0.16827 | -2.31809 | 0.76221 |
| O | -3.65523 | -1.86366 | -1.09867 |
| O | -2.55779 | -1.26459 | 0.68856 |
| O | 0.21672 | -1.76762 | 1.80415 |
| O | 0.04413 | -3.42316 | 0.37744 |
| O | 2.70362 | -0.15935 | 0.55391 |
| N | 3.71146 | 0.03302 | -0.70228 |
| O | 4.68342 | -0.60142 | -0.52173 |
| O | 3.30670 | 0.75027 | -1.54974 |
| H | -3.49206 | 2.42812 | -0.43523 |

| **ID46** | | | |
| --- | --- | --- | --- |
| **Atom** | **X, Å** | **Y, Å** | **Z, Å** |
| C | 1.73299 | 0.33601 | 0.48953 |
| C | 2.26269 | 1.62662 | 0.47980 |
| C | 0.40994 | 0.25271 | 0.09209 |
| C | -0.25182 | 1.45617 | -0.28254 |
| N | 0.32220 | 2.65606 | -0.27276 |
| N | 1.58747 | 2.72929 | 0.11628 |
| N | -0.52346 | -0.73141 | -0.04778 |
| C | -1.67191 | -0.10038 | -0.51688 |
| N | -1.55171 | 1.18568 | -0.66193 |
| O | 2.43588 | -0.75465 | 0.93373 |
| O | -2.74505 | -0.83896 | -0.79878 |
| N | 3.35365 | -1.38056 | -0.13618 |
| N | -3.92913 | -0.65892 | 0.26201 |
| O | 3.93029 | -2.31526 | 0.30797 |
| O | 3.34546 | -0.85464 | -1.20444 |
| O | -3.64603 | -0.03726 | 1.22798 |
| O | -4.89287 | -1.21145 | -0.12801 |
| H | -0.41555 | -1.72079 | 0.11576 |
| H | 3.28956 | 1.79088 | 0.78706 |

| **ID56** | | | |
| --- | --- | --- | --- |
| **Atom** | **X, Å** | **Y, Å** | **Z, Å** |
| C | 2.47303 | 0.71749 | -0.86695 |
| C | 3.66601 | 0.04491 | -0.60142 |
| C | 1.37147 | 0.10734 | -0.30763 |
| C | 1.53440 | -1.08218 | 0.44089 |
| N | 2.71135 | -1.66632 | 0.64586 |
| N | 3.78026 | -1.08809 | 0.11734 |
| N | 0.01130 | 0.33722 | -0.28265 |
| C | -0.55831 | -0.69019 | 0.50213 |
| N | 0.31800 | -1.54730 | 0.92066 |
| O | -1.85710 | -0.66856 | 0.75253 |
| O | -0.64857 | 1.33808 | -0.84301 |
| N | -2.71121 | -1.54052 | -0.30493 |
| N | -0.80587 | 2.58937 | 0.23714 |
| O | -3.84910 | -1.51059 | -0.01047 |
| O | -2.06999 | -2.03968 | -1.16421 |
| O | -1.39690 | 3.47825 | -0.26266 |
| O | -0.31395 | 2.37494 | 1.29113 |
| H | 4.60725 | 0.42251 | -0.98425 |
| H | 2.43115 | 1.62312 | -1.45826 |

| **TA35** | | | |
| --- | --- | --- | --- |
| **Atom** | **X, Å** | **Y, Å** | **Z, Å** |
| C | -2.48651 | -1.42038 | 0.02910 |
| N | -3.05442 | -0.25056 | 0.16302 |
| C | -1.08090 | -1.57632 | -0.06514 |
| N | -0.27894 | -0.42957 | -0.00558 |
| C | -0.89535 | 0.80461 | 0.04153 |
| C | -2.25352 | 0.85502 | 0.14608 |
| N | -0.32247 | -2.66174 | -0.14476 |
| C | 0.98765 | -0.94670 | -0.04262 |
| N | 0.96020 | -2.25825 | -0.12950 |
| H | -2.73616 | 1.82246 | 0.18479 |
| H | -3.10826 | -2.30910 | 0.00132 |
| N | -0.12923 | 2.02173 | -0.25178 |
| N | 2.20378 | -0.20156 | 0.25103 |
| O | -0.54085 | 3.05802 | 0.23516 |
| O | 0.80855 | 1.89454 | -1.02268 |
| O | 2.07602 | 0.71047 | 1.05929 |
| O | 3.22676 | -0.58191 | -0.27388 |

| **TA356** | | | |
| --- | --- | --- | --- |
| **Atom** | **X, Å** | **Y, Å** | **Z, Å** |
| C | 0.70968 | 2.59763 | 0.05096 |
| N | 1.79773 | 1.88110 | -0.03617 |
| C | -0.57295 | 1.99981 | 0.08329 |
| N | -0.65377 | 0.59917 | 0.00920 |
| C | 0.50407 | -0.14937 | 0.01111 |
| C | 1.68049 | 0.53896 | -0.02964 |
| N | -1.78632 | 2.52784 | 0.12902 |
| C | -2.00891 | 0.38178 | 0.01076 |
| N | -2.66866 | 1.51455 | 0.08472 |
| N | -2.65243 | -0.89507 | -0.26520 |
| O | -2.01329 | -1.65662 | -0.98287 |
| O | -3.76223 | -1.06362 | 0.18580 |
| N | 2.99069 | -0.18057 | -0.01260 |
| O | 3.78437 | 0.15202 | -0.86427 |
| O | 3.14206 | -1.00396 | 0.87199 |
| H | 0.79029 | 3.67816 | 0.09015 |
| N | 0.40975 | -1.61402 | 0.20335 |
| O | 1.02601 | -2.31000 | -0.56998 |
| O | -0.26735 | -1.95808 | 1.15491 |

| **TA3568** | | | |
| --- | --- | --- | --- |
| **Atom** | **X, Å** | **Y, Å** | **Z, Å** |
| C | 1.90687 | -0.63996 | 0.01013 |
| C | 0.69329 | -1.37103 | 0.04859 |
| N | -0.49147 | -0.61109 | -0.00558 |
| C | -0.43322 | 0.76706 | -0.00906 |
| C | 0.80557 | 1.33488 | -0.05365 |
| N | 1.96258 | 0.64531 | -0.05847 |
| N | 0.41684 | -2.65845 | 0.12388 |
| C | -1.45891 | -1.58611 | 0.03596 |
| N | -0.92425 | -2.77981 | 0.11692 |
| N | -1.68920 | 1.53649 | 0.17142 |
| N | 3.20325 | -1.38052 | 0.02854 |
| O | -1.93414 | 2.38521 | -0.65166 |
| O | -2.32642 | 1.22799 | 1.16067 |
| O | 3.34867 | -2.18494 | -0.87077 |
| O | 3.97382 | -1.09478 | 0.91869 |
| N | -2.88276 | -1.36794 | -0.19865 |
| O | -3.64959 | -2.15613 | 0.30224 |
| O | -3.14784 | -0.42492 | -0.93488 |
| N | 0.97944 | 2.81975 | -0.04488 |
| O | 1.78309 | 3.25853 | -0.83568 |
| O | 0.31459 | 3.43212 | 0.77089 |

| **TA358** | | | |
| --- | --- | --- | --- |
| **Atom** | **X, Å** | **Y, Å** | **Z, Å** |
| C | 2.03705 | 0.31964 | -0.03775 |
| C | 0.99750 | -0.64142 | 0.05350 |
| N | -0.31308 | -0.13975 | 0.00037 |
| C | -0.51673 | 1.22549 | -0.01192 |
| C | 0.56260 | 2.04997 | -0.11225 |
| N | 1.84655 | 1.58939 | -0.15652 |
| N | 0.98593 | -1.96290 | 0.08447 |
| C | -1.06472 | -1.28380 | -0.00492 |
| N | -0.30252 | -2.35131 | 0.04759 |
| N | -1.84214 | 1.77710 | 0.30748 |
| N | 3.45310 | -0.15257 | 0.01005 |
| O | -2.10522 | 2.86634 | -0.16264 |
| O | -2.52147 | 1.12085 | 1.07885 |
| O | 4.16151 | 0.16224 | -0.92155 |
| O | 3.74990 | -0.79335 | 1.00033 |
| H | 0.41367 | 3.12082 | -0.12734 |
| N | -2.49196 | -1.34303 | -0.30129 |
| O | -3.12108 | -2.25015 | 0.19295 |
| O | -2.89601 | -0.48825 | -1.08014 |

| **TA36** | | | |
| --- | --- | --- | --- |
| **Atom** | **X, Å** | **Y, Å** | **Z, Å** |
| C | 0.90681 | 2.17865 | 0.00002 |
| N | 1.95508 | 1.39906 | 0.00006 |
| C | -0.40941 | 1.65179 | 0.00002 |
| N | -0.55359 | 0.26310 | 0.00005 |
| C | 0.54289 | -0.56579 | 0.00006 |
| C | 1.74965 | 0.06842 | 0.00008 |
| N | -1.60225 | 2.23968 | -0.00002 |
| C | -1.91795 | 0.10274 | 0.00003 |
| N | -2.52944 | 1.26641 | -0.00003 |
| H | 1.04688 | 3.25398 | -0.00001 |
| N | 2.97908 | -0.78760 | -0.00002 |
| O | 2.78993 | -1.99678 | 0.00001 |
| O | 4.05048 | -0.22340 | -0.00015 |
| H | 0.39232 | -1.63177 | 0.00006 |
| N | -2.58112 | -1.18354 | -0.00002 |
| O | -3.79264 | -1.18915 | -0.00016 |
| O | -1.84095 | -2.16777 | 0.00012 |

| **TA368** | | | |
| --- | --- | --- | --- |
| **Atom** | **X, Å** | **Y, Å** | **Z, Å** |
| C | 1.33090 | -0.93097 | -0.01231 |
| C | -0.07112 | -1.15843 | -0.00615 |
| N | -0.86943 | -0.00756 | 0.00126 |
| C | -0.32542 | 1.25462 | 0.02413 |
| C | 1.03603 | 1.30592 | 0.02089 |
| N | 1.86193 | 0.24291 | -0.00883 |
| N | -0.82469 | -2.24745 | -0.04301 |
| C | -2.13941 | -0.53133 | -0.02830 |
| N | -2.10892 | -1.84245 | -0.05709 |
| N | -3.34809 | 0.27284 | -0.03172 |
| O | -4.40585 | -0.31341 | -0.05991 |
| O | -3.17801 | 1.49119 | -0.00518 |
| N | 2.25951 | -2.10175 | -0.00492 |
| O | 2.04625 | -2.92371 | 0.86660 |
| O | 3.13185 | -2.10863 | -0.84595 |
| H | -0.98425 | 2.10637 | 0.03884 |
| N | 1.68937 | 2.65184 | 0.05015 |
| O | 0.92982 | 3.61013 | 0.04013 |
| O | 2.89852 | 2.67896 | 0.08315 |

| **TA38** | | | |
| --- | --- | --- | --- |
| **Atom** | **X, Å** | **Y, Å** | **Z, Å** |
| C | -1.71372 | 0.29646 | 0.01465 |
| N | -1.82607 | 1.57944 | 0.06144 |
| C | -0.47274 | -0.39115 | -0.00587 |
| N | 0.66339 | 0.41783 | 0.02803 |
| C | 0.56963 | 1.79281 | 0.10007 |
| C | -0.68327 | 2.32744 | 0.11597 |
| N | -0.13074 | -1.67391 | -0.09201 |
| C | 1.68158 | -0.50025 | -0.03844 |
| N | 1.21041 | -1.72592 | -0.11167 |
| H | 1.49276 | 2.34704 | 0.13376 |
| H | -0.81414 | 3.39987 | 0.17006 |
| N | 3.08602 | -0.15381 | -0.03261 |
| N | -2.98145 | -0.49595 | -0.00145 |
| O | -3.04398 | -1.39879 | 0.81317 |
| O | -3.82724 | -0.15705 | -0.80254 |
| O | 3.34407 | 1.04937 | 0.03687 |
| O | 3.88735 | -1.06010 | -0.09552 |

| **TA56** | | | |
| --- | --- | --- | --- |
| **Atom** | **X, Å** | **Y, Å** | **Z, Å** |
| C | -0.89735 | -2.14719 | 0.00003 |
| N | 0.40166 | -1.97499 | 0.00003 |
| C | -1.79794 | -1.06270 | -0.00000 |
| N | -1.27479 | 0.24437 | -0.00003 |
| C | 0.09982 | 0.41909 | -0.00007 |
| C | 0.87020 | -0.71277 | -0.00001 |
| N | -3.11828 | -1.02094 | 0.00001 |
| C | -2.39288 | 1.03696 | -0.00004 |
| N | -3.47570 | 0.27860 | -0.00001 |
| H | -1.28859 | -3.15853 | 0.00006 |
| N | 2.36279 | -0.67436 | 0.00004 |
| O | 2.89081 | -0.70178 | 1.09140 |
| O | 2.89095 | -0.70216 | -1.09124 |
| N | 0.63650 | 1.77682 | -0.00005 |
| O | -0.17578 | 2.69279 | 0.00017 |
| O | 1.84919 | 1.89143 | -0.00025 |
| H | -2.36918 | 2.10946 | -0.00006 |

| **TA568** | | | |
| --- | --- | --- | --- |
| **Atom** | **X, Å** | **Y, Å** | **Z, Å** |
| C | -1.47994 | -0.56108 | -0.00441 |
| C | -1.35808 | 0.84311 | -0.01415 |
| N | -0.04752 | 1.36108 | -0.02279 |
| C | 1.03358 | 0.49311 | -0.00157 |
| C | 0.76079 | -0.84764 | 0.00822 |
| N | -0.47773 | -1.37854 | -0.00147 |
| N | -2.23905 | 1.82122 | -0.04435 |
| C | -0.24846 | 2.71756 | -0.05664 |
| N | -1.54358 | 2.97645 | -0.07034 |
| H | 0.54380 | 3.44107 | -0.06979 |
| N | 1.82082 | -1.89917 | 0.02822 |
| N | 2.38628 | 1.05051 | 0.00828 |
| N | -2.84164 | -1.16568 | 0.01045 |
| O | 2.14124 | -2.29810 | 1.12681 |
| O | 2.18880 | -2.29387 | -1.05679 |
| O | 3.30622 | 0.25477 | 0.04310 |
| O | 2.48081 | 2.26946 | -0.01875 |
| O | -3.46302 | -1.01624 | 1.04309 |
| O | -3.17833 | -1.75007 | -0.99683 |

| **TA58** | | | |
| --- | --- | --- | --- |
| **Atom** | **X, Å** | **Y, Å** | **Z, Å** |
| C | 1.35371 | -0.52530 | 0.00925 |
| N | 0.75939 | -1.67290 | 0.03113 |
| C | 0.69704 | 0.72602 | -0.01792 |
| N | -0.70963 | 0.69579 | -0.02358 |
| C | -1.34811 | -0.52965 | 0.01635 |
| C | -0.60435 | -1.67277 | 0.04395 |
| N | 1.12821 | 1.96995 | -0.06948 |
| C | -1.05466 | 2.02160 | -0.07852 |
| N | 0.03577 | 2.76459 | -0.10644 |
| H | -1.10582 | -2.62936 | 0.07487 |
| N | -2.81002 | -0.57697 | 0.02621 |
| O | -3.32883 | -1.67926 | 0.06297 |
| O | -3.39950 | 0.49599 | -0.00309 |
| H | -2.06780 | 2.37548 | -0.09500 |
| N | 2.84469 | -0.54246 | 0.02164 |
| O | 3.36307 | -0.13065 | 1.04097 |
| O | 3.38689 | -0.97754 | -0.97271 |

| **TA68** | | | |
| --- | --- | --- | --- |
| **Atom** | **X, Å** | **Y, Å** | **Z, Å** |
| C | -0.65876 | -0.76054 | -0.00888 |
| N | 0.61028 | -0.97936 | -0.00067 |
| C | -1.24307 | 0.53673 | -0.02056 |
| N | -0.32487 | 1.60120 | -0.02549 |
| C | 1.02818 | 1.39198 | 0.00208 |
| C | 1.43179 | 0.09252 | 0.01611 |
| N | -2.47309 | 0.99928 | -0.06496 |
| N | -2.38611 | 2.35871 | -0.09644 |
| C | -1.12199 | 2.71414 | -0.07073 |
| H | -0.74362 | 3.72313 | -0.08493 |
| H | 1.70360 | 2.23340 | 0.00842 |
| N | -1.55170 | -1.95799 | 0.00972 |
| N | 2.89479 | -0.19009 | 0.05017 |
| O | -2.38213 | -1.97266 | 0.89935 |
| O | -1.35624 | -2.79802 | -0.84303 |
| O | 3.62833 | 0.79190 | 0.03874 |
| O | 3.23979 | -1.34970 | 0.08769 |

| **TB35** | | | |
| --- | --- | --- | --- |
| **Atom** | **X, Å** | **Y, Å** | **Z, Å** |
| C | -3.22511 | -0.00734 | 0.36267 |
| C | -2.17723 | -0.93032 | 0.11741 |
| N | -0.93527 | -0.41932 | -0.25373 |
| C | -0.75961 | 0.94619 | -0.43249 |
| C | -1.84526 | 1.74497 | -0.20088 |
| N | -3.05972 | 1.28034 | 0.21565 |
| N | -2.12365 | -2.25648 | 0.18609 |
| C | -0.14921 | -1.54078 | -0.36978 |
| N | -0.86105 | -2.62055 | -0.11059 |
| N | 1.26568 | 2.27938 | -0.04489 |
| O | 2.45230 | 2.26137 | -0.29351 |
| O | 0.66992 | 2.97310 | 0.74427 |
| N | 2.16865 | -1.39458 | 0.32412 |
| O | 1.81651 | -0.72691 | 1.26789 |
| O | 3.23318 | -1.91809 | 0.11060 |
| H | -4.19217 | -0.38446 | 0.67760 |
| N | 1.18987 | -1.52161 | -0.76891 |
| N | 0.48793 | 1.39718 | -0.86990 |
| H | 1.11325 | 0.71344 | -1.28287 |
| H | -1.74960 | 2.81281 | -0.33684 |
| H | 1.46466 | -2.34436 | -1.29841 |

| **TB356** | | | |
| --- | --- | --- | --- |
| **Atom** | **X, Å** | **Y, Å** | **Z, Å** |
| C | 0.86392 | 2.88657 | 0.59186 |
| C | -0.38571 | 2.41298 | 0.13121 |
| N | -0.49833 | 1.05649 | -0.17824 |
| C | 0.58115 | 0.19973 | -0.02194 |
| C | 1.75672 | 0.77998 | 0.38779 |
| N | 1.90120 | 2.09944 | 0.69935 |
| N | -1.53162 | 3.03354 | -0.10488 |
| C | -1.79660 | 0.93695 | -0.63038 |
| N | -2.39009 | 2.11875 | -0.58881 |
| N | -0.07478 | -2.02391 | 0.68825 |
| O | 0.19680 | -3.19044 | 0.53861 |
| O | -0.73523 | -1.49908 | 1.55826 |
| N | 3.62826 | -0.56631 | -0.48200 |
| O | 4.81824 | -0.70492 | -0.32281 |
| O | 2.96157 | -0.92809 | -1.44265 |
| H | 0.96818 | 3.93313 | 0.85595 |
| N | 2.93118 | 0.01400 | 0.60591 |
| H | 3.61064 | 0.50033 | 1.18312 |
| N | 0.41041 | -1.15021 | -0.35694 |
| H | 1.20678 | -1.58251 | -0.82276 |
| N | -2.39659 | -0.23195 | -1.06147 |
| N | -3.31762 | -0.91346 | -0.18496 |
| H | -1.85211 | -0.91859 | -1.56622 |
| O | -3.78988 | -0.27668 | 0.72153 |
| O | -3.53706 | -2.06133 | -0.50980 |

| **TB3568** | | | |
| --- | --- | --- | --- |
| **Atom** | **X, Å** | **Y, Å** | **Z, Å** |
| C | -2.17509 | 0.33092 | -0.14628 |
| C | -1.40270 | -0.78832 | -0.58063 |
| N | -0.01719 | -0.65572 | -0.51929 |
| C | 0.59117 | 0.54073 | -0.15043 |
| C | -0.25936 | 1.59115 | 0.08695 |
| N | -1.61292 | 1.47446 | 0.14022 |
| N | -1.73365 | -1.96689 | -1.08223 |
| C | 0.43437 | -1.86450 | -0.98851 |
| N | -0.58640 | -2.63017 | -1.32381 |
| N | 2.57178 | 0.60293 | 1.26460 |
| O | 3.76000 | 0.37427 | 1.25318 |
| O | 1.85383 | 0.85561 | 2.20139 |
| N | 2.44226 | -2.79127 | -0.00184 |
| O | 2.04288 | -2.42498 | 1.07854 |
| O | 3.35207 | -3.53391 | -0.27123 |
| N | 1.78079 | -2.18627 | -1.16521 |
| N | 1.97080 | 0.62877 | -0.04532 |
| H | 2.56218 | 0.13538 | -0.70211 |
| H | 1.94386 | -2.81433 | -1.94730 |
| N | -3.56961 | 0.28877 | -0.15567 |
| N | -4.28773 | -0.66269 | 0.64719 |
| H | -4.02574 | 1.19136 | -0.08012 |
| O | -3.66277 | -1.58356 | 1.11339 |
| O | -5.47012 | -0.41875 | 0.74502 |
| N | 0.26486 | 2.86972 | 0.35494 |
| N | 0.77399 | 3.63201 | -0.74069 |
| H | -0.32176 | 3.47377 | 0.91900 |
| O | 0.83294 | 4.82568 | -0.53605 |
| O | 1.13270 | 3.02168 | -1.72505 |

| **TB358** | | | |
| --- | --- | --- | --- |
| **Atom** | **X, Å** | **Y, Å** | **Z, Å** |
| C | -2.13659 | 0.67371 | -0.44772 |
| C | -1.26000 | -0.44743 | -0.56151 |
| N | 0.10582 | -0.17312 | -0.59514 |
| C | 0.58065 | 1.13354 | -0.64216 |
| C | -0.35669 | 2.12712 | -0.61992 |
| N | -1.69257 | 1.90047 | -0.48712 |
| N | -1.47050 | -1.75093 | -0.66540 |
| C | 0.67538 | -1.42036 | -0.68576 |
| N | -0.26253 | -2.34502 | -0.73192 |
| N | 2.66637 | 2.01250 | 0.30434 |
| O | 3.85208 | 1.76473 | 0.32853 |
| O | 2.03848 | 2.77805 | 0.99545 |
| N | 2.75886 | -1.78482 | 0.50177 |
| O | 2.33311 | -1.10795 | 1.40716 |
| O | 3.72316 | -2.50621 | 0.46984 |
| N | 2.05041 | -1.64760 | -0.78522 |
| N | 1.95511 | 1.34187 | -0.75435 |
| H | 2.52109 | 0.56280 | -1.07374 |
| H | -0.03736 | 3.15760 | -0.67834 |
| H | 2.27857 | -2.47991 | -1.32232 |
| N | -3.52641 | 0.49512 | -0.42797 |
| N | -4.14976 | -0.20195 | 0.65797 |
| H | -4.05509 | 1.34101 | -0.61260 |
| O | -3.44241 | -0.87460 | 1.37004 |
| O | -5.35033 | -0.04235 | 0.71781 |

| **TB36** | | | |
| --- | --- | --- | --- |
| **Atom** | **X, Å** | **Y, Å** | **Z, Å** |
| C | 0.62255 | 2.69257 | 0.31078 |
| C | -0.62554 | 2.09978 | 0.00053 |
| N | -0.64170 | 0.72139 | -0.17572 |
| C | 0.49261 | -0.05214 | -0.06208 |
| C | 1.64634 | 0.62968 | 0.20864 |
| N | 1.71443 | 1.98527 | 0.40376 |
| N | -1.84432 | 2.60128 | -0.16674 |
| C | -1.95623 | 0.44619 | -0.42724 |
| N | -2.66578 | 1.56238 | -0.42298 |
| N | -2.63621 | -1.75681 | 0.31094 |
| O | -3.45416 | -2.61990 | 0.09116 |
| O | -1.91694 | -1.62280 | 1.27906 |
| N | 3.33895 | -1.14619 | -0.19046 |
| O | 4.54536 | -1.31296 | -0.18944 |
| O | 2.48523 | -1.91628 | -0.59921 |
| H | 0.67811 | 3.76383 | 0.47088 |
| N | 2.91149 | 0.04235 | 0.38398 |
| H | 3.67829 | 0.68621 | 0.53419 |
| N | -2.44006 | -0.81592 | -0.76035 |
| H | 0.40830 | -1.11631 | -0.18393 |
| H | -3.27653 | -0.81101 | -1.33443 |

| **TB368** | | | |
| --- | --- | --- | --- |
| **Atom** | **X, Å** | **Y, Å** | **Z, Å** |
| C | -1.60551 | -0.69224 | 0.01594 |
| C | -0.37206 | -1.38075 | -0.22048 |
| N | 0.77072 | -0.61874 | -0.28102 |
| C | 0.76004 | 0.75793 | -0.13669 |
| C | -0.47564 | 1.30919 | 0.05295 |
| N | -1.64449 | 0.59948 | 0.14053 |
| N | -0.10390 | -2.66345 | -0.40032 |
| C | 1.75622 | -1.55010 | -0.48409 |
| N | 1.23672 | -2.76269 | -0.55514 |
| N | 3.89089 | -0.91251 | 0.45981 |
| O | 3.29533 | -0.44714 | 1.40804 |
| O | 5.07863 | -1.08928 | 0.32366 |
| N | -4.06229 | -1.18079 | 0.19325 |
| O | -4.80726 | -2.14330 | 0.16763 |
| O | -4.34846 | -0.01190 | 0.29328 |
| N | -2.70128 | -1.52945 | 0.09906 |
| N | 3.09249 | -1.22838 | -0.69852 |
| H | -2.56704 | -2.52597 | -0.03096 |
| H | 1.69085 | 1.29127 | -0.17473 |
| H | 3.60604 | -1.89492 | -1.26528 |
| N | -0.73463 | 2.67593 | 0.24003 |
| N | 0.07291 | 3.72622 | -0.16828 |
| H | -1.70900 | 2.94230 | 0.31087 |
| O | 1.23212 | 3.47147 | -0.45240 |
| O | -0.46526 | 4.81814 | -0.16664 |

| **TB38** | | | |
| --- | --- | --- | --- |
| **Atom** | **X, Å** | **Y, Å** | **Z, Å** |
| C | -1.60538 | 0.24013 | -0.00140 |
| C | -0.37535 | -0.48920 | -0.12923 |
| N | 0.77614 | 0.23719 | -0.33739 |
| C | 0.74797 | 1.61756 | -0.43617 |
| C | -0.47066 | 2.20564 | -0.31629 |
| N | -1.64650 | 1.53573 | -0.09115 |
| N | -0.11797 | -1.78540 | -0.08492 |
| C | 1.75075 | -0.72641 | -0.38863 |
| N | 1.22212 | -1.92475 | -0.23933 |
| N | 3.91535 | 0.07160 | 0.35108 |
| O | 3.35209 | 0.74368 | 1.18974 |
| O | 5.09812 | -0.15017 | 0.23028 |
| N | -4.06367 | -0.24309 | 0.18406 |
| O | -4.79908 | -1.20802 | 0.31037 |
| O | -4.36859 | 0.91458 | 0.03103 |
| N | -2.69854 | -0.57618 | 0.24577 |
| N | 3.08549 | -0.46887 | -0.69226 |
| H | -0.55515 | 3.28237 | -0.38861 |
| H | -2.55721 | -1.57933 | 0.27995 |
| H | 1.68279 | 2.13316 | -0.58477 |
| H | 3.57831 | -1.24652 | -1.11863 |

| **TB56** | | | |
| --- | --- | --- | --- |
| **Atom** | **X, Å** | **Y, Å** | **Z, Å** |
| C | -1.29940 | -2.31729 | -0.60584 |
| C | -2.11611 | -1.29140 | -0.07387 |
| N | -1.49507 | -0.08735 | 0.27049 |
| C | -0.14028 | 0.10861 | 0.06949 |
| C | 0.55707 | -0.96929 | -0.41662 |
| N | -0.01018 | -2.16376 | -0.75396 |
| N | -3.40832 | -1.22443 | 0.19596 |
| C | -2.51168 | 0.68469 | 0.75109 |
| N | -3.64370 | 0.00804 | 0.70977 |
| N | 0.25003 | 2.45636 | -0.40153 |
| O | 1.01897 | 3.36857 | -0.19556 |
| O | -0.65832 | 2.40136 | -1.20635 |
| N | 2.88873 | -0.80536 | 0.36294 |
| O | 3.97445 | -1.29042 | 0.14003 |
| O | 2.54586 | -0.18794 | 1.36266 |
| H | -1.75032 | -3.26060 | -0.89311 |
| H | -2.38962 | 1.69576 | 1.09771 |
| N | 1.94885 | -0.90461 | -0.68928 |
| H | 2.25725 | -1.64807 | -1.30825 |
| N | 0.40728 | 1.33219 | 0.45909 |
| H | 1.33404 | 1.31090 | 0.87743 |

| **TB568** | | | |
| --- | --- | --- | --- |
| **Atom** | **X, Å** | **Y, Å** | **Z, Å** |
| C | -1.62645 | 0.63733 | -0.17087 |
| C | -1.42298 | -0.71758 | -0.56827 |
| N | -0.10585 | -1.18273 | -0.53661 |
| C | 0.95535 | -0.38043 | -0.15235 |
| C | 0.64438 | 0.92459 | 0.13152 |
| N | -0.62359 | 1.42061 | 0.13050 |
| N | -2.22211 | -1.65428 | -1.03840 |
| C | -0.19762 | -2.46100 | -1.00542 |
| N | -1.45035 | -2.73727 | -1.30682 |
| N | 2.66561 | -1.75776 | 0.88078 |
| O | 3.86385 | -1.90643 | 0.96341 |
| O | 1.79193 | -2.25790 | 1.55895 |
| N | 2.60080 | 2.33093 | -0.37291 |
| O | 3.01825 | 3.44571 | -0.15881 |
| O | 2.97080 | 1.55421 | -1.24197 |
| H | 0.64144 | -3.12741 | -1.10229 |
| N | 1.62940 | 1.85798 | 0.54337 |
| H | 1.24061 | 2.66295 | 1.02386 |
| N | 2.23708 | -0.92743 | -0.19869 |
| H | 2.98859 | -0.32381 | -0.52189 |
| N | -2.89138 | 1.22517 | -0.22635 |
| N | -3.98156 | 0.71845 | 0.55911 |
| H | -2.89811 | 2.23782 | -0.18017 |
| O | -3.84595 | -0.36580 | 1.06982 |
| O | -4.93576 | 1.46487 | 0.60048 |

| **TB58** | | | |
| --- | --- | --- | --- |
| **Atom** | **X, Å** | **Y, Å** | **Z, Å** |
| C | 1.39405 | -0.10989 | -0.10596 |
| C | 0.48477 | 1.00217 | -0.10097 |
| N | -0.85509 | 0.74111 | -0.30023 |
| C | -1.29916 | -0.57037 | -0.49762 |
| C | -0.34689 | -1.54382 | -0.47716 |
| N | 0.98946 | -1.33451 | -0.28910 |
| N | 0.69066 | 2.29318 | 0.06652 |
| C | -1.43956 | 1.97724 | -0.23265 |
| N | -0.52367 | 2.89966 | -0.01756 |
| N | -3.57259 | -0.72161 | 0.36532 |
| O | -4.74048 | -0.66246 | 0.03959 |
| O | -3.10017 | -0.73368 | 1.48063 |
| N | 3.85698 | -0.54215 | 0.16312 |
| O | 4.87415 | 0.09248 | 0.38136 |
| O | 3.73446 | -1.73085 | 0.00024 |
| N | 2.70528 | 0.26747 | 0.09961 |
| N | -2.65508 | -0.82181 | -0.72154 |
| H | -3.11669 | -0.55544 | -1.58315 |
| H | -0.65641 | -2.57118 | -0.62184 |
| H | -2.49732 | 2.15307 | -0.33394 |
| H | 2.91600 | 1.24825 | 0.24769 |

| **TB68** | | | |
| --- | --- | --- | --- |
| **Atom** | **X, Å** | **Y, Å** | **Z, Å** |
| C | -0.92300 | -0.49407 | -0.29302 |
| C | -1.08916 | 0.91970 | -0.21166 |
| N | 0.05711 | 1.65220 | 0.09100 |
| C | 1.29434 | 1.06775 | 0.26983 |
| C | 1.34277 | -0.28586 | 0.10170 |
| N | 0.24555 | -1.06011 | -0.16116 |
| N | -2.11296 | 1.73117 | -0.40820 |
| C | -0.37109 | 2.94790 | 0.06946 |
| N | -1.65522 | 2.99441 | -0.22758 |
| N | -3.15165 | -1.42608 | 0.17879 |
| O | -3.30119 | -0.58890 | 1.03572 |
| O | -3.87088 | -2.36271 | -0.09894 |
| N | 3.79808 | -0.66484 | 0.02722 |
| O | 4.60809 | -1.56147 | -0.12441 |
| O | 4.01813 | 0.53560 | 0.05117 |
| N | 2.49340 | -1.08377 | 0.24312 |
| H | 2.37896 | -2.06952 | 0.04452 |
| N | -1.99105 | -1.32486 | -0.65505 |
| H | 2.13865 | 1.69355 | 0.49701 |
| H | 0.26704 | 3.79357 | 0.26563 |
| H | -1.72391 | -2.25716 | -0.95027 |

| **TC35** | | | |
| --- | --- | --- | --- |
| **Atom** | **X, Å** | **Y, Å** | **Z, Å** |
| C | -2.77385 | -1.13063 | 0.14892 |
| C | -1.37285 | -1.35876 | 0.15786 |
| N | -0.52627 | -0.25660 | -0.05282 |
| C | -1.06918 | 1.01344 | -0.24740 |
| C | -2.43144 | 1.10627 | -0.24900 |
| N | -3.28434 | 0.05235 | -0.05388 |
| N | -0.65982 | -2.46012 | 0.29932 |
| C | 0.72484 | -0.81730 | -0.03638 |
| N | 0.64797 | -2.11566 | 0.17665 |
| N | 0.63733 | 2.48100 | 0.21968 |
| N | 2.94575 | -0.65383 | -0.19656 |
| H | -3.43734 | -1.97441 | 0.30695 |
| N | 1.86077 | -0.04900 | -0.23025 |
| N | -0.26959 | 2.12436 | -0.54840 |
| N | 3.99386 | -1.06583 | -0.19667 |
| N | 1.49015 | 2.94597 | 0.79204 |
| H | -2.87840 | 2.07778 | -0.41480 |

| **TC356** | | | |
| --- | --- | --- | --- |
| **Atom** | **X, Å** | **Y, Å** | **Z, Å** |
| C | 0.74873 | -2.44392 | 0.00011 |
| C | -0.55827 | -1.90440 | 0.00005 |
| N | -0.68988 | -0.50680 | -0.00003 |
| C | 0.42773 | 0.32089 | -0.00009 |
| C | 1.65085 | -0.32528 | -0.00006 |
| N | 1.80570 | -1.67839 | 0.00006 |
| N | -1.74900 | -2.47620 | 0.00009 |
| C | -2.04912 | -0.31914 | -0.00003 |
| N | -2.67143 | -1.48625 | 0.00004 |
| N | 1.02248 | 2.53754 | 0.00028 |
| N | 3.89383 | -0.08703 | -0.00007 |
| H | 0.87653 | -3.52046 | 0.00020 |
| N | 2.79655 | 0.48703 | -0.00016 |
| N | 0.13573 | 1.67188 | -0.00030 |
| N | 4.95017 | -0.48259 | -0.00004 |
| N | 1.66235 | 3.46700 | 0.00022 |
| N | -2.62522 | 0.93782 | -0.00009 |
| N | -3.86728 | 0.96547 | -0.00004 |
| N | -4.97772 | 1.15787 | -0.00001 |

| **TC3568** | | | |
| --- | --- | --- | --- |
| **Atom** | **X, Å** | **Y, Å** | **Z, Å** |
| C | -1.21386 | -1.45300 | -0.00002 |
| C | 0.21050 | -1.53571 | -0.00001 |
| N | 0.91774 | -0.32726 | 0.00002 |
| C | 0.26906 | 0.90763 | 0.00003 |
| C | -1.11037 | 0.85201 | 0.00003 |
| N | -1.83096 | -0.30172 | 0.00000 |
| N | 1.03938 | -2.55918 | -0.00003 |
| C | 2.22826 | -0.73598 | 0.00002 |
| N | 2.29720 | -2.05595 | -0.00001 |
| N | 4.41905 | -0.34798 | 0.00003 |
| N | 0.69637 | 3.16289 | -0.00007 |
| N | 1.11854 | 1.99911 | 0.00008 |
| N | 3.28345 | 0.15678 | 0.00004 |
| N | 0.51962 | 4.27822 | -0.00026 |
| N | 5.50558 | -0.64622 | 0.00003 |
| N | -1.90198 | -2.66222 | -0.00005 |
| N | -3.14353 | -2.58723 | -0.00006 |
| N | -4.26578 | -2.67105 | -0.00006 |
| N | -1.79499 | 2.07736 | 0.00008 |
| N | -3.03226 | 2.03480 | 0.00010 |
| N | -4.15623 | 2.13398 | 0.00011 |

| **TC358** | | | |
| --- | --- | --- | --- |
| **Atom** | **X, Å** | **Y, Å** | **Z, Å** |
| C | -2.12911 | -0.16331 | 0.01296 |
| C | -0.85435 | -0.81364 | 0.05699 |
| N | 0.27803 | -0.00385 | -0.10473 |
| C | 0.15489 | 1.37853 | -0.28819 |
| C | -1.11435 | 1.87393 | -0.32737 |
| N | -2.24524 | 1.12226 | -0.17898 |
| N | -0.50492 | -2.07505 | 0.19484 |
| C | 1.30930 | -0.90606 | -0.06119 |
| N | 0.85134 | -2.12814 | 0.12010 |
| N | 3.48487 | -1.39589 | -0.14402 |
| N | 2.19204 | 2.28186 | 0.27027 |
| N | 1.26668 | 2.19119 | -0.55213 |
| N | 2.62572 | -0.49932 | -0.19847 |
| N | 3.11377 | 2.48136 | 0.88842 |
| N | 4.36681 | -2.09544 | -0.12000 |
| N | -3.23847 | -0.98993 | 0.17894 |
| N | -4.34665 | -0.42713 | 0.12873 |
| N | -5.40819 | -0.05511 | 0.10672 |
| H | -1.24888 | 2.93558 | -0.48702 |

| **TC36** | | | |
| --- | --- | --- | --- |
| **Atom** | **X, Å** | **Y, Å** | **Z, Å** |
| C | 1.14801 | 1.83267 | 0.00001 |
| N | 2.10899 | 0.94901 | 0.00002 |
| C | -0.21939 | 1.46205 | 0.00000 |
| N | -0.50102 | 0.09427 | 0.00000 |
| C | 1.77997 | -0.38384 | 0.00003 |
| C | 0.49728 | -0.85652 | 0.00002 |
| C | -1.86200 | 0.04786 | -0.00000 |
| N | -1.34538 | 2.16027 | -0.00001 |
| N | -2.36851 | 1.27083 | -0.00001 |
| H | 1.40802 | 2.88616 | 0.00000 |
| H | 0.22053 | -1.89906 | 0.00002 |
| N | 2.81865 | -1.32839 | 0.00005 |
| N | 3.97634 | -0.88517 | -0.00002 |
| N | 5.07454 | -0.62804 | -0.00006 |
| N | -2.51506 | -1.17304 | -0.00000 |
| N | -3.75668 | -1.13906 | -0.00001 |
| N | -4.87641 | -1.26358 | -0.00001 |

| **TC368** | | | |
| --- | --- | --- | --- |
| **Atom** | **X, Å** | **Y, Å** | **Z, Å** |
| C | 1.02369 | -1.01702 | -0.00005 |
| C | -0.40555 | -1.02693 | -0.00003 |
| N | -1.02389 | 0.22122 | 0.00001 |
| C | -0.31681 | 1.40917 | 0.00001 |
| C | 1.04345 | 1.29216 | -0.00002 |
| N | 1.70962 | 0.09662 | -0.00005 |
| N | -1.30855 | -1.99155 | -0.00004 |
| C | -2.35144 | -0.08480 | 0.00000 |
| N | -2.52839 | -1.39540 | -0.00003 |
| N | -4.48642 | 0.57699 | 0.00004 |
| N | 3.02901 | 2.35912 | 0.00001 |
| N | 1.79573 | 2.47773 | -0.00003 |
| N | -3.29491 | 0.92840 | 0.00003 |
| N | 4.15655 | 2.40102 | -0.00000 |
| N | -5.60054 | 0.41072 | 0.00004 |
| N | 1.64417 | -2.26160 | -0.00012 |
| N | 2.88929 | -2.25171 | 0.00002 |
| N | 4.00440 | -2.39639 | 0.00018 |
| H | -0.86256 | 2.33847 | 0.00005 |

| **TC38** | | | |
| --- | --- | --- | --- |
| **Atom** | **X, Å** | **Y, Å** | **Z, Å** |
| C | -1.71322 | -0.05353 | 0.00001 |
| C | -0.34312 | -0.47302 | 0.00001 |
| N | 0.60704 | 0.54655 | 0.00000 |
| C | 0.25569 | 1.88562 | 0.00000 |
| C | -1.07258 | 2.16242 | 0.00000 |
| N | -2.05714 | 1.20661 | 0.00001 |
| N | 0.24621 | -1.65305 | 0.00001 |
| C | 1.79349 | -0.12625 | 0.00000 |
| N | 1.59047 | -1.42928 | 0.00000 |
| N | 4.02885 | -0.09856 | -0.00001 |
| N | -3.85134 | -0.71027 | -0.00000 |
| N | -2.66213 | -1.07512 | 0.00001 |
| N | 2.98709 | 0.57777 | -0.00000 |
| N | -4.96132 | -0.52739 | -0.00002 |
| N | 5.04898 | -0.57654 | -0.00001 |
| H | -1.41133 | 3.19043 | 0.00000 |
| H | 1.05284 | 2.61297 | -0.00000 |

| **TC56** | | | |
| --- | --- | --- | --- |
| **Atom** | **X, Å** | **Y, Å** | **Z, Å** |
| C | 0.97520 | -2.02562 | 0.00006 |
| C | 1.92346 | -0.97485 | 0.00001 |
| N | 1.43093 | 0.33071 | -0.00002 |
| C | 0.07638 | 0.60125 | 0.00001 |
| C | -0.75687 | -0.50161 | -0.00001 |
| N | -0.31000 | -1.78694 | 0.00004 |
| N | 3.24809 | -0.95383 | 0.00000 |
| C | 2.55256 | 1.11320 | -0.00004 |
| N | 3.62714 | 0.34780 | -0.00003 |
| N | -1.37782 | 2.37726 | 0.00003 |
| N | -2.88582 | -1.24738 | 0.00001 |
| H | 1.31731 | -3.05417 | 0.00011 |
| H | 2.53112 | 2.18891 | -0.00005 |
| N | -2.14045 | -0.25811 | -0.00009 |
| N | -0.20922 | 1.95903 | 0.00002 |
| N | -3.67000 | -2.05796 | -0.00003 |
| N | -2.35184 | 2.94530 | 0.00004 |

| **TC568** | | | |
| --- | --- | --- | --- |
| **Atom** | **X, Å** | **Y, Å** | **Z, Å** |
| C | -1.56860 | -0.20663 | -0.00007 |
| C | -1.03075 | -1.52929 | -0.00005 |
| N | 0.35632 | -1.63386 | 0.00005 |
| C | 1.19276 | -0.52904 | 0.00016 |
| C | 0.55936 | 0.69644 | -0.00001 |
| N | -0.79174 | 0.84628 | -0.00009 |
| N | -1.57809 | -2.73178 | -0.00009 |
| C | 0.58717 | -2.98194 | 0.00008 |
| N | -0.56021 | -3.63058 | -0.00000 |
| N | 3.42574 | 0.00344 | -0.00002 |
| N | 0.80639 | 2.93958 | 0.00026 |
| H | 1.57140 | -3.41665 | 0.00018 |
| N | 1.37466 | 1.83936 | -0.00022 |
| N | 2.53978 | -0.86417 | 0.00059 |
| N | 0.41462 | 3.99752 | 0.00022 |
| N | 4.36064 | 0.63527 | -0.00062 |
| N | -2.95571 | -0.09984 | -0.00007 |
| N | -3.41897 | 1.05438 | -0.00008 |
| N | -3.97501 | 2.03288 | -0.00005 |

| **TC58** | | | |
| --- | --- | --- | --- |
| **Atom** | **X, Å** | **Y, Å** | **Z, Å** |
| C | -1.41974 | -0.14406 | 0.00001 |
| C | -0.68677 | 1.08624 | 0.00003 |
| N | 0.70185 | 0.98231 | -0.00011 |
| C | 1.33936 | -0.24502 | -0.00030 |
| C | 0.54317 | -1.35654 | -0.00041 |
| N | -0.82027 | -1.30550 | -0.00022 |
| N | -1.04519 | 2.35556 | 0.00021 |
| C | 1.13420 | 2.28128 | -0.00002 |
| N | 0.09972 | 3.09347 | 0.00016 |
| N | 3.38795 | -1.20260 | -0.00005 |
| N | -3.44343 | -1.10088 | 0.00009 |
| N | -2.80967 | -0.03174 | 0.00020 |
| N | 2.73415 | -0.15177 | -0.00053 |
| N | -4.14109 | -1.98429 | 0.00007 |
| N | 4.10448 | -2.07594 | 0.00087 |
| H | 0.98631 | -2.34514 | -0.00067 |
| H | 2.17286 | 2.56329 | -0.00007 |

| **TC68** | | | |
| --- | --- | --- | --- |
| **Atom** | **X, Å** | **Y, Å** | **Z, Å** |
| C | 0.19103 | 0.88090 | -0.00005 |
| N | -0.85051 | 0.08851 | -0.00004 |
| C | 1.52847 | 0.37680 | -0.00001 |
| N | 1.66633 | -1.00664 | 0.00001 |
| C | -0.65366 | -1.26336 | 0.00001 |
| C | 0.57707 | -1.85595 | 0.00001 |
| C | 3.01741 | -1.19767 | 0.00003 |
| N | 2.71666 | 0.95881 | 0.00000 |
| N | 3.64048 | -0.03424 | 0.00003 |
| H | 0.74086 | -2.92157 | 0.00003 |
| N | -1.77737 | -2.10652 | 0.00003 |
| N | -2.88861 | -1.55866 | 0.00000 |
| N | -3.95755 | -1.19759 | -0.00004 |
| N | 0.05751 | 2.26523 | -0.00013 |
| N | -1.10811 | 2.70257 | -0.00002 |
| N | -2.09715 | 3.23786 | 0.00016 |
| H | 3.48539 | -2.16812 | 0.00005 |

| **TD35** | | | |
| --- | --- | --- | --- |
| **Atom** | **X, Å** | **Y, Å** | **Z, Å** |
| C | -3.22044 | -0.38643 | 0.41546 |
| N | -3.31291 | 0.92056 | 0.42579 |
| C | -2.02852 | -1.05569 | 0.05022 |
| C | -2.21913 | 1.64443 | 0.05475 |
| C | -1.02462 | 1.09514 | -0.33298 |
| N | -0.92240 | -0.28832 | -0.29574 |
| N | -1.73374 | -2.35213 | -0.04414 |
| C | 0.04454 | -1.22767 | -0.61589 |
| N | -0.46224 | -2.44734 | -0.45475 |
| H | -4.08596 | -0.97727 | 0.69597 |
| O | -0.02656 | 1.82261 | -0.84743 |
| O | 1.26672 | -0.92841 | -0.97750 |
| N | 1.18651 | 2.07001 | 0.15632 |
| N | 2.36663 | -1.20782 | 0.26219 |
| O | 2.01027 | 2.73774 | -0.35522 |
| O | 1.06980 | 1.55320 | 1.22196 |
| O | 1.88162 | -1.59838 | 1.26482 |
| O | 3.45319 | -0.96536 | -0.12044 |
| H | -2.31827 | 2.72271 | 0.03751 |

| **TD356** | | | |
| --- | --- | --- | --- |
| **Atom** | **X, Å** | **Y, Å** | **Z, Å** |
| C | 0.90302 | 2.73646 | -0.00040 |
| N | 1.83558 | 1.93613 | -0.45098 |
| C | -0.39466 | 2.26692 | 0.31155 |
| C | 1.55243 | 0.62188 | -0.60502 |
| C | 0.32506 | 0.04068 | -0.32473 |
| N | -0.65062 | 0.91429 | 0.14322 |
| N | -1.48459 | 2.88531 | 0.76505 |
| C | -1.96497 | 0.77212 | 0.54604 |
| N | -2.44627 | 1.96050 | 0.89914 |
| H | 1.13227 | 3.78788 | 0.13402 |
| O | 2.51003 | -0.16598 | -1.15799 |
| O | 0.01444 | -1.21842 | -0.52965 |
| N | 3.74554 | -0.35644 | -0.24850 |
| N | 0.14950 | -2.15207 | 0.87053 |
| O | 4.63551 | -0.84147 | -0.85661 |
| O | 3.60870 | -0.02070 | 0.88462 |
| O | 0.60903 | -1.56769 | 1.78884 |
| O | -0.24393 | -3.23595 | 0.65511 |
| O | -2.59191 | -0.37955 | 0.61217 |
| N | -3.34004 | -0.75982 | -0.81353 |
| O | -3.91388 | -1.77994 | -0.70354 |
| O | -3.16814 | 0.03327 | -1.67457 |

| **TD3568** | | | |
| --- | --- | --- | --- |
| **Atom** | **X, Å** | **Y, Å** | **Z, Å** |
| C | -2.13607 | 0.06316 | -0.21491 |
| N | -1.70817 | 1.25508 | 0.08490 |
| C | -1.24659 | -0.98990 | -0.58155 |
| C | -0.38296 | 1.50574 | 0.03913 |
| N | 0.10821 | -0.69919 | -0.61808 |
| N | -1.46245 | -2.25035 | -0.92896 |
| C | 0.67950 | -1.88638 | -1.03976 |
| N | -0.26444 | -2.80166 | -1.19822 |
| O | 0.03866 | 2.72720 | 0.44116 |
| O | 1.95658 | -2.04443 | -1.33635 |
| N | -0.04490 | 3.80416 | -0.69206 |
| N | 2.86126 | -2.42173 | -0.06544 |
| O | 0.24219 | 4.86744 | -0.26800 |
| O | -0.36470 | 3.39404 | -1.76008 |
| O | 2.31239 | -2.31131 | 0.98432 |
| O | 3.94406 | -2.72550 | -0.40931 |
| O | -3.47138 | -0.14928 | -0.25610 |
| N | -3.98894 | -1.14913 | 0.82169 |
| O | -5.11471 | -1.42409 | 0.61064 |
| O | -3.20467 | -1.43719 | 1.66571 |
| C | 0.59114 | 0.57501 | -0.31390 |
| O | 1.86985 | 0.80188 | -0.32291 |
| N | 2.52542 | 0.87567 | 1.30048 |
| O | 1.70489 | 0.78273 | 2.13806 |
| O | 3.68533 | 1.03158 | 1.22983 |

| **TD358** | | | |
| --- | --- | --- | --- |
| **Atom** | **X, Å** | **Y, Å** | **Z, Å** |
| C | -2.11174 | -0.23946 | -0.04382 |
| N | -2.08467 | 0.90648 | 0.57853 |
| C | -0.91987 | -0.91421 | -0.45900 |
| C | -0.88250 | 1.48462 | 0.84403 |
| N | 0.27699 | -0.28875 | -0.16849 |
| N | -0.70707 | -2.05818 | -1.09940 |
| C | 1.22435 | -1.14161 | -0.69859 |
| N | 0.62393 | -2.19496 | -1.23664 |
| O | 1.50332 | 1.43213 | 0.84669 |
| O | 2.51654 | -0.88800 | -0.70024 |
| N | 2.18964 | 2.31129 | -0.39991 |
| N | 3.25703 | -1.45058 | 0.65066 |
| O | 3.28494 | 2.62731 | -0.11127 |
| O | 1.47385 | 2.46891 | -1.32968 |
| O | 2.52498 | -1.95520 | 1.43209 |
| O | 4.41205 | -1.23571 | 0.59286 |
| O | -3.26431 | -0.85012 | -0.33165 |
| N | -4.49924 | 0.00697 | 0.18165 |
| O | -4.90625 | 0.73761 | -0.64786 |
| O | -4.83609 | -0.30835 | 1.26549 |
| C | 0.32745 | 0.92968 | 0.51660 |
| H | -0.89458 | 2.43142 | 1.36855 |

| **TD36** | | | |
| --- | --- | --- | --- |
| **Atom** | **X, Å** | **Y, Å** | **Z, Å** |
| C | 0.95917 | 2.28358 | 0.34415 |
| N | 1.95711 | 1.50446 | 0.01923 |
| C | -0.38911 | 1.88654 | 0.15924 |
| C | 1.67673 | 0.27921 | -0.50021 |
| C | 0.41898 | -0.21332 | -0.69755 |
| N | -0.61412 | 0.62466 | -0.36868 |
| N | -1.55124 | 2.49911 | 0.38640 |
| C | -1.99145 | 0.53731 | -0.45323 |
| N | -2.53043 | 1.65952 | 0.01177 |
| H | 1.17026 | 3.26260 | 0.76088 |
| O | 2.73659 | -0.47592 | -0.92232 |
| O | -2.60955 | -0.51025 | -0.94004 |
| N | 3.57585 | -1.02875 | 0.22849 |
| N | -3.11090 | -1.55221 | 0.26575 |
| O | 4.59060 | -1.48829 | -0.17318 |
| O | 3.09370 | -0.93840 | 1.31485 |
| O | -2.76793 | -1.23382 | 1.35242 |
| O | -3.72994 | -2.43649 | -0.20243 |
| H | 0.19216 | -1.18470 | -1.11050 |

| **TD368** | | | |
| --- | --- | --- | --- |
| **Atom** | **X, Å** | **Y, Å** | **Z, Å** |
| C | 1.18312 | 1.05927 | -0.22503 |
| N | 1.85118 | 0.04224 | 0.22651 |
| C | -0.20022 | 0.96780 | -0.56289 |
| C | 1.21904 | -1.15543 | 0.34195 |
| N | -0.79631 | -0.27053 | -0.39677 |
| N | -1.08194 | 1.84816 | -1.02021 |
| C | -2.10213 | -0.06535 | -0.80970 |
| N | -2.25550 | 1.20307 | -1.16534 |
| O | 1.93045 | -2.18592 | 0.88293 |
| O | -2.99520 | -1.02408 | -0.83959 |
| N | 3.01063 | -2.73563 | -0.06622 |
| N | -3.95164 | -1.02613 | 0.52767 |
| O | 3.72993 | -3.47912 | 0.50679 |
| O | 2.94742 | -2.35804 | -1.19361 |
| O | -3.63161 | -0.22895 | 1.34023 |
| O | -4.78873 | -1.84520 | 0.42404 |
| O | 1.85016 | 2.22034 | -0.45117 |
| N | 1.41448 | 3.38245 | 0.46926 |
| O | 1.89563 | 4.39541 | 0.10462 |
| O | 0.71481 | 3.07827 | 1.38130 |
| C | -0.09292 | -1.36791 | 0.02746 |
| H | -0.60049 | -2.31754 | 0.10062 |

| **TD38** | | | |
| --- | --- | --- | --- |
| **Atom** | **X, Å** | **Y, Å** | **Z, Å** |
| C | -1.75771 | 0.79439 | -0.24700 |
| N | -1.71757 | 1.99839 | 0.23395 |
| C | -0.58238 | 0.06530 | -0.59086 |
| C | -0.49762 | 2.59888 | 0.39735 |
| C | 0.68634 | 1.99176 | 0.10623 |
| N | 0.62388 | 0.70914 | -0.38118 |
| N | -0.37850 | -1.15008 | -1.08861 |
| C | 1.56597 | -0.20948 | -0.80861 |
| N | 0.95256 | -1.31273 | -1.21455 |
| O | 2.85438 | 0.04009 | -0.80560 |
| O | -2.61337 | -1.05267 | 1.37574 |
| N | 3.59882 | -0.56248 | 0.55367 |
| N | -3.34884 | -0.89252 | 0.45192 |
| O | 4.76105 | -0.39061 | 0.48225 |
| O | 2.85712 | -1.04546 | 1.33891 |
| O | -2.97826 | 0.23915 | -0.50404 |
| O | -4.35000 | -1.41812 | 0.10846 |
| H | -0.51060 | 3.60933 | 0.78443 |
| H | 1.66309 | 2.43854 | 0.22061 |

| **TD56** | | | |
| --- | --- | --- | --- |
| **Atom** | **X, Å** | **Y, Å** | **Z, Å** |
| C | 1.18368 | -2.24314 | 0.19059 |
| N | -0.06553 | -2.06204 | -0.15307 |
| C | 2.13373 | -1.19222 | 0.15233 |
| C | -0.46911 | -0.82597 | -0.53131 |
| C | 0.35368 | 0.28857 | -0.59465 |
| N | 1.68473 | 0.06425 | -0.25290 |
| N | 3.42995 | -1.14136 | 0.41814 |
| C | 2.79861 | 0.84920 | -0.20943 |
| N | 3.83317 | 0.13269 | 0.19334 |
| H | 2.81107 | 1.89205 | -0.47613 |
| H | 1.50115 | -3.23092 | 0.50621 |
| O | -1.75084 | -0.68233 | -0.95462 |
| O | 0.00170 | 1.49253 | -0.97340 |
| N | -2.80600 | -0.99999 | 0.13202 |
| N | -0.68100 | 2.39850 | 0.29773 |
| O | -3.87696 | -1.11094 | -0.35735 |
| O | -2.40131 | -1.05805 | 1.24812 |
| O | -0.84249 | 1.78106 | 1.29240 |
| O | -0.89046 | 3.49470 | -0.07041 |

| **TD568** | | | |
| --- | --- | --- | --- |
| **Atom** | **X, Å** | **Y, Å** | **Z, Å** |
| C | -1.50591 | 0.66048 | -0.18194 |
| N | -0.44871 | 1.32890 | 0.17512 |
| C | -1.43596 | -0.68974 | -0.64183 |
| C | 0.76425 | 0.73907 | 0.08731 |
| C | 0.97817 | -0.55365 | -0.37038 |
| N | -0.17111 | -1.26807 | -0.69833 |
| N | -2.34931 | -1.55080 | -1.04463 |
| C | -0.40421 | -2.52802 | -1.17189 |
| N | -1.69619 | -2.69765 | -1.37117 |
| O | 1.83635 | 1.43143 | 0.54023 |
| O | 2.14141 | -1.13224 | -0.49743 |
| N | 2.36584 | 2.47761 | -0.48885 |
| N | 2.72734 | -1.74955 | 1.00147 |
| O | 3.21749 | 3.13685 | -0.00435 |
| O | 1.85559 | 2.44090 | -1.56154 |
| O | 2.02137 | -1.51186 | 1.91557 |
| O | 3.75523 | -2.28652 | 0.82204 |
| O | -2.69708 | 1.30536 | -0.18741 |
| N | -3.72937 | 0.73744 | 0.82476 |
| O | -4.79402 | 1.20151 | 0.62320 |
| O | -3.29112 | -0.01821 | 1.62995 |
| H | 0.37079 | -3.25161 | -1.35828 |

| **TD58** | | | |
| --- | --- | --- | --- |
| **Atom** | **X, Å** | **Y, Å** | **Z, Å** |
| C | -1.33630 | -0.54374 | -0.68384 |
| N | -0.62484 | -1.59469 | -0.97022 |
| C | -0.75581 | 0.72878 | -0.40201 |
| N | 0.63348 | 0.80384 | -0.43407 |
| C | 1.40747 | -0.30789 | -0.75598 |
| C | 0.72947 | -1.47528 | -1.01182 |
| N | -1.26887 | 1.90768 | -0.10413 |
| C | 0.90204 | 2.11022 | -0.14107 |
| N | -0.22599 | 2.76307 | 0.05971 |
| H | 1.89435 | 2.52577 | -0.09953 |
| H | 1.29728 | -2.36208 | -1.26341 |
| O | 2.71907 | -0.16164 | -0.80793 |
| N | 3.45991 | -0.50763 | 0.65899 |
| O | 2.73712 | -0.97920 | 1.46749 |
| O | 4.60062 | -0.22600 | 0.60119 |
| O | -2.69026 | -0.65590 | -0.74075 |
| N | -3.39056 | -0.48831 | 0.61954 |
| O | -4.55926 | -0.40609 | 0.47402 |
| O | -2.67665 | -0.49917 | 1.57130 |

| **TD68** | | | |
| --- | --- | --- | --- |
| **Atom** | **X, Å** | **Y, Å** | **Z, Å** |
| C | -0.65087 | -0.49396 | -0.25087 |
| N | 0.55555 | -0.79890 | 0.10914 |
| C | -1.13715 | 0.84974 | -0.24880 |
| C | 1.39763 | 0.20679 | 0.47088 |
| C | 1.05886 | 1.52693 | 0.49642 |
| N | -0.23687 | 1.83613 | 0.15529 |
| N | -2.30204 | 1.38711 | -0.54737 |
| C | -0.96265 | 2.99343 | 0.06736 |
| N | -2.18292 | 2.72851 | -0.34606 |
| O | 2.63999 | -0.15166 | 0.91242 |
| O | -1.45486 | -1.48706 | -0.71928 |
| N | 3.56775 | -0.61372 | -0.21884 |
| N | -2.64479 | -1.80612 | 0.20036 |
| O | 4.55386 | -1.10009 | 0.21930 |
| O | 3.18001 | -0.40399 | -1.32581 |
| O | -2.61018 | -1.30485 | 1.27913 |
| O | -3.39573 | -2.54192 | -0.33630 |
| H | 1.73378 | 2.31952 | 0.78122 |
| H | -0.57015 | 3.96827 | 0.30537 |

# Part B. Molecular electrostatic potential maps for the 5-H-imidazo[4,5-c]-pyridazine and 1,2,4-triazolo[4,3-a]-pyrazine derivatives

**Table S2.** Molecular electrostatic potential maps for the 5-H-imidazo[4,5-c]-pyridazine derivatives, mapped on the electron density surface at $0.001 a. u$. Red regions indicate electron-rich corresponding to negative MEP values, while the blue region indicates electron-deficient corresponding to positive MEP values.

|  | | | | | |
| --- | --- | --- | --- | --- | --- |
|  | Position | $-NO_{2}$ | $-NHNO_{2}$ | $-N_{3}$ | $-ONO_{2}$ |
| 5-H-imidazo[4,5-c]-pyridazine | 34 |  |  |  |  |
|  | 35 |  |  |  |  |

| 5-H-imidazo[4,5-c]-pyridazine | Position | $-NO_{2}$ | $-NHNO_{2}$ | $-N_{3}$ | $-ONO_{2}$ |
| --- | --- | --- | --- | --- | --- |
|  | 36 |  |  |  |  |
|  | 45 |  |  |  |  |
|  | 46 |  |  |  |  |
|  | 56 |  |  |  |  |

|  | Position | $-NO_{2}$ | $-NHNO_{2}$ | $-N_{3}$ | $-ONO_{2}$ |
| --- | --- | --- | --- | --- | --- |
| 5-H-imidazo[4,5-c]-pyridazine | 345 |  |  |  |  |
|  | 346 |  |  |  |  |
|  | 356 |  |  |  |  |
|  | 456 |  |  |  |  |

|  | Position | $-NO_{2}$ | $-NHNO_{2}$ | $-N_{3}$ | $-ONO_{2}$ |
| --- | --- | --- | --- | --- | --- |
| 5-H-imidazo[4,5-c]-pyridazine | 3456 |  |  |  |  |

**Table S3.** Molecular electrostatic potential maps for the 1,2,4-triazolo[4,3-a]-pyrazine derivatives, mapped on the electron density surface at $0.001 a. u$. Red regions indicate electron-rich corresponding to negative MEP values, while the blue region indicates electron-deficient corresponding to positive MEP values.

|  | | | | | |
| --- | --- | --- | --- | --- | --- |
| 1,2,4-triazolo[4,3-a]-pyrazine | Position | $-NO_{2}$ | $-NHNO_{2}$ | $-N_{3}$ | $-ONO_{2}$ |
|  | 35 |  |  |  |  |
|  | 36 |  |  |  |  |

|  | Position | $-NO_{2}$ | $-NHNO_{2}$ | $-N_{3}$ | $-ONO_{2}$ |
| --- | --- | --- | --- | --- | --- |
| 1,2,4-triazolo[4,3-a]-pyrazine | 38 |  |  |  |  |
|  | 56 |  |  |  |  |
|  | 58 |  |  |  |  |
|  | 68 |  |  |  |  |

|  | Position | $-NO_{2}$ | $-NHNO_{2}$ | $-N_{3}$ | $-ONO_{2}$ |
| --- | --- | --- | --- | --- | --- |
| 1,2,4-triazolo[4,3-a]-pyrazine | 356 |  |  |  |  |
|  | 358 |  |  |  |  |
|  | 368 |  |  |  |  |
|  | 568 |  |  |  |  |

|  | Position | $-NO_{2}$ | $-NHNO_{2}$ | $-N_{3}$ | $-ONO_{2}$ |
| --- | --- | --- | --- | --- | --- |
| 1,2,4-triazolo[4,3-a]-pyrazine | 3568 |  |  |  |  |

# Part C. Computed aromaticity index $\boldsymbol{NICS}\left( \mathbf{1} \right)_{\boldsymbol{zz}}$ of the 6-member ring and 5-member ring of of 5-H-imidazo[4,5-c]-pyridazine and 1,2,4-triazolo[4,3-a]-pyrazine derivatives

**Figure S1.** A plot of the computed aromaticity index $NICS\left( 1 \right)_{zz}$ of the 6-member ring. Predicted densities ($\rho$), in g cm-3, maximum heat of detonation ($Q_{MAX}$) in kcal kg⁻¹, detonation velocities ($D$) in km s⁻¹, and pressure ($P_{CJ}$), in GPa, of 5-H-imidazo[4,5-c]-pyridazine derivatives (a) $-NO_{2}$; (b) $-NHNO_{2}$; (c) $-N_{3}$ and (d) $-ONO_{2}$.

**Figure S2.** A plot of the computed aromaticity index $NICS\left( 1 \right)_{zz}$ of the 5-member ring. Predicted densities ($\rho$), in g cm-3, maximum heat of detonation ($Q_{MAX}$) in kcal kg⁻¹, detonation velocities ($D$) in km s⁻¹, and pressure ($P_{CJ}$), in GPa, of 5-H-imidazo[4,5-c]-pyridazine derivatives (a) $-NO_{2}$; (b) $-NHNO_{2}$; (c) $-N_{3}$ and (d) $-ONO_{2}$.

**Figure S3.** A plot of the computed aromaticity index $NICS\left( 1 \right)_{zz}$ of the 6-member ring. Predicted densities ($\rho$), in g cm-3, maximum heat of detonation ($Q_{MAX}$) in kcal kg⁻¹, detonation velocities ($D$) in km s⁻¹, and pressure ($P_{CJ}$), in GPa, of 1,2,4-triazolo[4,3-a]-pyrazine derivatives: (a) $-NO_{2}$; (b) $-NHNO_{2}$; (c) $-N_{3}$ and (d) $-ONO_{2}$.

**Figure S4.** A plot of the computed aromaticity index $NICS\left( 1 \right)_{zz}$ of the 5-member ring. Predicted densities ($\rho$), in g cm-3, maximum heat of detonation ($Q_{MAX}$) in kcal kg⁻¹, detonation velocities ($D$) in km s⁻¹, and pressure ($P_{CJ}$), in GPa, of 1,2,4-triazolo[4,3-a]-pyrazine derivatives: (a) $-NO_{2}$; (b) $-NHNO_{2}$; (c) $-N_{3}$ and (d) $-ONO_{2}$.

# Part D. Identification of Trigger Bonds in the designed compounds through Mayer Bond Order

**Table S4.** Calculated Mayer Bond Order of the designed compounds.

| Compounds | Explosophore | Trigger Bond | Position | MBO  (minimum) | **MBO** **(N1-N2)** | **MBO** **(N2-N3)** |
| --- | --- | --- | --- | --- | --- | --- |
| IA34 | $-NO₂$ | $C-NO₂$ | R4 | 0.789 | - | - |
| IA35 | $-NO₂$ | $C-NO₂$ | R3 | 0.740 | - | - |
| IA36 | $-NO₂$ | $C-NO₂$ | R6 | 0.739 | - | - |
| IA45 | $-NO₂$ | $C-NO₂$ | R4 | 0.773 | - | - |
| IA46 | $-NO₂$ | $C-NO₂$ | R6 | 0.740 | - | - |
| IA56 | $-NO₂$ | $C-NO₂$ | R6 | 0.748 | - | - |
| IA345 | $-NO₂$ | $C-NO₂$ | R3 | 0.747 | - | - |
| IA346 | $-NO₂$ | $C-NO₂$ | R6 | 0.737 | - | - |
| IA356 | $-NO₂$ | $C-NO₂$ | R3 | 0.738 | - | - |
| IA456 | $-NO₂$ | $N-NO₂$ | R5 | 0.620 | - | - |
| IA3456 | $-NO₂$ | $N-NO₂$ | R5 | 0.606 | - | - |
| IB34 | $-NHNO₂$ | $C-NHNO₂$ | R4 | 0.930 | - | - |
| IB35 | $-NHNO₂$ | $HN-NO₂$ | R5 | 0.794 | - | - |
| IB36 | $-NHNO₂$ | $C-NHNO₂$ | R3 | 0.944 | - | - |
| IB45 | $-NHNO₂$ | $HN-NO₂$ | R5 | 0.808 | - | - |
| IB46 | $-NHNO₂$ | $C-NHNO₂$ | R4 | 0.917 | - | - |
| IB56 | $-NHNO₂$ | $HN-NO₂$ | R5 | 0.816 | - | - |
| IB345 | $-NHNO₂$ | $HN-NO₂$ | R5 | 0.784 | - | - |
| IB346 | $-NHNO₂$ | $C-NHNO₂$ | R4 | 0.935 | - | - |
| IB356 | $-NHNO₂$ | $HN-NO₂$ | R5 | 0.812 | - | - |
| IB456 | $-NHNO₂$ | $HN-NO₂$ | R5 | 0.818 | - | - |
| IB3456 | $-NHNO₂$ | $HN-NO₂$ | R5 | 0.816 | - | - |
| IC34 | $-N₃$ | $C-N₃$ | R3 | 1.020 | 1.367 | 2.261 |
| IC35 | $-N₃$ | $N-N₃$ | R5 | 0.940 | 1.230 | 2.245 |
| IC36 | $-N₃$ | $C-N₃$ | R3 | 1.034 | 1.317 | 2.254 |
| IC45 | $-N₃$ | $N-N₃$ | R5 | 0.939 | 1.242 | 2.246 |
| IC46 | $-N₃$ | $C-N₃$ | R4 | 1.102 | 1.360 | 2.217 |
| IC56 | $-N₃$ | $N-N₃$ | R5 | 0.955 | 1.246 | 2.245 |
| IC345 | $-N₃$ | $N-N₃$ | R5 | 0.941 | 1.243 | 2.247 |
| IC346 | $-N₃$ | $C-N₃$ | R3 | 1.018 | 1.368 | 2.261 |
| IC356 | $-N₃$ | $N-N₃$ | R5 | 0.958 | 1.244 | 2.246 |
| IC456 | $-N₃$ | $N-N₃$ | R5 | 0.963 | 1.244 | 2.252 |
| IC3456 | $-N₃$ | $N-N₃$ | R5 | 0.964 | 1.245 | 2.252 |
| ID34 | $-ONO₂$ | $O-N$ | R4 | 0.710 | - | - |
| ID35 | $-ONO₂$ | $O-N$ | R5 | 0.575 | - | - |
| ID36 | $-ONO₂$ | $O-N$ | R6 | 0.653 | - | - |
| ID45 | $-ONO₂$ | $O-N$ | R5 | 0.573 | - | - |
| ID46 | $-ONO₂$ | $O-N$ | R6 | 0.654 | - | - |
| ID56 | $-ONO₂$ | $O-N$ | R5 | 0.573 | - | - |
| ID345 | $-ONO₂$ | $O-N$ | R5 | 0.567 | - | - |
| ID346 | $-ONO₂$ | $O-N$ | R6 | 0.641 | - | - |
| ID356 | $-ONO₂$ | $O-N$ | R5 | 0.571 | - | - |
| ID456 | $-ONO₂$ | $O-N$ | R5 | 0.561 | - | - |
| ID3456 | $-ONO₂$ | $O-N$ | R5 | 0.560 | - | - |
| TA35 | $-NO₂$ | $C-NO₂$ | R3 | 0.774 | - | - |
| TA36 | $-NO₂$ | $C-NO₂$ | R6 | 0.756 | - | - |
| TA38 | $-NO₂$ | $C-NO₂$ | R8 | 0.750 | - | - |
| TA56 | $-NO₂$ | $C-NO₂$ | R6 | 0.782 | - | - |
| TA58 | $-NO₂$ | $C-NO₂$ | R8 | 0.762 | - | - |
| TA68 | $-NO₂$ | $C-NO₂$ | R8 | 0.747 | - | - |
| TA356 | $-NO₂$ | $C-NO₂$ | R6 | 0.753 | - | - |
| TA358 | $-NO₂$ | $C-NO₂$ | R8 | 0.758 | - | - |
| TA368 | $-NO₂$ | $C-NO₂$ | R8 | 0.748 | - | - |
| TA568 | $-NO₂$ | $C-NO₂$ | R8 | 0.762 | - | - |
| TA3568 | $-NO₂$ | $C-NO₂$ | R6 | 0.746 | - | - |
| TB35 | $-NHNO₂$ | $HN-NO₂$ | R3 | 0.826 | - | - |
| TB36 | $-NHNO₂$ | $HN-NO₂$ | R3 | 0.880 | - | - |
| TB38 | $-NHNO₂$ | $HN-NO₂$ | R3 | 0.882 | - | - |
| TB56 | $-NHNO₂$ | $HN-NO₂$ | R5 | 0.892 | - | - |
| TB58 | $-NHNO₂$ | $HN-NO₂$ | R5 | 0.885 | - | - |
| TB68 | $-NHNO₂$ | $HN-NO₂$ | R8 | 0.880 | - | - |
| TB356 | $-NHNO₂$ | $HN-NO₂$ | R5 | 0.857 | - | - |
| TB358 | $-NHNO₂$ | $HN-NO₂$ | R3 | 0.823 | - | - |
| TB368 | $-NHNO₂$ | $HN-NO₂$ | R3 | 0.876 | - | - |
| TB568 | $-NHNO₂$ | $HN-NO₂$ | R8 | 0.872 | - | - |
| TB3568 | $-NHNO₂$ | $HN-NO₂$ | R3 | 0.835 | - | - |
| TC35 | $-N₃$ | $C-N₃$ | R5 | 1.041 | 1.302 | 2.240 |
| TC36 | $-N₃$ | $C-N₃$ | R6 | 1.055 | 1.331 | 2.241 |
| TC38 | $-N₃$ | $C-N₃$ | R3 | 1.069 | 1.334 | 2.250 |
| TC56 | $-N₃$ | $C-N₃$ | R6 | 1.029 | 1.388 | 2.245 |
| TC58 | $-N₃$ | $C-N₃$ | R5 | 1.079 | 1.334 | 2.212 |
| TC68 | $-N₃$ | $C-N₃$ | R6 | 1.052 | 1.333 | 2.235 |
| TC356 | $-N₃$ | $C-N₃$ | R6 | 1.027 | 1.389 | 2.244 |
| TC358 | $-N₃$ | $C-N₃$ | R5 | 1.037 | 1.301 | 2.240 |
| TC368 | $-N₃$ | $C-N₃$ | R6 | 1.056 | 1.331 | 2.235 |
| TC568 | $-N₃$ | $C-N₃$ | R6 | 1.035 | 1.385 | 2.239 |
| TC3568 | $-N₃$ | $C-N₃$ | R6 | 1.033 | 1.386 | 2.239 |
| TD35 | $-ONO₂$ | $O-N$ | R3 | 0.568 | - | - |
| TD36 | $-ONO₂$ | $O-N$ | R3 | 0.576 | - | - |
| TD38 | $-ONO₂$ | $O-N$ | R3 | 0.585 | - | - |
| TD56 | $-ONO₂$ | $O-N$ | R5 | 0.537 | - | - |
| TD58 | $-ONO₂$ | $O-N$ | R5 | 0.565 | - | - |
| TD68 | $-ONO₂$ | $O-N$ | R8 | 0.728 | - | - |
| TD356 | $-ONO₂$ | $O-N$ | R5 | 0.554 | - | - |
| TD358 | $-ONO₂$ | $O-N$ | R5 | 0.570 | - | - |
| TD368 | $-ONO₂$ | $O-N$ | R3 | 0.579 | - | - |
| TD568 | $-ONO₂$ | $O-N$ | R5 | 0.515 | - | - |
| TD3568 | $-ONO₂$ | $O-N$ | R5 | 0.490 | - | - |
